# School‐Based Interventions for Reducing Disciplinary School Exclusion. An Updated Systematic Review

**DOI:** 10.1002/cl2.70063

**Published:** 2025-10-22

**Authors:** Sara Valdebenito, Hannah Gaffney, Maria Jose Arosemena‐Burbano, Sydney Hitchcock, Darrick Jolliffe, Alex Sutherland

**Affiliations:** ^1^ Institute of Criminology University of Cambridge Cambridge UK; ^2^ Clare Hall College Cambridge UK; ^3^ Department of Security and Crime Science University College London London UK; ^4^ Department of Social Policy and Intervention University of Oxford Oxford UK

## Abstract

School exclusion—commonly referred to as suspension—is a disciplinary response employed by school authorities to address student misbehaviour. Typically, it involves temporary removal from regular teaching or, in more serious cases, complete removal from the school premises. A substantial body of research has associated exclusion with adverse developmental outcomes. In response, various school‐based interventions have been developed to reduce exclusion rates. While some programmes have shown promising effects, the evidence on their effectiveness remains inconclusive. This mixed‐methods systematic review and multi‐level meta‐analysis updates the previous review by Valdebenito et al. (2018), which included literature published between 1980 and 2015. The present update extends the evidence base by including studies until 2022. The primary aim of this review was to assess the effectiveness of school‐based interventions in reducing disciplinary exclusions, with secondary aims focused on related behavioural outcomes including conduct problems, delinquency, and substance use. Systematic searches conducted between November and December 2022 yielded over 11,000 references for quantitative studies. Following title and abstract screening, 777 records were reviewed at full text by two independent coders. Thirty‐two studies met the inclusion criteria for meta‐analysis, comprising 2765 effect sizes from 67 primary evaluations (1980–2022) and representing approximately 394,242 students. Meta‐analysis was conducted using a multilevel random‐effects model with robust variance estimation to account for the nested structure of the data. Quantitative impact evaluations were eligible if they used a randomised controlled or quasi‐experimental design, included both a control group and pre/post‐test data, and used statistical methods to minimise selection bias (e.g., propensity score matching or matched cohort design). Studies were excluded if they exhibited substantial baseline differences between treatment and control groups. The qualitative synthesis explored implementation barriers and facilitators based on nine UK‐based process evaluations, identified through searches completed in September 2023. Process evaluations were included if they focused on the perceptions of stakeholders—teachers, students, or school leadership—within UK schools. Data collection followed two stages: initial selection based on titles, abstracts, and keywords, followed by full‐text review. Two independent coders applied inclusion criteria, extracted data, and resolved discrepancies with the principal investigators. All steps were documented to inform the PRISMA flow chart. To evaluate interventions reducing school exclusions, we conducted a multilevel meta‐analysis using robust variance estimation. We explored heterogeneity via meta‐regression (e.g., gender, intervention type), conducted sensitivity analyses for outliers and correlation structures, and assessed quality data using the EPOC, ROBIN‐I and CASP checklist for methodological quality. Findings indicated that school‐based interventions were associated with a small but statistically significant reduction in school exclusion (standardised mean difference [SMD] = 0.104; 95% CI: 0.04 to 0.17; *p* < 0.001). Compared with the original 2018 review, which reported a slightly larger effect size, this update benefits from a broader evidence base and more advanced statistical modelling. However, the results for secondary behavioural outcomes were more limited: effects on conduct problems and delinquency were negligible or non‐significant, and the impact on substance use was small and not statistically significant. Risk of bias was assessed using the Cochrane EPOC 2 tool (Higgins and Green 2011) for randomised controlled trials and ROBINS‐I (Sterne et al. 2016) for quasi‐experimental designs. Randomised studies generally exhibited lower risk of bias, while quasi‐experimental studies showed greater variability in quality. Four major themes emerged from the analysis. First, *intervention format* mattered: flexible, collaborative, and well‐structured interventions facilitated implementation, while outdated materials or content misaligned with local context impeded delivery. Second, *consistency* in school policies and practice enabled smoother implementation, whereas inconsistency acted as a barrier. Third, *staff buy‐in*—particularly among senior leaders—was essential for successful implementation, although resistance from more experienced staff was noted. Finally, *perceived effectiveness* played a motivational role: visible improvements in pupil behaviour supported continued engagement with the intervention. In summary, the updated review finds that school‐based interventions can modestly but significantly reduce school exclusions. While more serious disciplinary sanctions such as permanent exclusions and out‐of‐school suspensions appear less responsive, in‐school exclusion shows greater potential for reduction. Impacts on other behavioural outcomes remain limited. These findings suggest that targeted, context‐sensitive interventions supported by strong implementation strategies and whole‐school engagement are most likely to achieve sustained reductions in school exclusion.

## 1 Plain Language Summary

*School‐based interventions have a small impact on reducing school exclusion*.

### 1.1 The Review in Brief

The meta‐analysis found that school‐based interventions slightly reduced school exclusion. This effect, though small, was significant. Results varied by intervention type, with less impact on severe exclusions and mixed outcomes for behavioural issues. Larger sample sizes and independent evaluations are needed for more precise findings.

### 1.2 What Is This Review About?

School exclusion, a severe disciplinary measure, entails removing students from regular classes or school premises. In the United Kingdom, 346,279 students were suspended for a fixed period, and 4168 were permanently excluded in the autumn term of 2023/24, marking an increase from previous years (Department for Education 2025). The predominant reason for these exclusions was persistent disruptive behaviour. Exclusion is associated with negative outcomes in behaviour, academic performance, and future social inclusion. Excluded students are at higher risk for criminal behaviour, academic failure, and social exclusion (Arnez and Condry 2021; Cohen et al. 2023). Vulnerable groups, including minority ethnic students and those with special educational needs, are disproportionately impacted. Therefore, effective interventions are essential to reduce exclusion rates and address these disparities.

### 1.3 What Is the Aim of This Review?

The present mixed‐methods review aims to systematically examine the effectiveness of various school‐based interventions in reducing disciplinary school exclusions, conduct problems, and drug use. It will analyse differential effects based on participant and study characteristics and identify implementation barriers and facilitators for successful programme outcomes.

### 1.4 What Are the Main Findings of This Review?

#### 1.4.1 What Studies Are Included?

The review included 275 effect sizes from 67 studies, combining data from Valdebenito et al. (2018) and recent findings, covering 1980 to 2022. This evidence includes 394,242 students involved in school‐based interventions to reduce school exclusions through RCT or quasi‐experimental evaluations.

### 1.5 What Do the Findings of This Review Mean?

The meta‐analysis found that school‐based interventions significantly reduce school exclusion, though the effect size is small and varies by context. These interventions are more effective for general suspensions than for severe disciplinary actions like expulsions. Secondary outcomes related to behaviour showed mixed results. Qualitative findings highlighted factors influencing success, including intervention format, policy consistency, and staff buy‐in. Positive behavioural changes and structured, flexible programmes facilitated success, while outdated materials and context mismatches posed barriers. The study underscores the need for tailored approaches and independent evaluations to enhance intervention effectiveness.

### 1.6 How Up‐to‐Date Is This Review?

The review authors searched for studies up to 2023. This Campbell Systematic Review was published in 2025.

## 2 Background

### 2.1 Description of the Problem or Condition

Schools play a vital role in the lives of children and young people, extending far beyond the provision of academic instruction (Sanders et al. 2020). In addition to families, schools represent the primary institution through which young people are socialised into broader societal norms. They are introduced to concepts such as discipline, authority, codes of conduct, and the consequences associated with their transgression (Fisher et al. 2019; Maimon et al. 2012).

In a manner analogous to the justice system, schools adopt a spectrum of punitive and non‐punitive responses to manage behavioural infractions. Among the more severe disciplinary measures is school exclusion, also referred to as suspension. This sanction is typically imposed by school leadership in response to student misbehaviour and involves the temporary removal of the pupil from regular educational activities. During this period, the student may be barred from attending classes or, in more serious cases, from being on school premises altogether. In the most extreme instances, exclusion may result in permanent expulsion or transfer to an alternative educational setting (Department for Education 2024).

In the United Kingdom, the most recent available official data suggest that for the autumn term 2023/24, a total of 346,279 (rate of 4.13 per 1000) students were suspended or excluded from school for a fixed period, and 4168 (rate of 0.05 per 1000) were permanently excluded (Department for Education 2025). These figures represent an increase from the numbers of exclusions in English schools for the comparable period in the previous year (i.e., spring term 2022/23: *N* = 263,900) and are now higher than the final term before the COVID‐19 pandemic and subsequent school closures (Department for Education 2025). Click or tap here to enter text. In the UK policy context, other available options include ‘managed moves’, whereby a child who is at risk of exclusion from one school may be moved to another or moved to a pupil referral unit/alternate provision (Thomson 2019). ‘Off rolling’ is another way in which schools may deal with children who are at risk of permanent exclusion without using official channels (Timpson 2019). Parents of a child at risk of exclusion may be pressured into removing their child from one school before an exclusion takes place, and while this is not necessarily illegal, official bodies have deemed it an unacceptable practice (Owen 2019).

A range of behaviours can lead to a student being excluded from school. In England, ‘persistent disruptive behaviour’ was cited as the reason for 51% of all fixed‐term exclusions and 38% of permanent exclusions during the autumn term of the 2023/24 academic year (Department for Education 2025). These behaviours are often of serious concern. For instance, in the spring term of 2023/24, 23% of permanent exclusions were attributed to verbal abuse or threats directed at school staff, while 13% resulted from physical assaults against other pupils (Department for Education 2025). Regarding frequency, most suspended pupils (63%) received a single suspension, while 31% were suspended two to four times, and a further 6% were excluded on five or more occasions during the same period.

The empirical literature has consistently shown that exclusion from school is associated with adverse outcomes across behavioural, academic, and social domains. In terms of behavioural consequences (Cohen et al. 2023), they observed that both in‐school and out‐of‐school exclusions were linked to lower prosocial behaviour, diminished emotional regulation, increased disruptive conduct, and greater concentration difficulties among a predominantly Black sample of school‐aged children. Scholars such as Arnez and Condry (2021) have drawn attention to the ‘school‐to‐prison pipeline’, whereby excluded pupils appear at greater risk of engaging in delinquent or criminal activity, a trajectory also noted by McAra and McVie (2010).

Academically, early exclusion has been associated with diminished educational attainment. Andrew and Blake (2023) reported a link between exclusion and poorer performance as students progressed through school. Leban and Masterson (2022) found that suspension by age 12 significantly raised the likelihood of dropping out, even after adjusting for confounding factors such as ethnicity and exposure to childhood adversity. Similarly, Chu and Ready (2018) demonstrated that students excluded in Year 9 were less likely to complete secondary education within 4–6 years compared to their non‐excluded peers.

Social outcomes are also affected. Excluded youth are more likely to become categorised as NEET (Not in Education, Employment, or Training) by ages 19–20, and face a higher risk of unemployment and lower earnings by ages 25–26 (Madia et al. 2022). Beyond labour market participation, school exclusions may also have civic implications. Kupchik and Catlaw (2015), for instance, found that individuals suspended during their school years were subsequently less likely to participate in elections, indicating a negative effect on political engagement.

While school exclusion—whether temporary or permanent—may, in certain instances, be justified for safeguarding or disciplinary reasons, the breadth of evidence highlights its potential for lasting consequences.

Despite the potential long‐term and life‐altering impact of being excluded from school, at times the decision to exclude a child from school, either permanently or temporarily, may be necessary. These decisions often involve a series of correlated consequences and difficult decisions for school authorities. In Edward Timpson's review of school exclusion practices in England, the authors assert that:

Schools must be calm and safe places, and it is right that we fully support head teachers in using exclusion where this is appropriate. Head teachers considering exclusion have a tough choice to make, having to weigh the profound implications that it can have on a young person's life with the interests and needs of pupils and staff in the wider school community.

(Timpson 2019, 3)

Not all children face an equal risk of being excluded from school. In England, exclusion rates are disproportionately higher among boys, students from certain ethnic minority backgrounds, those eligible for free school meals, and pupils with special educational needs (Department for Education 2025). This disproportionality has been widely documented and may function as a mechanism through which broader social inequalities—such as those related to socioeconomic status or criminal justice involvement—are perpetuated (Demie 2021; Skiba et al. 2014; Towl and Hemphill 2016). Children with impairing psychopathologies or additional learning needs are also overrepresented among those excluded (Parker et al. 2015).

In this review, we use the term ‘school exclusion’ to refer to the removal of a pupil from the provision of education, encompassing both temporary and permanent exclusions, unless otherwise specified. Temporary exclusions include fixed‐period suspensions, whether implemented in‐school (e.g., isolation rooms) or out‐of‐school, whereas permanent exclusion entails the complete removal of a pupil from their educational institution (Department for Education 2025).

Although school exclusion is sometimes framed as a behavioural intervention intended to address disruptive conduct, its effectiveness in this role remains questionable. Sutherland and Eisner (2014) highlighted the lack of empirical evidence supporting the use of exclusion as a deterrent. Indeed, exclusion may lead to repeat suspensions: Theriot et al. (2010) found that students who received in‐ or out‐of‐school suspensions were more likely to be excluded again in the future.

Given these concerns, our updated review considers a range of school‐based interventions aimed at reducing exclusion. These interventions span all educational levels, from primary to secondary school, and may also include multi‐system approaches, provided a substantial component is delivered within the school environment. Eligible programmes include school‐based mental health services such as individual counselling (Toth et al. 2022), as well as structural reforms aimed at developing more equitable disciplinary policies (Gregory and Skiba 2019).

### 2.2 How the Intervention Might Work

Previous research has indicated the need for intervention programmes that address both student behaviour and the school environment to reduce the number of students who are excluded (Theriot et al. 2010). Therefore, the theories of change underlying interventions to reduce school exclusions are likely to focus on individual‐ or school‐level change, or both. As such, interventions to prevent or reduce the frequency of school exclusions may work through various mechanisms. These mechanisms will depend on the type of intervention programme implemented.

Valdebenito et al. (2018) noted that theoretical foundations were not commonly reported in the primary evaluations of interventions to reduce school exclusions. It was more common for evaluations to report a set of components or activities that were implemented. Valdebenito et al. (2018) categorised interventions using this limited information and reported that the majority were based on a cognitive behavioural framework or an ecological systems theory.

On the system or school‐level, interventions may aim to reduce school exclusions by targeting the ‘school climate’. This possible mechanism of change involves creating a school climate that promotes supportive relationships and encourages positive behaviour (Scottish Government 2017).

At the individual‐level, interventions may aim to directly change students' behaviour, particularly disruptive, violent, or aggressive behaviour, to reduce the risk of a child being excluded from school. Interventions may also target risk factors for exclusion, such as truancy (Keppens and Spruyt 2020) or specific behaviours directly, for example using anger management or cognitive behavioural therapy techniques (Feindler and Engel 2011).

### 2.3 Why It Is Important to Do This Review

There are multiple justifications for undertaking the present mixed‐methods systematic review and multi‐level meta‐analysis. Most notably, a considerable body of research has demonstrated a strong association between school exclusion and a range of negative life outcomes, including increased involvement in crime and violence. The exclusion of pupils from school has been identified as a critical turning point in a young person's developmental trajectory and is recognised as a significant risk factor for continued offending (McAra and McVie 2010).

The concept of the ‘school‐to‐prison pipeline’—a term used to describe the pathway from school exclusion to contact with the criminal justice system—has gained traction in recent years. National‐level evidence from England shows the magnitude of this issue. (Cathro et al. 2023), for instance, reported that nearly half (approximately 50%) of young people aged 15–17 years serving custodial sentences had experienced at least one school exclusion. This finding reflects a broader concern regarding the potential long‐term consequences of exclusionary disciplinary practices.

International research has produced similar findings. In New Zealand, Sanders et al. (2020) examined the relationship between school exclusion and contact with the criminal justice system. School exclusion was operationalised through three indicators: being ‘stood down’, fixed‐term exclusions, and permanent exclusions. Criminal justice involvement was measured through arrest, court appearances, and custodial sentences over a 12‐month period. Their results indicated both a direct association between exclusion and criminal justice system contact, as well as an indirect relationship mediated by delinquent behaviours such as theft, vandalism, and aggression.

Given these findings, there is a clear rationale for conducting a rigorous synthesis of evidence on the effectiveness of interventions aimed at reducing school exclusion. Reducing exclusionary practices may not only improve educational outcomes but also serve as a preventative strategy against youth crime and violence. Thus, school‐based interventions intended to prevent exclusion can be framed as a form of crime prevention—an angle we seek to explore further in the present review.

Another key rationale for this review lies in its status as an update to a previous Campbell systematic review and meta‐analysis (Valdebenito et al. 2018). The original searches for that review were conducted in 2015 and focused exclusively on randomised controlled trials (RCTs). It represented the first comprehensive synthesis of the effectiveness of school‐based interventions to reduce exclusions. However, as the body of relevant literature has grown in both size and methodological diversity, there is a need to revisit and expand upon that initial evidence base.

The present review addresses this gap by incorporating studies published between 2016 and 2022, as well as broadening inclusion criteria to encompass high‐quality quasi‐experimental evaluations and qualitative process evaluations. This expanded scope aims not only to enhance our understanding of the effectiveness of such interventions, but also to explore the contextual and implementation factors that may support or hinder their success. As such, this review represents a timely and significant contribution to the field of school discipline and youth prevention research.

### 2.4 Previous Reviews and Meta‐Analyses

Despite the considerable body of evidence linking school exclusion to a range of adverse developmental outcomes, there remains a notable paucity of consistent and rigorous research on the effectiveness of interventions aimed at reducing exclusionary practices. Three systematic reviews have addressed this topic to varying extents (Gage and Stevens 2018; Mielke and Farrington 2021; Valdebenito et al. 2018) each contributing important yet distinct perspectives to the literature.

The earliest of these, Valdebenito et al. (2018), conducted a comprehensive systematic review and meta‐analysis of RCTs evaluating school‐based interventions designed to reduce disciplinary exclusions. Drawing on 37 studies published from 1980 onwards, this review identified a small but statistically significant reduction in school exclusion in the short term (SMD = 0.30; 95% CI [0.20, 0.41]; *p* < 0.001). However, the effect was not sustained in longer‐term follow‐ups. The analysis further revealed that certain types of interventions—such as academic skills enhancement, counselling, mentoring, and teacher training—were associated with more favourable outcomes. The authors also noted that interventions were more effective in reducing expulsions and in‐school exclusions than out‐of‐school suspensions. Importantly, methodological factors such as the role of the evaluator influenced effect size estimates, with studies involving independent evaluators tending to report smaller effects.

In the same year, Gage and Stevens (2018) published a narrower review focusing exclusively on Schoolwide Positive Behaviour Interventions and Supports (SWPBIS), a widely adopted approach in school settings. Despite the intervention's broad implementation across approximately 23,000 schools in the United States, the review identified only four studies meeting the threshold for experimental or quasi‐experimental evaluation. This highlights an important gap between widespread adoption of school‐based behavioural frameworks and the availability of robust evidence regarding their efficacy in reducing exclusion.

More recently, Mielke and Farrington (2021) conducted a meta‐analysis restricted to school‐based interventions targeting reductions in fixed‐term exclusions (suspensions) and arrests. Fourteen studies were included, and although findings suggested some small (i.e., both below *d* = 0.1) reductions in both outcomes, the weighted mean effect sizes were not statistically significant. While this review offered a focused lens on the relationship between exclusion and justice system involvement, its limited scope and small number of studies reduce the generalisability of its conclusions.

Together, these reviews indicate that while some school‐based interventions show promise in mitigating exclusionary disciplinary practices, the strength of the evidence base remains limited. The majority of available evaluations originate from the United States, and high‐quality research in other contexts remains scarce. Furthermore, long‐term effects are often not sustained, underscoring the need for more rigorous and comprehensive evaluations of intervention effectiveness across diverse educational settings.

## 3 Objectives

The primary goal of the present mixed methods review is to systematically examine the available evidence for the effectiveness of different types of school‐based interventions for reducing disciplinary school exclusion. Quantitative evidence will help to understand the overall size of the impact, as well as the factors that better explain it. Qualitative evidence will help to understand better how these programmes may work, and what factors aid or hinder implementation and success.

The research questions underlying the quantitative review are as follows:

1. Do school‐based programmes reduce the use of exclusionary sanctions in schools?
2. Are some school‐based approaches more effective than others in reducing exclusionary sanctions?
3. Do participants' characteristics (e.g., age, sex or ethnicity) affect the impact of school‐based programmes on exclusionary sanctions in schools?
4. Do characteristics of the interventions, implementation, and methodology affect the impact of school‐based programmes on exclusionary sanctions in schools?
5. Do school‐based programmes have an impact on reducing the involvement of children and young people in crime and violence?
6. Do participants' characteristics (e.g., age, gender, ethnicity) affect the impact of school‐based programmes on crime and violence?

If sufficient data are available, we will compare different approaches (e.g., school‐wide management, classroom management, restorative justice, cognitive‐behavioural interventions) and identify those that could potentially demonstrate larger effects. We will also (potentially) run analysis controlling for characteristics of *participants* (e.g., age, ethnicity, level of risk); *interventions* (e.g., theoretical bases, components); *implementation* (e.g., facilitators' training, doses, quality); and *methodology* (e.g., research design).

The research questions underlying the qualitative review are defined as follows:

7. What are the barriers and facilitators to implementation of interventions to reduce school exclusions?
8. What are the barriers and facilitators to implementation of interventions to reduce the involvement of children and young people in crime and violence?

## 4 Methods

The present section describes the methods involved in this systematic review and meta‐analysis. The chapter explicitly defines the criteria for the selection of studies, search methods for the identification of studies, and methods for data collection and data analysis. The methods outlined here are in line with the protocol for the current review (Valdebenito et al. 2023). Any deviations from the protocol are explained and justified in Section 4.5.

### 4.1 Criteria for Considering Studies for This Review

A PICOS framework was used to guide the inclusion criteria for our quantitative assessment of the impact of school‐based interventions to reduce and prevent children being excluded from school. As such, searches were performed using keywords related to the: (i) population; (ii) intervention; (iii) comparison; (iv) outcomes; and (v) study design.

Inclusion criteria for our qualitative evidence synthesis of UK process evaluations to understand the barriers and facilitators to implementing interventions to reduce school exclusions is informed by a PerSpecTIF framework (Booth et al. 2019).

#### 4.1.1 Types of Studies

The present review included experimental, quasi‐experimental and process evaluations of school‐based interventions. Experimental and quasi‐experimental designs (QEDs) were used to address research questions (RQs) 1–6 and process evaluations (i.e., those that use qualitative methodologies) were used to answer RQs 7 and 8.

##### 4.1.1.1 Research Questions 1–6: Impact Evaluations

To be eligible for inclusion, experimental and QEDs had to comprise at least one treatment group, where participants took part in an intervention or were exposed to a treatment, and at least one comparison condition. Evaluations that used multiple treatment arms were eligible for inclusion and suitable comparison conditions included: (i) waitlist control groups (i.e., where the comparison agree to participate and receive the intervention or treatment following completion of the evaluation); (ii) treatment‐as‐usual control (i.e., where no intervention is implemented and schools continue to use their existing disciplinary policies or procedures related to exclusion); (iii) placebo control groups (i.e., where the comparison schools received an alternative treatment or intervention); or (iv) no treatment control groups (i.e., where nothing is done or implemented). Studies that used alternative treatment groups were considered for inclusion but were excluded if the comparison condition took part in a conceptually similar intervention or treatment. For example, there are many evaluations that compare outcomes for different schools based on the level of implementation of Positive Behavioural Interventions and Supports (PBIS) and only report a comparison of high‐implementation to low‐implementation schools (Grasley‐Boy et al. 2022). Such comparisons were excluded due to the lack of an appropriate control group.

Eligible experimental evaluations used randomised controlled designs (RCTs) where participants were randomly assigned to treatment and control conditions. Evaluations that randomly assigned individuals or clusters of individuals (e.g., classes or schools) to experimental conditions were also eligible for inclusion. Included evaluations used different methods to assign treatment and control groups randomly. For example, random number allocation (e.g., Augustine et al. 2018), or block randomisation (Bergman and Chan 2017).

QEDs were included in the present review if, in the absence of random assignment to experimental and control conditions, adequate matching procedures were used to reduce possible selection biases. Eligible QEDs used matching procedures such as propensity score matching. Matching procedures must have incorporated both behavioural risk factors (e.g., suspension rate, number of office discipline referrals, or prevalence of disruptive behaviour) and demographic factors known to be risk factors for exclusion from school (e.g., minority ethnicity or male students). In addition, QEDs must have measured school exclusion outcomes pre‐ and post‐intervention.

Regarding difference‐in‐differences designs, they were not excluded a priori. Rather, they were eligible for inclusion provided they met the same criteria as other QEDs—specifically, the presence of a clearly defined comparison group, pre‐ and post‐intervention measurements, and evidence of baseline comparability or adjustment for potential confounders. Some DiD studies may have been excluded if they did not meet these minimum thresholds or if they lacked sufficient statistical reporting to enable effect size computation.

Other types of QEDs were excluded from the review, including two‐group post‐test only designs, single‐group pre‐post‐test designs, and repeated measures panel designs where outcomes are measured at numerous time points both before and after implementation of an intervention.

##### 4.1.1.2 Research Questions 7 and 8: Process Evaluations

Process evaluations were eligible for inclusion in the present review to examine RQs 7 and 8. Our definition of a process evaluation refers to a qualitative study that examines the perspectives and views of participants who took part in an evaluation of an intervention. Eligible process evaluations could have been conducted alongside a quantitative impact evaluation (i.e., ‘trial siblings’ [Noyes et al. 2016]), or independently of an impact evaluation. We placed no restrictions on the type of process evaluations that were eligible for inclusion in the review, with the exception that only those employing qualitative methodologies would be included.

As such, purely quantitative process evaluations are excluded. For example, those that quantify and assess implementation fidelity or dosage of an intervention. This is because the purpose of our qualitative evidence synthesis is to understand the perspectives of students, teachers, and school staff on the facilitators and barriers to implementation of interventions to reduce school exclusions.

#### 4.1.2 Types of Participants

##### 4.1.2.1 Research Questions 1–6: Impact Evaluations

The present review examines the impact of interventions implemented and evaluated in schools. Specifically, ‘mainstream’ schools. Therefore, evaluations conducted in schools that only enrol students with special educational needs and disabilities (SEND)^1^ or alternative educational settings (e.g., alternative provision) for students with behavioural needs are excluded. As per the previous review, this is to ensure that the results are generalisable to all mainstream schools (Valdebenito et al. 2018). If an evaluation included participants with SEND status that are enroled in a mainstream school, they were included.

No restrictions were placed on any demographic factors of participants. As such, for the quantitative section of the review, no studies were excluded on basis of participants' nationality, culture, or socio‐economic background.

Evaluations conducted in either primary or secondary schools were eligible for inclusion in the quantitative review, and as such, participants were typically aged between 4 and 18 years. However, no restrictions or specific criteria on participant age were incorporated in our inclusion/exclusion criteria as outlined in the protocol. The ‘school‐age’ of participants may vary internationally, and so it is possible that participants aged 3–19 years are included in the review. However, evaluations conducted in pre‐school, kindergarten, nursery or further and higher education institutions (e.g., community college, University, training colleges) were not included.

Participants from other types of schools, for example, charter schools, private schools, alternative provision, or pupil referral units, were excluded. Alternative provision settings and pupil referral units (PPUs) are defined as educational settings whereby students have been removed from ‘normal’, or mainstream, education, typically due to their behaviour, special educational needs, social or emotional needs. Alternative provision and PPUs in the United Kingdom are also used for students considered at‐risk of exclusion from mainstream schools (Department for Education 2024, 2025). Private schools are those in which attendance is accompanied by a fee, and in comparison to public schools, they are not required to follow the national curriculum.^2^ Charter schools are a concept unique to the United States and are described in the literature as ‘relatively autonomous’ schools that are set up to implement novel and ambitious learning programmes to enhance achievement (Bulkley and Fisler 2003).

##### 4.1.2.2 Research Questions 7 and 8: Process Evaluations

Similar inclusion criteria relating to the age of students and the types of schools were employed in our qualitative evidence synthesis. However, in addition to school‐age participants, eligible process evaluations may have included adult participants. For example, process evaluations that present the perspectives of teachers, school staff, school leaders or administrators were eligible for inclusion. Perspectives of parents were considered, but not if these were the only group that participated in the process evaluation.

#### 4.1.3 Types of Interventions

For the purpose of both our quantitative assessment of impact evaluations and the qualitative synthesis of process evaluations, evaluations of school‐based interventions were included. We include interventions defined as school‐based: that is, delivered on school premises, or supported by schools with at least one component implemented in the school setting. In the present review, we include interventions explicitly aimed at preventing/reducing school exclusion or those measuring exclusion as an outcome. This definition encompasses a wide array of intervention programmes. For example, included interventions may have involved teacher training (e.g., Kendziora et al. 2019), mentoring (e.g., MacIver et al. 2017), mindfulness (e.g., Meadows 2018), or teen courts (e.g., Smokowski et al. 2020).

In addition to interventions that take place solely in schools, we also included evaluations of multi‐system interventions with the provision that at least one aspect of the intervention involved schools. For example, interventions that worked with families and/or parents to support children considered at‐risk of exclusion (e.g., Mason et al. 2016) were eligible for inclusion. Furthermore, interventions that involved multi‐disciplinary teams were eligible for inclusion if one (or more) of the components involved the child's school (e.g., Thompson et al. 2021; Weist et al. 2022).

#### 4.1.4 Types of Outcomes

##### 4.1.4.1 Research Questions 1–6: Impact Evaluations

The primary outcome of interest in this review was school exclusion. This included: permanent exclusion, fixed‐term exclusion, in‐school suspension, out‐of‐school suspension, and suspension. As previously outlined, our operational definition of school exclusion is in line with the previous review and refers to incidents whereby a student is removed from regular teaching and is not permitted to be present in the classroom or on school property (Kugley et al. 2017; Valdebenito et al. 2018).

To be included in the present review, evaluations were required to report the effect of an intervention on any of the aforementioned school exclusion outcomes. We also extracted information relating to any additional outcomes reported by evaluations. These included conduct problems, violence, delinquent behaviour, antisocial behaviour and substance use.

Included evaluations reported outcomes as either dichotomous (i.e., number of students excluded as a percentage of the total sample) or continuous (i.e., mean number of suspensions per student/school). We included outcomes measured using official school records/data or self‐, teacher‐, or parents‐report.

##### 4.1.4.2 Research Questions 7 and 8: Process Evaluations

Process evaluations (i.e., qualitative) were eligible for inclusion in our qualitative evidence synthesis if they reported findings on the perspectives of participants on the facilitators and barriers to intervention implementation. We defined a facilitator to implementation as any factor, component, or strategy that participants perceived as aiding the implementation of the intervention. These may also be referred to as enablers in the literature. We defined a barrier to implementation as any factor, component, or strategy that participants perceived as hindering or restricting the implementation of the intervention. This includes elements of the intervention that participants viewed as difficult, negative, harmful, or challenging.

#### 4.1.5 Duration of Follow‐up

##### 4.1.5.1 Research Questions 1–6: Impact Evaluations

We included multiple effect sizes from evaluations in the present review, and no restriction was placed on the duration of the follow‐up. Thus, baseline to post‐intervention follow‐ups were included, along with baseline to additional follow‐up timepoints. We estimated the baseline to post‐intervention effect size as the difference between a baseline measure of the outcome (where available) and the first point of data collection following completion of the intervention. Additional follow‐ups were recorded, as the number of months from baseline to the time of data collection.

##### 4.1.5.2 Research Questions 7 and 8: Process Evaluations

We did not restrict the inclusion of process evaluations based on the duration of follow‐up. Eligible process evaluations reported findings at the end of an intervention, several months after the completion of an intervention, or during the implementation of an intervention. This was to capture perspectives of participants that not only took part in an intervention, but also those who completed it, and those who possibly dropped out during the evaluation. It was our view that the experiences and opinions of those who decided not to continue participation would be invaluable to further our understanding of the barriers and facilitators to implementation.

#### 4.1.6 Types of Settings

##### 4.1.6.1 Research Questions 1–6: Impact Evaluations

We did not place any restriction on the setting or location of impact evaluations. However, to be included, studies must have had a title, abstract, or keywords in English for the purpose of screening. We were able to include studies written in English or Spanish.

##### 4.1.6.2 Research Questions 7 and 8: Process Evaluations

Our synthesis of process evaluations included only studies conducted in the United Kingdom.

### 4.2 Search Methods for Identification of Studies

Systematic searches for the present review of impact evaluations were undertaken in November–December 2022. Systematic searches for qualitative process evaluations took place in September 2023.^3^ Exact dates for each search are outlined in Appendix S1A. Searches for impact evaluations and process evaluations were conducted independently of one another on electronic databases due to the slight difference in inclusion/exclusion criteria (i.e., geographical restrictions on process evaluations).

#### 4.2.1 Updated Review of Quantitative Impact Evaluations

As the present review is an update of a previous review of school‐based interventions to reduce school exclusion (Valdebenito et al. 2018), studies included in this earlier report are also included in this update. Furthermore, Valdebenito et al. (2018) searched for studies up to 2015, and so searches undertaken for this update were restricted to evaluations published between 2015 and 2022. Studies were not excluded based on the language of publication or their publication status and availability.

We followed recommendations put forward by Kugley et al. (2017) in relation to updating reviews and so minor changes were made to the searches from the previous review. For example, Open Grey was not searched and removed from the proposed list of electronic databases in our protocol. This database is no longer available. As per these guidelines, we also included numerous searches to supplement searches of online databases and included both published and unpublished studies.

### 4.3 Search Strategy

Searches for quantitative impact evaluations were conducted by M.J. A.‐B., and searches for qualitative process evaluations were conducted by S.H., S.V., and H.G. supported the search process. The results of all searches were exported and uploaded to Covidence, a review management software, for screening. Bibliographic references were administered in Zotero.

#### 4.3.1 Search Terms and Keywords

All searches were conducted using a selected set of keywords, which were present and outlined in our protocol (Valdebenito et al. 2023). Keywords covered terms related to the type of study, type of interventions, population, and outcomes. Table 1 outlines the keywords. These keywords are also consistent with those used in the previous review (i.e., Valdebenito et al. 2018).

**Table 1:** Search terms used in electronic database searches.

Type of study	Interventions	Population	Outcomes
Evaluation Effectiveness Intervention Programme Programme effectiveness Impact Effect Experimental evaluation Quasi‐experimental evaluation RCT Random evaluation Efficacy trial Process evaluation Implementation Facilitators* Access* Barriers*	Disciplinary methods Token economy Classroom management programme/intervention/strategies School management Early interventions School support projects Skills training	Schoolchildren Pupils Children Adolescents School‐aged children Student Youth Adolescent Young people United Kingdom*	School exclusion School exclusion reduction Suspension reduction Out‐of‐school suspension In‐school suspension Out‐of‐school exclusion In‐school exclusion Suspended Suspension Expelled Expulsion Outdoor suspension Stand‐down Exclusionary discipline Discipline

* Keywords included only in searches for qualitative process evaluations.

Keywords were combined using Boolean operators (e.g., AND, OR, NOT), wildcards and truncation symbols, and combinations of key terms and search operators were created according to the specific database. Following recommendations for best practice, where possible, we adapted searches using subject headings or descriptors appropriately for each database (Kugley et al. 2017). For example, for education databases such as ERIC or Canadian Business & Current Affairs (CBCA), we use the thesaurus or subject index to assess the fit of our keywords for these specific databases. For each electronic database, pilot searches were conducted, and the results of these pilot searches were used to adjust the terms, synonyms, and wildcards. The pilot searches also assisted in the creation of combinations of keywords that captured relevant evaluations.

Appendix S1A outlines the search syntax for searches conducted on each electronic database for quantitative impact evaluations and qualitative process evaluations, along with the dates searches were undertaken, the total number of studies located, and the total number of studies retrieved.

#### 4.3.2 Electronic Searches

As per our protocol, a comprehensive number of electronic databases were searched to identify as many evaluations of school‐based interventions to reduce school exclusion as possible. We included databases for published literature, grey literature (e.g., ProQuest Dissertation and Theses Global), and literature in languages other than English (e.g., SciELO).

All databases that were searched are listed in Table 2. Some databases (e.g., Australian Education Index and CBCA) were not searched for qualitative process evaluations, given our geographical restrictions in the qualitative evidence synthesis.^4^

**Table 2:** Electronic database searched.

Database	Platform
Australian Education Index	ProQuest
British Education Index	EBSCO
BMJ Controlled Trials	
Canadian Business & Current Affairs^a^	ProQuest
ClinicalTrials.gov	
Criminal Justice Abstracts	EBSCO
Cochrane Central Register of Controlled Trials (CENTRAL)	
Database of Abstracts of Reviews of Effects (DARE)	
Educational Resources Information Centre (ERIC)	EBSCO
EThOS	
EMBASE	Ovid
Google Scholar	
Institute of Education Sciences – What Works Clearinghouse	
ISI Web of Science	Clarivate
MEDLINE	PubMed
ProQuest Dissertations & Theses Global	ProQuest
APA PsycInfo	EBSCO
Sociological Abstracts	ProQuest
Social Sciences Citation Index (SCCI)	ISI Web of Science
Scientific Electronic Library Online (SciELO)	ISI Web of Science
Science.gov	
World Health Organisation International Clinical Trial Registry Platform (WHO ICTRP)	

^a^ We are grateful to Patrick Labelle for facilitating searches of this database.

The selection of databases was informed by searches preformed for the previous review. However, some databases were no longer available and hence could not be searched for the present update. These databases were: Index to Theses database (ProQuest), The Netherlands National Trial Register (NTR), and Open Grey.

#### 4.3.3 Searching Other Resources

In addition to electronic databases, we undertook several additional searches to supplement the systematic search strategy. For example, we searched several institutional websites, hand searched relevant journals, screened and harvested references from relevant reviews, and contacted experts. We also rescreened studies previously excluded by Valdebenito et al. (2018) due to changes in the inclusion criteria for the current updated review (i.e., inclusion of QEDs).

These additional searches were conducted concurrently for quantitative impact evaluations and qualitative process evaluations. Multiple members of the research team were involved in the supplemental search process. The following sections outline these additional searches in more detail.

##### 4.3.3.1 Website Searches

Websites of several international and governmental organisations were searched for possibly includable evaluations. Organisations were those that have produced research evidence in the field of education and were selected based on the review team's prior knowledge and recommendations from experts.

Searches on websites used combinations of ‘school exclusion’, ‘school suspension’ and ‘school discipline’ terms. Websites were searched on 13th May 2023 by three members of the research team (D.J., M.J. A.‐B., and S.V.). Table 3 outlines the results of these searches. Two studies included in the quantitative review were identified using website searches, that is, Humphrey et al. (2018) and Kendziora et al. (2019).

**Table 3:** List of organisation websites searched.

Organisation	Website	Number of results	Number of results retained	Number of included studies
CAF Development Back of Latin America	http://www.caf.com/en/topics/e/education/	140	0	0
Civil Rights Project	http://www.civilrightsproject.ucla.edu/research	129	5	0
Education Endowment Fund	educationendowmentfoundation.org.uk/	38	6	1
Excluded Lives	excludedlives.education.ox.ac.uk/excluded-lives-project/	25	3	0
International Development Research Centre (IDRC)	www.idrc.ca/en/program/education-and-science	37	0	0
IPPR	http://www.ippr.org/	1420	0	0
US Department of Justice Office of Justice Programmes	www.ojp.gov	42	2	2
Together Transforming Behaviour	www.togethertransformingbehaviour.com/			
UNICEF	www.unicef.org/education	34	0	0
UNESCO	www.unesco.org/en/publications	96	3	0
World Bank	www.worldbank.org/en/research/brief/publications	27,472^a^	0	0

^a^ Only the first 10 pages (approximately 2000 results) were screened, as subsequent pages yielded increasingly less relevant material.

##### 4.3.3.2 Forward Citation Searches

Forward citation screening of evaluations included in the earlier review by Valdebenito et al. (2018) was also undertaken to supplement our systematic search process. These were conducted on Google Scholar between 25th and 27th April 2023 by one member of the team (H.G.). Each of the RCTs included in the previous review (*n* = 37) was located on Google Scholar, and the studies listed as citing each study were screened. Titles of all results were initially screened on Google Scholar, and any possibly includable results were retained. Google Translate was used for any non‐English titles.

Table 4 outlines the number of citations screened and the number of results retained for each study. Eight results were duplicates, and as such, a total of 228 results of citation searches were retained for further screening. Of these, 55 had been identified in our electronic database searches. As such, an additional 173 studies were screened.

**Table 4:** Citation searches results.

References	*N* citations	*N* results retained
Allen et al. (1997)	609	15
Arter (2005)	5	0
Barnes et al. (2003)	290	14
Berlanga (2004)	7	0
Bradshaw et al. (2012)	536	49
Bragdon (2010)	3	0
Brett (1993)	3	0
Burcham (2002)	5	0
Collier (2002)	8	0
Cook et al. (2014)	126	5
Cornell et al. (2012)	127	8
Crowder (2001)	4	0
Dynarski et al. (2003)	152	2
Edmunds et al. (2012)	135	10
Farrell et al. (2001)	321	16
Feindler et al. (1984)	368	2
Harding (2011)	5	0
Hawkins et al. (1988)	301	2
Hirschfield and Celinska (2011)	54	3
Hostetler and Fisher (1997)	42	0
Ialongo et al. (2001)	134	13
Johnson (1983)	1	0
Lewis et al. (2013)	119	6
Mack (2001)	2	0
Obsuth et al. (2017)	42	5
Okonofua et al. (2016)	382	29
Panayiotopoulos and Kerfoot (2004)	10	1
Peck (2006)	3	0
Reese et al. (1981)	10	0
Russell (2007)	3	0
Shetgiri et al. (2011)	29	3
Smith (2004)	18	0
Snyder et al. (2010)	206	22
Sprague et al. (2016)	2	0
Tilghman (1988)	4	0
Ward and Gersten (2013)	67	11
Wyman et al. (2010)	234	20
Total	4367	236

The majority of these studies were excluded using information reported in the study abstracts (*n* = 107). Of the 66 studies retained and screened based on the full‐text, 4 were included in the review (Barrett and Harris 2018; Glenn et al. 2021; Meadows 2018; Neace and Muñoz 2012).

##### 4.3.3.3 Targeted Hand‐Searches and Reference Harvesting

Some electronic databases were hand searched, specifically, the Campbell Collaboration Social, Psychological, Educational and Criminological Trials Register (C2‐SPECTR) and the National Dropout Prevention Centre Network. These hand searches were undertaken alongside systematic searches of electronic databases.

In addition, hand searches and reference harvesting of relevant reviews, books, policy briefs, and reports were undertaken by one member of the research team (M.J. A.‐B.). In total, 106 additional sources were screened through this process. These sources, listed in Appendix S1B, were initially identified during the title and abstract screening of search results from our systematic searches. Although many of these sources did not meet the review's inclusion criteria—particularly books and policy briefs—they were considered highly relevant to the field of school exclusion. To minimise the risk of missing eligible evaluations, the reference lists of all identified sources were screened. The majority were reviews (e.g., systematic reviews and meta‐analyses; *n* = 79), and any potentially includable studies cited within them were harvested and recorded in May 2023.

A number of journals related to our research questions were also hand‐searched for possibly includable evaluations in May 2023 by one member of the research team (S.H.). Issues published between 2015 and 2023 of the following journals were hand‐searched: *School Psychology Review, Journal of School Psychology*, and *Journal of Research on Educational Effectiveness*. We conducted hand searches of journals specialising in education and evaluation research where electronic access was unavailable or incomplete. Specifically, we manually examined the tables of contents and special issues of the five journals most frequently represented in our list of included studies. This process covered issues published from October 2015 onwards to ensure the identification of potentially eligible studies not captured through electronic database searches. While this was not a field‐wide hand‐search, we believe it enhanced the sensitivity of our search strategy in areas where database coverage may have been limited. Table 5 outlines these hand searches. In total, 1013 articles were screened using the title and abstract, and 38 articles were retained and screened using the full text. Full‐texts were either accessed on journal websites or located using Google Scholar. No unique studies eligible for inclusion were identified by hand searches.

**Table 5:** Hand searches of journals.

Journal name	Year	*N* screened using T&A	N screened using FT
School Psychology Review	2015	30	2
	2016	26	2
	2017	24	1
	2018	29	0
	2019	30	4
	2020	38	3
	2021	41	3
	2022	137	3
	2023	9	1
Journal of School Psychology	2015	10	0
	2016	38	3
	2017	44	3
	2018	62	3
	2019	60	2
	2020	35	2
	2021	50	0
	2022	66	5
	2023	29	1
Journal of Research of Educational Effectiveness	2015	6	0
	2016	41	1
	2017	37	0
	2018	27	0
	2019	30	0
	2020	34	0
	2021	33	0
	2022	32	0
	2023	14	0
Total		1013	38

Abbreviations: FT, full‐text; T&A, title and abstract.

##### 4.3.3.4 Rescreening of Previously Excluded Studies

As this review constitutes an update of Valdebenito et al. (2018), all studies included in the earlier version were retained. However, the current review expands the scope of eligible designs: while the 2018 review included only RCTs, this updated version also includes QEDs that employ a control group and pre/post‐test data, with statistical adjustment for baseline differences. This amendment to the eligibility criteria necessitated the rescreening of studies previously excluded solely on the basis that they were non‐randomised designs.

Due to resource constraints, the rescreening process focused only on studies excluded during the full‐text screening stage in the 2018 review. According to that review, 516 studies were excluded at the full‐text stage; of these, 514 could be retrieved and were therefore re‐assessed in the present update. Two members of the review team (H.G. and S.V.) undertook the rescreening process.

Rescreening was conducted in two stages. In the first stage, studies were screened at the title and abstract level to identify whether they (1) included a control or comparison group, and (2) reported outcomes related to school exclusion. All 514 records were screened by one reviewer (H.G.), and 33.6% (*n* = 98) were screened independently by a second reviewer (S.V.). No discrepancies were identified. A total of 292 studies were excluded at this stage.

In the second stage, full texts of the remaining 147 studies were screened against the revised eligibility criteria, using a standardised checklist for quantitative evaluations (Appendix S1C). Each study was assessed by one reviewer (H.G.), and 32% (*n* = 47) were independently double screened by S.V. Any disagreements were resolved through discussion, with consensus reached in all cases. Of these, 142 studies were excluded at this stage.

As a result, five additional studies were newly identified as eligible for inclusion (Flay and Allred 2003; Rentschler and Wright 1996; Robinson 1985; Schoeneberger 2008; Zivin et al. 2001). However, three of these (Robinson et al.) were subsequently excluded from the meta‐analysis due to insufficient statistical information for effect size computation.

##### 4.3.3.5 Contacting Experts

Our final supplementary search was to contact experts in this area. Table 6 provides a list of 25 experts who were contacted. These individuals were provided with information about the review, our research questions, and the inclusion/exclusion criteria. They were asked to share any possibly includable evaluations they may have undertaken or published, or any relevant research in this area. Experts were selected based on their recognised expertise and substantive contributions to the field of education and school exclusion. The selection process prioritised individuals with a strong track record of peer‐reviewed publications or influential policy work on school discipline, educational exclusion, and related interventions. Particular attention was given to ensuring a balance of methodological expertise, including both qualitative and quantitative researchers, as well as those with experience in mixed‐methods approaches. Experts were affiliated with leading academic institutions or held senior roles in government departments and non‐governmental organisations focused on education policy and practice. In addition, we sought to include professionals whose work addressed the disproportionate impact of exclusion on vulnerable groups, such as students with special educational needs, those from disadvantaged backgrounds, and minority ethnic communities. While most experts were based in the United Kingdom, international scholars from the United States were also included, given the global relevance and influence of their research in this area.

**Table 6:** List of experts contacted.

Name	Affiliation	Quantitative/qualitative
1. Dr. Kate Allen	University of Bristol	Qualitative
2. David Bartram OBE	Persistent Education	Qualitative
3. Tom Bennett OBE	Department for Education	Qualitative
4. Professor Catherine P. Bradshaw	University of Virginia	Quantitative
5. Dr. Lorraine Campbell	University of Sheffield	Qualitative
6. Tracey Campbell	Together Transforming Behaviour	Qualitative
7. Dr. Andrew Christie	Birmingham Children's Trust	Qualitative
8. Lauren Cross	University of Cambridge	Qualitative
9. Professor Harry Daniels	University of Oxford	Quantitative
10. Professor Feyisa Demie	Durham University	Qualitative
11. Dr. Tasmin Ford	University of Cambridge	Qualitative
12. Professor Tony Gallagher	Queen's University Belfast	Quantitative
13. Professor Patricia Gándara	University of California, Los Angeles	Quantitative
14. Kiran Gill	The Difference	Qualitative
15. Dr. Lorraine Hansford	University of Exeter	Qualitative
16. Dr. Helen Knowler	University College London	Qualitative
17. Daniel Losen	University of California, Los Angeles	Quantitative
18. Professor Sarah Martin‐Denham	University of Sunderland	Qualitative
19. Professor Gillean McCluskey	University of Edinburgh	Qualitative
20. Monika Mielke	University of Pennsylvania	Quantitative
21. Dr. Ingrid Obsuth	University of Edinburgh	Both
22. Professor Gary Orfield	University of California, Los Angeles	Quantitative
23. Dr. Jeffery Quaye	Department for Education	Qualitative
24. Jonathon Simmons	Varkey Foundation	Qualitative
25. Dr. Ian Thompson	University of Oxford	Qualitative

Experts were emailed in April 2023 by two members of the research team (S.H. and M.J. A.‐B.) and no unique studies were retrieved this way.

### 4.4 Data Collection and Analysis

#### 4.4.1 Selection of Studies

Screening of studies was conducted in Covidence. Initially, studies were screened based on the title and abstracts. M.J. A.‐B. screened the studies identified in searches for quantitative impact evaluations and S.H. screened studies identified in searches for qualitative process evaluations.

Studies retained after screening of titles and abstracts were then screened based on the full text. Full text screening was undertaken in duplicate at this stage, and all studies were screened by at least two members of the review team. The eligibility checklists in Appendix S1C were used to guide screening.

Screening of studies eligible for inclusion in our meta‐analysis of impact evaluations was undertaken by M.J. A.‐B., S.V., D.J., and H.G., who acted as independent second screeners for impact evaluations. The inter‐rater reliability ranged from (0.69) to (0.93), and the overall agreement ranged from (92.5%) to (99.3%). Disagreement between reviewers on whether to include or exclude a study was discussed and resolved.

S.H. and H.G. screened all full texts retained for our qualitative evidence synthesis of process evaluations independently. The inter‐rater reliability was (0.75), and the overall agreement was good (93.6%). The majority of conflicts occurred due to different reasons for excluding studies. Conflicts were resolved until a consensus was reached.

#### 4.4.2 Data Extraction and Management

All screening was undertaken in Covidence, but data extraction and management were performed in MS Excel. Using formulae previously described raw data to compute effect sizes was extracted from primary studies and recorded. Where possible, we computed difference in difference standardised mean differences (SMDs) as the chosen effect size for studies that reported outcomes as continuous variables. If no baseline data were reported, we computed endline only SMDs. If outcomes were reported dichotomously, we computed odds ratios and then transformed these to *d* (Appendix S1E).

In cases where results of primary evaluations were reported using statistical models (e.g., regression or ANOVAs) and no raw data was available, effect sizes were computed using test statistics reported. The formulae to compute effect sizes in this way are those that are used in the Campbell Collaboration effect size calculator.

Effect sizes for all outcomes, timepoints, and subgroups were extracted from all studies included in this review. We re‐extracted effect sizes from the RCTs included in the previous review. In total, 819 effect sizes were extracted. H.G. and S.V. extracted effect sizes independently of one another. Effect size extraction was not done in duplicate. However, H.G. extracted all effect sizes from the previous review, where S.V. had previously extracted effect sizes. As such, these effects were extracted in duplicate.

The data extraction tool outlined in Appendix S1D was used to code information relating to moderator variables. Data extraction of moderator variables was undertaken by S.V. and H.G. It was not undertaken in duplicate.

#### 4.4.3 Assessment of Risk of Bias in Included Studies

As per our protocol, assessment of risk of bias in included studies was conducted using the EPOC tool for RCTs (Cochrane Effective Practice and Organisation of Care [EPOC] 2007) and the ROBINS‐I tool (Sterne et al. 2016) for QEDs. RCTs were assessed using the EPOC tool to ensure consistency with the risk of bias assessment conducted in the earlier review (i.e., Valdebenito et al. 2018). The risk of bias assessment for quantitative impact evaluations was led by A.S. and carried out in duplicate with S.V. or D.J.

##### 4.4.3.1 Research Questions 7 and 8: Process Evaluations

We also assessed the quality of included process evaluations using the CASP checklist for qualitative studies. This process is outlined in Section 4.4.12.

#### 4.4.4 Measures of Treatment Effect

The effectiveness of school‐based interventions was assessed using effect sizes for treatment‐control comparisons reported by included studies. Studies reported the effectiveness of outcomes, measured either dichotomously (e.g., percentage of students excluded vs. percentage of students not excluded) or as a continuous measure (e.g., the mean number of exclusions in schools). Formulae used here can be found in Appendix S1E.

##### 4.4.4.1 Corrections and Transformations

The present review conducted meta‐analyses of multiple outcomes. As a result, the direction of effect sizes was not necessarily coded in the same way. For example, in some instances where a decrease in the outcome was a desirable intervention effect (e.g., school exclusions, violence, or offending) the formula for an odds ratio would result in an OR less than 1 indicating a positive impact of the intervention. However, in instances where an increase in the outcome is desirable, the formula would result in an OR greater than 1, indicating a positive impact of the intervention. Thus, we adjusted the direction of odds ratios for outcomes where a decrease is desirable. The natural logarithm scale (LnOR) was used for all computations using odds ratios.

Additionally, included evaluations may have reported the impact of an intervention on a rare event. For example, being permanently excluded from school or engaging in violent offending. In such instances, values used to compute an odds ratio may include zero (i.e., no students were excluded, or no participants reported violence). In these cases, we used an odds ratio continuity principle to compute an effect size. In these cases, 0.5 was added to each value in the 2 × 2 frequency table used to estimate the OR. The weighted overall effect size in our meta‐analysis was represented as Cohen's *d* or the SMD. Therefore, for the analysis, effect sizes estimated as ORs were transformed to Cohen's *d* before computation.

#### 4.4.5 Unit of Analysis Issues

For the purposes of this systematic review, we include primary studies involving pupils and clusters of pupils as units of analysis. One key issue emerges when meta‐analysis includes studies randomising clusters or units. Participants nested in the same cluster tend to share similarities (intra‐class correlation [ICC]). When this correlation is not accounted for, standard errors (SEs), confidence intervals and *p*‐values tend to be too small. These conditions affect the meta‐analysis in two different ways. First, the primary trial gets a mistakenly high weight. Second, the pooled result produces a meta‐analysis with an overly small SE. To avoid the combination of individual and clustered data, we plan to follow the strategy proposed by the Cochrane Handbook for Systematic Reviews of Interventions (Higgins and Green 2011). The handbook suggests that the effective sample size in a cluster‐randomised trial can be obtained by dividing the original sample size by the design effect, which equals 1 + (*M* − 1) × ICC. In this equation, *M* is the average cluster size and ICC is the intra‐cluster correlation coefficient.

#### 4.4.6 Dealing With Missing Data

Where statistical information required to compute an effect size was missing, we contacted primary and corresponding authors of papers to retrieve missing information. Where information about moderators was missing, we followed the same procedure.

#### 4.4.7 Assessment of Heterogeneity

We report weighted mean effect sizes with robust adjusted SEs. Heterogeneity between effect sizes is assessed by the *Q*‐value, degrees of freedom and the value of *I* ^2^. We include the *I* ^2^ as the value of *Q*, which can appear distorted if the number of studies included in a meta‐analysis is small (Higgins et al. 2003), and it can also be transformed easily to a percentage value.

We report the variance components for each level of a three‐level meta‐analytical model and conduct a sensitivity analysis to assess the suitability of the multilevel model.

#### 4.4.8 Assessment of Reporting Biases

In the present meta‐analysis, we assessed the risk of bias for RCTs and QEDs using two distinct evaluation tools tailored to each study type.

For the RCTs, we employed the EPOC tool, a recognised instrument for evaluating the methodological quality of healthcare interventions in systematic reviews. This tool allowed us to systematically assess various aspects of RCT quality, including randomisation, allocation concealment, blinding, and handling of incomplete outcome data.

To assess risk of bias in studies employing a QED design, we utilised the Risk of Bias in Non‐randomised Studies of Interventions (ROBINS‐I) tool. ROBINS‐I is specifically designed to evaluate the risk of bias in non‐randomised studies, including QEDs. This tool enabled us to thoroughly examine the quality and potential sources of bias in QEDs, considering factors such as confounding, selection bias, and measurement of outcomes.

By employing these tailored assessment tools for each study type, we ensured a comprehensive and context‐specific evaluation of the risk of bias, enabling us to draw more precise conclusions regarding the quality and reliability of the evidence within our study.

#### 4.4.9 Data Synthesis

To evaluate the impact of interventions aimed at reducing school exclusions, we conducted a meta‐analysis of effect sizes. This included both primary outcomes (e.g., school exclusion) and secondary outcomes (e.g., antisocial behaviour and substance use). As anticipated, the data presented both hierarchical (between‐study) and correlated (within‐study) structures of dependency across the extracted effect sizes. To appropriately model this complexity, we employed robust variance estimation (RVE), which allows for the inclusion of dependent effect sizes without violating assumptions of independence.

To guide the selection of the most suitable meta‐analytic model, we applied the decision‐making framework developed by Pustejovsky and Tipton (2022). This decision tree provided a structured and empirically supported approach to choosing among RVE models based on key characteristics of the data, including the number of studies (clusters), the presence and nature of within‐study dependence, the nesting structure of effect sizes, and the inclusion of moderator variables. Depending on these characteristics, the decision tree recommends whether to use a correlated effects model, a hierarchical model, or a model with small‐sample corrections. For example, in our case, the presence of multiple effect sizes per study, variation in outcome type, and a moderate number of studies led us to implement a three‐level random‐effects model with robust SEs using the CR2 correction.

All analyses were conducted using the *metafor* package in r‐studio (Viechtbauer 2010), with the coding script included in the technical appendices of the final report. This analytic strategy ensured that our modelling approach was both theoretically grounded and statistically appropriate for the data structure, increasing the robustness and replicability of the findings.

#### 4.4.10 Investigation of Heterogeneity: Meta Regression

The exploration of heterogeneity encompasses several key factors, including gender, ethnicity, grade at school, and the type of intervention. To explore sources of statistical heterogeneity, meta‐regression models were employed. Moderator analyses examined whether differences in intervention type, implementation context, participant characteristics, or study design contributed to variability in effect sizes. These analyses were critical in identifying conditions under which interventions were more or less effective.

#### 4.4.11 Sensitivity Analysis

In the context of our multilevel meta‐analysis, we conducted sensitivity analyses specifically tailored to address outliers and estimate correlations within the covariance matrix. These analyses were instrumental in assessing the stability and reliability of our results.

We systematically examined the influence of outliers on our pooled effect sizes, ensuring that our findings were not unduly affected by extreme data points. Additionally, we scrutinised the impact of estimated correlations within the covariance matrix, a critical aspect of multilevel meta‐analysis. By doing so, we enhanced the robustness and precision of our meta‐analytic outcomes, providing more confidence in the validity of our findings.

#### 4.4.12 Treatment of Qualitative Research

We assessed all included process evaluations for methodological quality. This was performed using the CASP checklist (Critical Appraisal Skills Programme 2024) for appraisal of qualitative research (see Table 7). This tool is designed to enable reviewers to consider three issues related to the quality of a qualitative process evaluation. Items refer to the validity of the results, the content of the results, and also the application of the results (CASP 2024), and responses include ‘yes’, ‘no’, or ‘can't tell’. Assessment of qualitative process evaluations was undertaken by two members of the research team independently (H.G. and D.J.), and all differences or disagreements were reconciled.

**Table 7:** Example CASP checklist used to assess quality of process evaluations.

Section	Question
Are the results valid? [Section A]	Was there a clear statement of the aims of the research?
	Is a qualitative methodology appropriate?
	Was the research design appropriate to address the aims of the research?
	Was the recruitment strategy appropriate to the aims of the research?
	Was the data collected in a way that addressed the research issue?
	Has the relationship between researcher and participants been adequately considered?
What are the results? [Section B]	Have ethical issues been taken into consideration?
	Was the data analysis sufficiently rigorous?
	Is there a clear statement of findings?
Will the results help locally? [Section C]	How valuable is the research?

#### 4.4.13 Data Extraction and Management

Data extraction from process evaluations was undertaken in MS Excel using an inductive thematic analytical approach. Two members of the research team (H.G. and S.H.) independently extracted data from included process evaluations in relation to the factors that facilitated or hindered the implementation of the intervention in schools. The themes and subthemes categorised by researchers were recorded, alongside supporting evidence and information about the source of the evidence (e.g., a quote from a teacher/student/school leadership, or an interpretation by the researcher, and the page number).

#### 4.4.14 Data Synthesis and Analysis

We adopted a thematic synthesis framework to synthesise findings from included process evaluations. Themes and subthemes that were extracted from individual process evaluations were recorded and then grouped into overarching streams to explore the factors that facilitated the implementation of interventions in a school‐based setting and those factors that were perceived as a barrier. Our results are discussed in relation to these facilitators and barriers.

### 4.5 Deviations From Protocol

Our protocol specified that we would only include process evaluations that reported findings on participant perspectives at the end of an intervention. However, while screening search results, we identified that this criterion was less important for qualitative research. As such, we adjusted our criteria and process evaluations that reported findings on participant perspectives either during their participation in an intervention or following completion of an intervention were eligible for inclusion. Qualitative research conducted with participants either before an intervention or in the absence of an intervention (e.g., to ascertain schoolteachers' perceptions of exclusion; Done and Knowler 2021) were excluded.

Similarly, our protocol outlined that we would include process evaluations that reported information on participants' perceptions of interventions to reduce school exclusion. However, in practice, this was very difficult, as given our geographical limits, there were no process evaluations of interventions that aimed to reduce exclusions or suspensions in UK schools and as such, we modified our inclusion criteria. Included process evaluations therefore needed to report findings from a process evaluation of an intervention that aimed to modify or target student behaviour. Moreover, we included process evaluations that, if they met other inclusion criteria, presented some findings relevant to student behaviour. This decision was made so as to provide some information about facilitators and barriers to the implementation of interventions in UK schools. However, it does impact the systematic nature of our searches for qualitative evidence, and as such, this is discussed in further detail as a possible limitation of our mixed methods review.

## 5 Results of the Quantitative Systematic Searches

### 5.1 Description of Studies

#### 5.1.1 Results of the Searches

**Figure 1:**
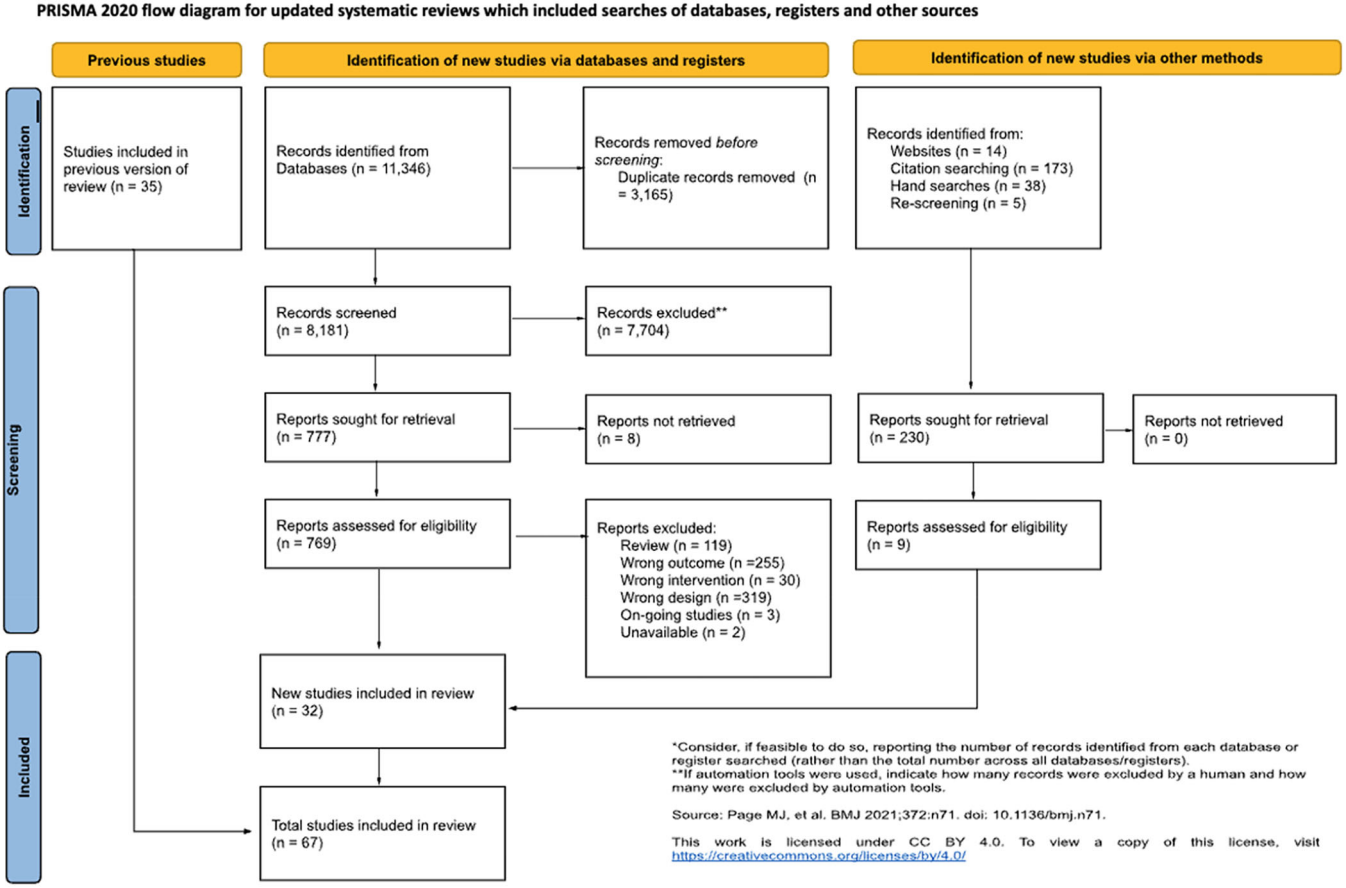
PRISMA flow diagram for quantitative studies. *Source:* Page et al. 2021. *BMJ* 372:n71. https://doi.org/10.1136/bmj.n71.

Systematic searches were undertaken to identify and retrieve a population of both published and unpublished studies that aligned with our inclusion criteria to assess the quantitative effectiveness of school‐based interventions to reduce school exclusion. The results of these searches are illustrated in the PRISMA flow diagram (i.e., Figure 1).

The implementation of the planned search strategy resulted in the identification of 11,346 articles sourced from 22 electronic databases. Consistent with our established methodology, we augmented the electronic searches using three supplementary strategies: (i) an examination of reference lists from manuscripts identified in systematic searches; (ii) contacting experts in the field; and (iii) a comprehensive review of backward citations. The all‐encompassing list of studies (*N* = 11,346), accompanied by their corresponding references, was imported into Zotero and Covidence. Covidence facilitated the management of pertinent bibliographic references, citations, screening processes, full‐text reviews, assessments of risk of bias, extraction of study characteristics and outcomes, as well as the subsequent exportation of data and references (Babineau 2014).

Following the elimination of duplicate entries, a total of 8181 manuscripts were retained for the screening phase. This screening process, overseen by three members of the research team (i.e., M.J. A.‐B., S.V., and H.G.), facilitated the evaluation of each manuscript based on the information presented in its title and accompanying abstract. Upon completion of the screening, a total of 7404 studies were excluded due to their lack of relevance to the objectives of the present review. Consequently, 777 manuscripts were retained for full‐text screening.

Eligibility was determined at this stage using the full text of each manuscript, where one was available. Our screening was guided by our inclusion criteria, with a primary focus on aspects related to study design, targeted population, and outcome measures and results. The eligibility stage was carried out independently by four members of the research team (i.e., M.J. A.‐B., D.J., H.G., and S.V.), utilising a screening tool (Appendix S1C) aligned with the inclusion/exclusion criteria detailed in Section 4. The assessment of eligibility concluded with 737 studies being excluded (refer to Section 5.2 for reasons for exclusion), while 8 studies were categorised as ‘studies awaiting classification’. These latter manuscripts met our inclusion criteria but did not report sufficient data for effect size calculations. Details of the studies awaiting classification are documented in Table 10.

In total, 32 evaluations provided a sufficient amount of statistical data, qualifying them for inclusion in the meta‐analysis. These studies were included alongside 35 studies previously included by Valdebenito et al. (2018) resulting in 67 studies included in our updated meta‐analysis.

### 5.2 Excluded Studies

*Inter‐Rater Reliability:* In line with our protocol, all 777 full texts were screened by two members of the research team. M.J. A.‐B. screened all 777 manuscripts, and H.G., D.J., and S.V. acted as the second reviewers. We utilised Cohen's Kappa to gauge inter‐rater reliability (Cohen 1960; Sim and Wright 2005). The Kappa value ranges from 0 (indicating agreement equivalent to chance) to 1 (indicating perfect agreement between raters). Our analysis produced a Cohen's Kappa of 0.84, signifying a substantial level of consensus between the evaluators. Upon completion of this agreement assessment, the evaluators collectively addressed any conflicts, and these were resolved through collaborative discussions. When a unanimous consensus proved elusive, guidance was sought from a senior team member. In alignment with our protocol, we excluded 737 manuscripts from further consideration. The reason for exclusion of each manuscript is elaborated upon in Table 8.

**Table 8:** Summary of the reasons for the exclusion of 737 papers.

Reason for exclusion	*N*	%
Outcome measure was absent	255	34
Type of intervention	30	4
Methodological design	319	43
Reviews	119	16
On‐going	3	0.00
Inaccessibility issue (unavailable)	11	1.5
Total	737	100

*Outcome:* Among the reports, 255 (34%) did not present a suitable measure of school suspension. In certain specific instances, the primary outcome was either unreported (e.g., Neluheni 2015; Ortega 2018) or reported within a composite measure along with other disciplinary actions that did not involve any form of school exclusion, for instance, office disciplinary referrals (e.g., Middendorf 2020; Stanton 2019). Due to the inability to isolate a distinct measure of school suspension in these cases, we opted to exclude these reports. In other exceptional situations, school exclusion was treated as a predictor rather than an outcome (e.g., Dean et al. 2019; McCray 2020).

*Type of Intervention:* Following our protocol, we excluded 30 (4%) studies because the tested intervention was not delivered in schools, supported by schools or with at least one component implemented in school settings. We also excluded evaluations of interventions delivered in alternative schools for high‐risk students (e.g., Adomako Letterlough 2018; Baroni et al. 2020; Stencler 2019) and those delivered in special education schools (e.g., Clark 2019; Johnson 2015).

*Methodological Design:* Three hundred and nineteen (44%) studies were excluded because they did not satisfy the methodological characteristics defined in the protocol. We excluded studies lacking a control group (e.g., Hunt 2015; Operton 2017; Ralston 2015) and those studies where the control group was not equivalent in demographics and risk factor variables to the intervention group (e.g., Jenson et al. 2018; Lantz 2017; Shoppe 2019). Unsuccessful matching procedures (e.g., Amaya 2020; Dohrmann 2020) were also excluded. As stated in the published protocol, qualitative studies not aligned with the pre‐defined inclusion criteria were excluded from the present review (e.g., Kappler 2019; Mann 2017).

*Reviews of Research:* During the searches, we kept 119 manuscripts which corresponded to literature reviews (e.g., O'Reilly et al. 2018; Raynor et al. 2015), systematic reviews (e.g., Bruhn and McDaniel 2021; Freeman 2018; Koh and Askell‐Williams 2021) or meta‐analysis (e.g., Mielke and Farrington 2021; Valdebenito et al. 2018) related to school suspension or behavioural problems in schools. These types of manuscripts were initially retained on the understanding that they could be a source for identifying extra primary research reports. All 119 of these studies were excluded in the second round of assessment once we had checked their citation lists.

*Inaccessibility Issue:* Despite our diligent attempts to reach out to authors and secure interlibrary loans, a total of 11 (1.5%) studies had to be excluded due to our inability to access the complete text of the reports.

#### 5.2.1 Studies Awaiting Classification

Eight studies remained unclassified due to insufficient statistical data for meta‐analysis calculations. In all the cases, the authors were contacted via email or social networks at least twice. A compilation of studies that could potentially be incorporated into a future iteration of this review is provided in Table 9.

**Table 9:** Studies awaiting classification.

References	Type of publication	Design	Sample	Outcomes	Intervention
Bergman and Chan (2017)	Working paper	RCT	1137	School suspensions	Text‐messaging technology
Bohnenkamp et al. (2021)	Journal	RCT, not enough data for ES calculation	40 schools	School suspension, Bullying, Juvenile Justice referrals	Emotional and Behavioural Health–Crisis Response and Prevention (EBH‐CRP)
Bradshaw et al. (2021)	Journal	RCT	58 schools	School Suspension	Multitiered system of supports for behaviour (MTSS‐B)
Cave (2021)	PhD thesis	QED	10 schools	School suspension	PBIS
Pas et al. (2019)	Journal	QED (PSM)	26,000 schools	School suspension, Truancy	SWPBIS
Rebok et al. (2019)	Journal	QED (PSM)	Unclear	School suspension, expulsion, ODR	Experience Corps (EC) Baltimore
Smokowski et al. (2020)	Journal	RCT	24 schools	School suspension, violent behaviour, Anxiety, bullying victimisation	School Based Teens Courts (SBTC)
Villavicencio et al. (2018)	Report	QED	Unclear	School Suspension	Unclear

### 5.3 Included Studies

#### 5.3.1 Characteristics of Included Impact Evaluations

This meta‐analysis included 32 new studies. The subsequent sections of this report summarise the characteristics of these studies, including their methodological features, participant characteristics, and the intervention specifics.

*Characteristics of the Included Studies:* As per our protocol, we included studies reporting interventions that were carried out and reported between 2015 and 2022. Exceptionally, this review involves one manuscript published in 2023 (Huang et al. 2023). The study was identified via the electronic searches, and for this reason, we decided to include its results in the present study. Also, we included five studies that were a product of the re‐screening exercise (Ballard et al. 2014; Brady et al. 2007; Fisher and Robinson 1994; Neace and Muñoz 2012; Rentschler and Wright 1996). Therefore, the date of publication of the included papers ranged from 1994 to 2023 (*M*_date_ = 2016; SD = 6.4). As indicated by the data presented in Table 10, we have incorporated both published and unpublished reports in a balanced distribution. Each of these reports was composed in English and predominantly represents studies conducted in the United States (94%). Only one study (3%) included a sample originating from the United Kingdom. Despite our comprehensive search through global databases and international organisation websites, with a specific focus on databases from non‐European countries, our efforts did not yield studies conducted in alternative geographical locations.

**Table 10:** Study characteristics searches 2016–2022 (*n* = 32).

Study characteristics	Mean	SD
Publication year	2016.5	6.47
Range of years	1994	2023
Type of publication	*n*	%
Published peer reviewed journal articles	15	44
Unpublished literature (PhD thesis, Master thesis, Technical/governmental reports)	17	56
Language		
English	32	100
Country of the sample		
United States	30	94
United Kingdom	1	3
Unclear	1	3
Design		
QED (Propensity Score Matching)	8	25
QED	7	22
RCT	17	53
Evaluator role		
Independent	22	72
Design	2	6
Design and delivery	2	6
Unclear/Not reported	6	16
Unit of randomisation (Only RCT)		
Individuals	5	31
Clusters	12	69
Statistical analysis		
Multilevel modelling	11	34
Differences in means (MANOVA, *χ* ^2^, ANOVA, ANCOVA)	12	38
Regression	7	22
Other	2	6
Size of the sample		
< 300	4	13
Between 300 and 1000	7	22
> 1000	14	44
Unknown	7	22

The scope of the quantitative review focused on both experimental and quasi‐experimental study designs. Overall, 53% of evaluations included in the meta‐analysis were labelled experimental or RCTs and 47% of evaluations included were considered QEDs.

In the case of experimental studies or RCTs, participants were randomly assigned to one or more experimental groups (who received the intervention) and at least one control group. These control groups encompassed options such as a group with no intervention, a group receiving intervention as usual, a group receiving an alternative intervention, or a wait‐list control group. While 31% of the included studies randomised individuals, 69% randomised clusters of students, that is, entire schools or classrooms within schools. Where necessary, we corrected data in clusters to combine them with individual‐level data.

The included QEDs compared the effects of the independent variable by incorporating at least one treatment group and one control group. 25% of the studies tested statistical equivalence, employing techniques such as Propensity Score Matching.

In terms of analytical models, a majority of the studies compare means (38%) of the treatment and control groups. That was the case, specially for quasi‐experimental studies. In the case of RCTs, 11 out of 17 employed robust SE calculations, such as hierarchical regressions or multi‐level modelling.

#### 5.3.2 Characteristics of Participants in Included Studies

In the current meta‐analysis, most of the students were enroled in higher school grades, particularly middle and high schools (see Table 11). Additionally, the included data showed that these students were enroled in schools characterised by relatively high proportions of students identified as Black (34%), and a smaller proportion of those of White (16%). In fact, within the studies incorporated, four were conducted in schools where a minimum of 80% of the pupils were of Black (Barrett and Harris 2018; Glenn et al. 2021; MacIver et al. 2017; Meadows 2018). However, it is important to note that a considerable proportion of the studies did not provide metrics related to ethnicity (47%).

**Table 11:** Characteristics of participants.

Participants characteristics	*n*	%
Grade		
Elementary school	6	19
Elementary and Middle	4	13
Middle school	7	22
Middle and High	6	19
High	4	13
Whole school (K12)	2	6
Unknown	3	9
Ethnicity		
Predominantly Black or Afro‐American	11	34
Predominantly Latino	1	3
Predominantly White	5	16
Unknown	15	47
Free school meals	Mean	SD
	67	18.1

Within this report the percentage of students eligible for free school meals was used as an indicator of low socio‐economic status or population vulnerability (Hobbs and Vignoles 2010). Across our included studies, pupils who received free school meals were prevalent (*M*_fsm_ = 67%; SD = 18.1%).

#### 5.3.3 The Characteristics of Interventions

A wide range of school‐based interventions were included in the quantitative review. These included, for example, counselling, mentoring, community services, classroom management, after‐school academic support, and school‐wide strategies. Notably, 28% of these interventions targeted changes at the school, teacher, or parent level, while 63% aimed to impact pupils' skills and behaviours to influence suspension rates (Table 12).

**Table 12:** Characteristics of the interventions.

Intervention characteristics	*n*	%
Targeted change		
At school level	9	28
At student level	20	63
Other (parents, teachers)	3	9
Programme delivery		
External facilitators	8	25
School facilitators	11	34
School facilitators plus external facilitators	9	28
Unknown	4	13
Role of the evaluator		
Deliver the programme	0	0
Design the programme	2	6
Delivery and design	2	6
Independent	23	72
Unclear	5	16

As shown in Table 13, school facilitators (34%) or school facilitators assisted by externals (28%) delivered most of the interventions. This means that schools interested in reducing exclusion typically put resources into the intervention process.

**Table 13:** Types of intervention programmes.

Programme	Number of studies	% of studies
Risk reduction	5	16
After‐school programmes	1	3
Enhancing academic skills	1	3
Mentoring/monitoring	2	6
Skills training for students	6	19
Skills training for teachers	2	6
School‐wide strategies	6	19
Restorative Justice	3	9
Mental health	3	9
Skill training for parents	1	3
Violence reduction	2	6
Total	32	100

We coded data concerning the role of the evaluator. We identified four potential approaches undertaken by evaluators: (i) administering the programme, signifying that the researcher not only implemented the intervention but also served as the evaluator; (ii) designing the programme, indicating that the evaluator formulated or participated in formulating the theoretical foundation, objectives, and activities of the intervention (Frank et al. 2017; Meadows 2018); (iii) both designing and delivering the programme (Flynn et al. 2016); and (iv) independent evaluation, relating to those researchers or research teams where no research member was involved in any stage of the design or implementation (e.g., Humphrey et al. 2018; Siennick et al. 2020; Weist et al. 2022). As per our review, most of the interventions were evaluated by independent researchers (72%).

Table 13 shows the types of interventions that were used to address school exclusions either as a primary or secondary outcome. While this categorisation might seem constrictive in certain instances (due to the involvement of multiple components within some interventions), our aim was to formulate a comprehensive roster of categories that matched with the review published by Valdebenito et al. (2018).

*Risk Reduction:* We found five (16%) studies (i.e., Brady et al. 2007; Faria et al. 2017; Rentschler and Wright 1996; Sepanik et al. 2021; Siennick et al. 2020) that implemented interventions aimed at preventing risks such as antisocial behaviour/crime, drug use, and school dropout. Brady et al. (2007) and Siennick et al. (2020) examined the impact of school‐based interventions involving the police and the criminal justice system, targeting children with high‐risk profiles and low school attendance rates. Generally, these interventions, aimed at risk reduction, were influenced by the Risk‐Needs‐Responsivity model (Bonta and Andrews 2010). Faria et al. (2017) and Sepanik et al. (2021) were more focused on the prevention of students leaving school before graduation. Those interventions also identified risks and attempted to provide support and resources to the targeted students.

In general, these interventions utilised a multisystem team to assess youths and provide tailored services or referrals. The interventions emphasised customisation and coordination through team‐driven planning, offering various services such as tutoring, mediation, and substance abuse evaluations.

*After‐School Programmes:* One study provided data on interventions that offered students after‐school activities. The Oregon Mathematics, Engineering, Science Achievement (Greenberg Motamedi and Singh 2016) directed its afterschool initiatives toward middle and high school students and centred on the creation of inventions tackling critical issues in developing nations, including sustainable lighting, water transportation, water filtration, and prosthetics. In pursuit of this objective, MESA has established four curricular modules that encourage students to collaborate in conceptualising, constructing, assessing, and showcasing a functional solution tailored to a specific client.

*Enhancing Academic Skills:* We found one study targeting the enhancement of academic skills as a strategy to improve academic performance, increase motivation and promote more adaptive behaviour. Martinez (2012) tested an intervention to boost the academic progress of students and improve they behaviour at school.

*Mentoring and Monitoring:* These types of initiatives were evaluated in two studies (MacIver et al. 2017; Zoblotsky et al. 2020). These programmes established structured and nurturing connections between a young individual encountering academic, emotional, or behavioural challenges and a non‐parental adult mentor. The mentor, often a community volunteer, offers role modelling and prolonged support (MacIver et al. 2017). Alternatively, mentors served as student tutors, guiding academic performance, providing guidance, and aiding with scholastic tasks (Zoblotsky et al. 2020).

*Skills Training for Students:* We found six studies reporting the impact of skills training for school children. Some of these programmes were based on social learning and cognitive behavioural theories (Fisher and Robinson 1994; Gonzalez et al. 2020; Humphrey et al. 2018), and their goal was to enhance individuals' socio‐cognitive, socio‐emotional, and behavioural skills to regulate maladaptive behaviour. Social skills training programmes typically consist of a curriculum with focused training modules. Group‐based sessions and occasionally one‐to‐one sessions offer the opportunity to implement specific techniques (e.g., instruction, modelling, role‐playing, feedback and reinforcement) in a real‐world environment. Some more specific programmes target communication skills or approaches to reducing stress (Frank et al. 2017; Meadows 2018). One additional study reported the results of a skill training programme called Self Affirmation (Borman et al. 2022). The intervention aimed to enhance academic outcomes for students who might feel threatened by racial stereotypes. Primarily, the intervention targeted the reduction of the racial disparities in the use of exclusionary discipline. This approach encouraged Black students to concentrate on and affirm positive facets of their identities, such as excelling as siblings, children, athletes, and artists, among others. These changes are expected to improve discipline at school among minority students affected by the discipline gap.

*Skills Training for Teachers:* Two evaluations targeted teachers' skills. Manzeske et al. (2017) examined an intervention targeting the enhancement of teachers' academic proficiency in mathematics and reading. The study evaluated its effect on the academic achievements and behavioural aspects (attendance and disciplinary referrals) of students in primary school. Flynn et al. (2016) reported the impact of a professional development model designed to empower teachers with skills and resources to create positive classroom environments benefiting a wide range of students. The intervention aimed to assist teachers in perceiving behaviour as a form of communication and creating supportive environments that mitigate misbehaviours through addressing needs and imparting skills to students. Workshops and coaching sessions centred on transitioning teachers from their punitive responses to fostering inclusivity. Taking a slightly different approach.

*School‐Wide Interventions:* We identified six evaluations that evaluated the impact of school‐wide interventions on students' behaviour. The programmes were characterised by holistic approaches encompassing systemic modifications throughout the entire school. These interventions encompass students, educators, parents, and occasionally the community within which the school operates. Three of the studies focused on testing PBIS in elementary and middle schools (Barrett and Harris 2018; Gage et al. 2019; Gage and Stevens 2018). These initiatives strive to establish a positive atmosphere through well‐defined regulations that encourage positive conduct, learning, and safety. School‐wide interventions possess the potential to address both institutional and individual needs of schoolchildren. An additional three studies assessed interventions with the goal of enhancing safety and reducing negative outcomes such as aggression or violence. Others aimed to ease the difficult transition between middle and high school. These programmes employed data‐driven approaches to determine appropriate student support. For instance, they provided alternative approaches to managing problematic behaviour, address mental and behavioural health challenges, and promote prosocial behaviour by targeting influential malleable risk and protective factors (Bos et al. 2022; Corrin et al. 2016; Kendziora et al. 2019).

*Restorative Justice:* Restorative justice was used by three studies (Augustine et al. 2018; Glenn et al. 2021; Huang et al. 2023) included in the present review. Restorative practices (RP) are an alternative to exclusionary disciplinary approaches. These emphasise the resolution of misconduct through addressing relational harm and promoting the reparation of relationships.

*Mental Health Interventions:* We included three studies which primarily focused on the provision of mental health services. Thompson et al. (2021) reported findings on the effects of a programme known as the Family Access Centre of Excellence (FACE). FACE utilises the Family Check‐Up method to assess family strengths and challenges within a child‐focused, family systems framework. A treatment strategy is then devised, which establishes connections to appropriate services and employs intensive case management to address obstacles directly. Another approach, the Interconnected Systems Framework (ISF), addresses the common limitations of many school‐wide programmes by providing specific guidance on systematically connecting mental health clinicians and schools (Weist et al. 2022). ISF involves expanding school team membership to include administrators and active PBIS and SMH experts. This approach broadens data systems to include universal mental health screening, which is then used to implement evidence‐based programming to all tiers. ISF fosters cross‐system partnerships, benefiting education by enhanced programming depth and mental health by improving connections to students and families.

*Skills Training for Parent:* One evaluation examined the impact of skills training for parents (i.e., Mason et al. 2016). The standard version of the Comprehensive Parenting Programme (CSP) entailed six 2‐h sessions, encompassing instruction, skill practice, video examples, and reviews. CSP Plus augments this structure by adding two sessions for adolescents and their parents—before and after the standard sessions. These additional CSP Plus sessions mirror the original CSP format but introduce content addressing high school transition goals, family communication, and decision‐making. The focus centred on adolescents' involvement in their learning journey.

*Violence Reduction:* We included two studies measuring the impact of violence reduction programmes (Dymnicki et al. 2021; Neace and Muñoz 2012). Although these interventions could be classified as skills training, we have isolated them because they are specifically targeted at increasing self‐control and safety and reducing violence.

### 5.4 Risk of Bias (RoB) in Included Studies

As with the previous review, we assessed risk of bias using the Cochrane Collaboration EPOC 2 risk of bias tool (Higgins et al. 2024). For QEDs, we used ROBINS‐I (Sterne et al. 2016). Both tools are intended to allow for an assessment of the risk of bias arising from different domains within research projects. From June 2023 until September 2023, we began the RoB assessment for all RCT (*n* = 17) and QED studies (*n* = 15) using these tools.

Overall, and as is often reported, risk of bias was lower for experimental than quasi‐experimental studies and there was much greater variation in risk of bias within quasi‐experimental studies than in randomised trials. For example, there was only one experimental study considered to be high risk of bias in 7/8 domains, whereas with quasi‐experimental studies there were seven that were serious, or critical in terms of RoB.

#### 5.4.1 RoB in Experimental Studies Using EPOC

The EPOC instrument proposes the following eight criteria for the assessment of quality bias:

1. Sequence generation.
2. Allocation concealment.
3. Baseline outcome equivalence.
4. Baseline characteristics equivalence.
5. Incomplete outcome data.
6. Blinding of outcome assessment.
7. Protection against contamination.
8. Selective outcome reporting.

Each of these domains was judged on a 3‐point scale (i.e., low risk, high risk, unclear risk). The EPOC tool provides guidance and examples for each domain that facilitate the decision of assigning low, high or unclear risk. Three members of the team performed the assessment of risk of bias (S.V., D.J., and A.S.). Assessment of bias was performed independently.

**Figure 2:**
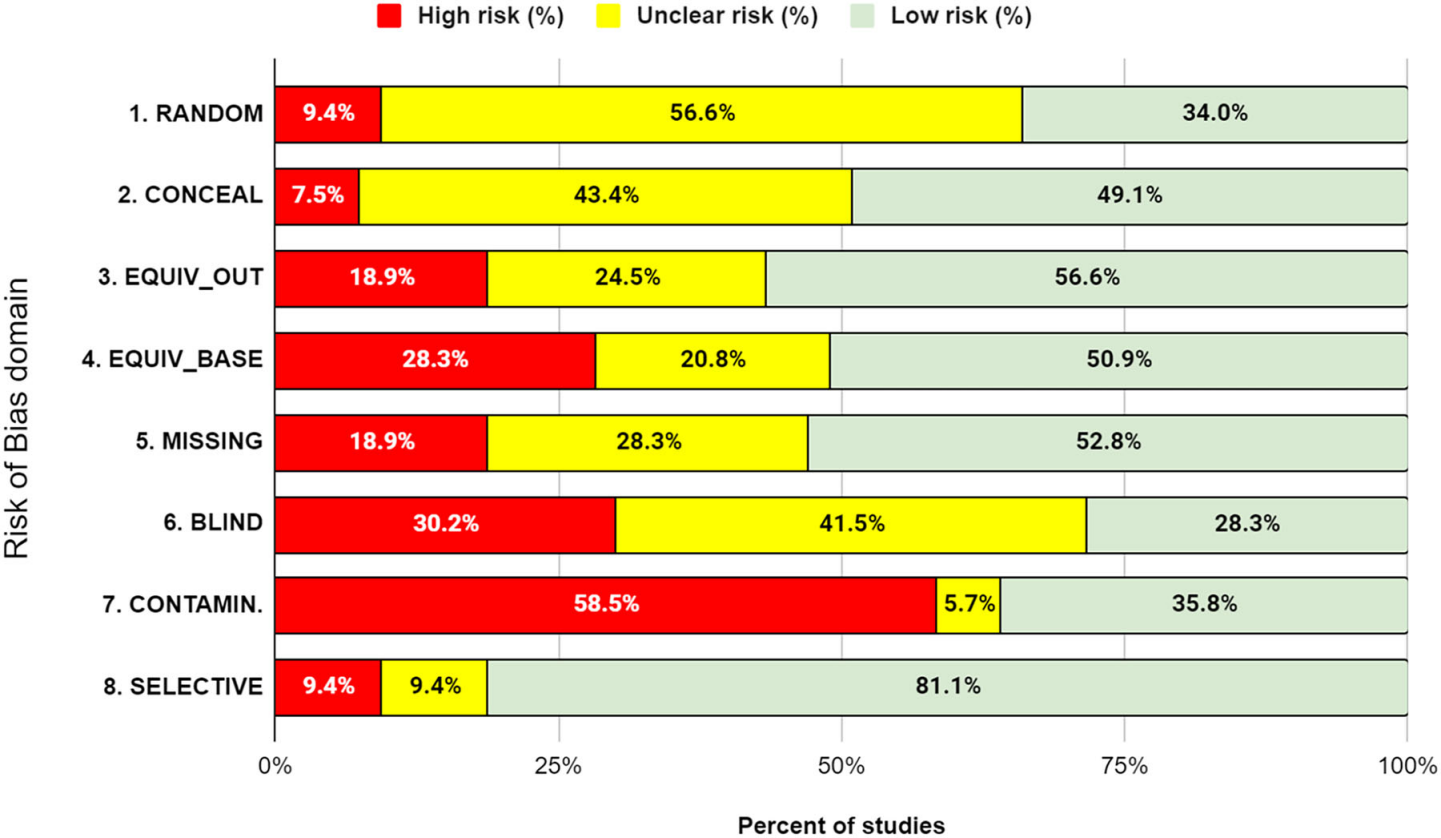
EPOC risk of bias randomised controlled trials.

The methodological quality of each publication included in the review was evaluated using the EPOC risk of bias tool. The instrument helps reviewers to evaluate the internal validity of reported results across several dimensions. Three coders (S.V., D.J., and A.S.) independently applied the EPOC tool to each study at different locations and did not confer on ratings until after all papers were assessed. Below, we report the results for each of the eight criteria involved in EPOC. Figure 2 shows a summary of the overall result.

Focusing on Figure 2, we can see the variation between RoB domains for the experimental studies, with criteria ‘7. Contamination’ carrying the greatest proportion of ‘high risk’ overall (58.5% of assessed studies). That said, in six of eight domains, more than 50% of studies were assessed as either ‘high’ or ‘unclear’ risk of bias. Recalling that assessments are driven by what is written in outputs, this does not mean there is bias, but without clear reporting, we cannot assume low risk of bias. For domain seven, contamination, this is driven by the use of individually randomised trials that randomise pupils or units such as classrooms within the same schools. The nature of some interventions may mean that the risk of contamination—where controls receive treatment—is lower. But where units are randomised within schools, it means that treatment and control pupils are in the same school, typically in the same year, and may even be in the same class.

*Adequate Sequence Generation:* Adequate sequence generation relates to whether a study team used an appropriate procedure to randomise groups in an experimental design, that is, was allocation actually random? Appropriate methods used to generate the allocation are, for instance, the use of random number tables or a computer random number generator (Higgins et al. 2024). Regardless of the approach taken, it should be described in enough detail to allow an assessment of whether the sequence generation was adequate.

Five studies (9%) presented a high risk of bias with respect to allocation sequence (Figure 2). Mason et al. (2016) used a sequential allocation sequence after stratifying by school and pupil gender. Sequential allocation itself is perfectly predictable, which is one source of concern as predictability opens itself to subversion, but Mason et al. also report that allocation factored in the order with which families returned permission slips (p. 4). It is not clear whether, at any point, putative participants were randomly sorted before the sequential allocation sequence was used. As such, this approach represents a high risk of bias. Another paper assessed as having a high risk of bias from the previous review was Allen et al. (1997). In that study, the authors describe using three different methods of randomisation (i.e., picking names from a hat, coin toss and using an alphabetical list of names) as well as running randomisation at different levels in the same study (i.e., individuals and classrooms). This mixture of methods could lead to a high risk of bias in the results: in particular, the use of a list of names is known not to be appropriate for allocating cases.

Under the categorisation of ‘unclear risk’, more than half of the studies (56.6%) were assessed as unclear for sequence generation. This is because many reports presented descriptions of randomisation or having randomised without detailing the methods of sequence generation, or, for example, if software was used (e.g., Arter 2005; Brett 1993; Reese et al. 1981; Meadows 2018).

Finally, nearly one‐third (32.7%) of manuscripts were defined as presenting a low risk of bias (e.g., Hirsch et al. 2019; Augustine et al. 2018). For example, one study explained that randomisation was stratified by pupil sex and year group, and that stratified randomisation was conducted using Stata following the specified procedure.

*Allocation Concealment:* Correct allocation concealment safeguards a rigorous implementation of the randomisation process by not allowing researchers or participants prior knowledge of the results of assignment (i.e., by using sealed envelopes or another procedure that prevents knowledge about the group that the participant is going to be allocated to). This is to prevent subversion of allocation on the basis of preference or choice based on knowledge of what the next patient/unit will ‘get’. To achieve this, Schulz and Grimes (2002) suggest that allocation should be centralised and executed at the beginning of the study, but can also be achieved with ‘trickle trials’ where a centralised allocation system is used to randomise patients as they enter the trial (Ariel et al. 2021).

In this meta‐analysis, 7% of studies were evaluated as having a high risk of bias for allocation concealment. Some examples of high‐risk studies are those where reports suggested that schoolteachers instead of researchers performed the random allocation (e.g., Crowder 2001), or where randomisation appears to have failed (e.g., Kendziora et al. 2019).

We also found a high percentage of studies (44%) reporting minimal details of allocation concealment. Those studies were classified as ‘unclear risk’ of bias. More positively, nearly half of the remaining studies were classified as having a low risk of bias, typified by randomisation being done at the start of the study, with all units randomised at once. For example, Humphrey et al. (2018, 7) describe the allocation process as having been conducted:

…independently of the authors by the Clinical Trials Unit at the Manchester Academic Health Science Centre and was balanced by proportions of children eligible for free school meals (FSMs) and speaking English as an additional language (EAL) via adaptive stratification (minimisation).

So, a team independent of the study authors carried out the randomisation, and we know from the study that the units randomised were schools. Schools were also stratified according to pupil deprivation and EAL, and randomisation was via minimisation.

*Baseline Equivalence in the Outcome Measured:* Pre‐existing baseline differences between groups—particularly of outcomes—could suggest problems with randomisation, hence, it is a key focus when assessing risk of bias (Shadish et al. 2002). Having assessed this for the RCTs, the majority (nearly 60%) were classified as ‘low risk of bias’—meaning that before randomisation treatment and control were equivalent in terms of exclusion and/or suspension. A further one‐quarter of studies (24.5%) were classified as ‘unclear risk’—for example, Dymnicki et al. (2021) discuss balance but present no evidence of this in their paper, meaning it was not possible to assess this.

Finally, we found that nearly 20% of studies had a high risk of bias, meaning that before randomisation, treatment and control groups were not equivalent on pre‐intervention outcomes (e.g., suspension or exclusion). This matters because an imbalance at baseline that is not appropriately adjusted for in analysis might then drive a later ‘result’ (i.e., introduce bias). One example of a study that did not report such an adjustment was Panayiotopoulos and Kerfoot (2004). Another example relates to the imbalance of a specific type of exclusion in Berlanga (2004) and Kendziora et al. (2019), where in‐school exclusion was not balanced before randomisation.

Note that for assessment of balance in outcome or baseline characteristics we did not focus on whether there were statistically significant differences between groups, but focused on means and distributions (Altman 1985).

*Baseline Equivalence in Other Participants' Characteristics:* We found a higher proportion of imbalance on participant characteristics, leading to around 30% of studies being assessed as high risk of bias. Again, imbalance at baseline does not invalidate a study or prevent valid inference (Senn 2013), but not adjusting for a pre‐existing difference may reduce power. That said, systematic differences between group characteristics—as with Kendziora et al. (2019)—that is not consistent with chance variation suggests a failure of randomisation (Higgins et al. 2024). A further 22% did not report enough data for judgement (i.e., unclear risk), for example, Kendziora et al. (2019), and 50% of studies were assessed as presenting a low risk of bias (e.g., Faria et al. 2017).

*Addressing Incomplete Outcome Data:* Incomplete outcome data, whether through attrition or being unable to collect outcome data, is an obvious issue for any intervention study. The fact that many studies pursued more than one measure across time makes it likely that attrition or other forms of missingness affected sample sizes.

As per the previous review, we assess a high risk of bias when (i) substantial attrition was present in the study and the researchers did not mention a strategy to deal with that issue (e.g., Peck 2006); (ii) when they used list‐wise deletion (e.g., Russell 2007), and (iii) in those cases where the attrition affected the treatment or control group in an unequal proportion of missing cases across arms (e.g., Hostetler and Fisher 1997). Using these criteria, we found that 20% of the studies presented a high risk of bias. A further 30% did not report enough data to be judged. The remaining 50% were evaluated as presenting a low risk of bias. Low risk cases involved (i) those studies reporting zero attrition; (ii) studies where attrition was represented by a low percentage of cases; (iii) when missingness was equivalent in the treatment and the control group, and (iv) when the researcher reported attrition, analysed it and used appropriate methods to deal with attrition (e.g., multiple imputation, full information maximum likelihood).

*Blinding of Outcome Assessment:* This criterion covers bias arising in situations where those collecting outcome data are aware of the condition assigned to each participant (e.g., individuals, classrooms, schools). In the majority of studies, administrative data from school official records were used to collect outcome data, which carries a lower risk of bias than self‐reports of exclusion. But the use of administrative data means that blinding to allocation is likely infeasible for treatment pupils. This is because pupils being excluded or suspended will go through a process before being sent away from school. (One can imagine being on a new scheme might be seen as mitigation in that process before exclusion. Similarly, we can speculate that blinding to allocation might be possible for control pupils if those disciplining a pupil were not informed/did not know they were part of a study.) Overall, knowledge of allocation would not, in our view, be a source of bias, precisely because of the process around exclusion and suspension. In short, manipulating administrative records to bias them for or against a particular intervention—which would raise serious questions about the probity of all involved—seems implausible.

Having said that, 30% of the studies coded were assessed as a high risk of bias. We assigned a high risk of bias when those in charge of delivering the intervention were also collecting data or when teachers rated the students' behaviour while being aware of the allocated condition (e.g., Tilghman 1988). In any case, it must be said that in school‐based experiments, blinding is likely infeasible. Most of the studies require at least a minimal participation of school staff, and the allocated condition is mostly evident for participants (Hutchison and Styles 2010). (But see the discussion of this point below relating to ROBINS‐I—in brief, it is unlikely that suspension and exclusion would be biased even with knowledge of treatment allocation.).

As in previous cases, the number of reports lacking the data to evaluate the blindness of the outcome assessment (i.e., unclear risk) was high (~40%).

*Protection Against Contamination:* Contamination means that control groups receive treatment, either directly through accessing resources, or via interaction with treated units. Schools present a challenging environment when it comes to contamination, by virtue of the way schools are organised or want to implement (e.g., by class or whole school). As a result, it is very difficult to keep separate treatment and control pupils, even if schools are willing to try (and speaking from experience, they often are not). Study design is a key means to protect against contamination. In the case of educational research, cluster randomised designs are viewed as the best way to prevent the mixing of treatment and control pupils, as well as aligning with school preferences for implementation (Connolly et al. 2018). As such, any school study implemented via a cluster‐randomised design inherently lowers the risk of bias via contamination and was rated as such in our assessment. However, as Figure 2 illustrates, contamination risk was very high in assessed studies, with nearly 60% being ‘high risk’, and a further ~6% being unclear risk. This is almost entirely due to the use of individual randomisation, with pupils as the unit of randomisation, meaning that schools in the trials hosted both treatment and control pupils. That said, it is mainly studies from the previous review that this problem relates to (e.g., Cook et al. 2014; Smith 2004); of the new trials assessed in the updated review, nearly two‐thirds were cluster‐randomised trials (e.g., Obsuth et al. 2017; Humphrey et al. 2018).

Three studies did not report enough data for evaluation and were categorised as unclear risk of bias (e.g., Mason et al. 2016). Finally, 36% of the studies were classified as low risk of bias. Normally low risk studies were cluster‐randomised experiments where control and treatment participants were in different schools (e.g., Corrin et al. 2016; Snyder et al. 2010).

*Selective Outcome Reporting:* Selective outcome reporting occurs when there is a difference between the proposed outcomes for evaluation and those finally reported. In our evaluation, five studies (10%) presented a high risk of selective outcome reporting (e.g., Panayiotopoulos and Kerfoot 2004; Russell 2007). In the case of thesis or trials without published protocols, the assessment was only based on discrepancies between outcomes proposed and reported in those documents, but we acknowledge that not having a protocol should likely default to studies being classed as ‘high risk’. Approximately 80% of studies were assessed as low risk of bias, and 10% did not report enough data for judgement so were ‘unclear’.

### 5.5 RoB Using ROBINS‐I

ROBINS‐I asks reviewers to assess studies across several domains. These cover confounding, bias in analyses, missing data, and various forms of selection bias. Within each section, there are initial signalling questions that, if answered in certain ways, prompt further questions. This process means that while ROBINS‐I seems initially unwieldy because of the number of questions, it is possible to progress through it relatively quickly (further methodological comments on ROBINS‐I provided below).

1. Confounding: Baseline confounding occurs when one or more prognostic variables (factors that predict the outcome of interest) also predicts the intervention received at baseline.
2. Selection of Participants Into Study: When exclusion of some eligible participants, or the initial follow‐up time of some participants, or some outcome events, is related to both intervention and outcome, there will be an association between interventions and outcome even if the effect of interest is truly null. This type of bias is distinct from confounding.
3. Classification of Interventions: Bias introduced by either differential or non‐differential misclassification of intervention status. Non‐differential misclassification is unrelated to the outcome and will usually bias the estimated effect of the intervention towards the null. Differential misclassification occurs when misclassification of intervention status is related to the outcome or the risk of the outcome.
4. Deviations Bias that arises when there are systematic differences between experimental intervention and comparator groups in the care provided, which represent a deviation from the intended intervention(s).
5. Missing Data: Bias that arises when later follow‐up is missing for individuals initially included and followed (e.g., differential loss to follow‐up that is affected by prognostic factors); bias due to exclusion of individuals with missing information about intervention status or other variables such as confounders.
6. Measurement Outcomes: Bias introduced by either differential or non‐differential errors in measurement of outcome data. Such bias can arise when outcome assessors are aware of intervention status, if different methods are used to assess outcomes in different intervention groups, or if measurement errors are related to intervention status or effects.
7. Selection of the Reported Result: Selective reporting of results from among multiple measurements of the outcome, analyses or subgroups in a way that depends on the findings.

**Figure 3:**
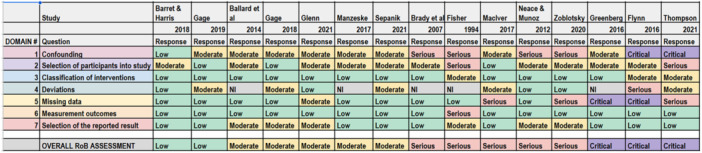
Overview ROBIN‐I.

Figure 3 below shows the overall summary of risk of bias across the 15 papers assessed by domain and then the overall assessment. Studies are organised in ascending order of overall risk of bias from Low to Critical, and then alphabetically therein. Each domain can be scored as ‘low’, ‘moderate’, ‘serious’, ‘critical’ or ‘no information’.

As an overarching comment on how much we can ‘believe’ the results from these studies is that: the more rigorous the designs were, the lower the risk of bias assessment was. That does not hold all the time, but the two studies with the lowest risk of bias used a matched difference‐in‐differences design, and three of the four ‘critical’ risk of bias studies used weaker comparison designs (regression adjustment, case‐control, unmatched controls). That said, as is well known from decades of research, study rigour can help minimise but cannot remove the risk of bias. As with the experimental studies, there is variation between studies in terms of risk of bias, up to and including bias that fundamentally undermines the results from studies. The two areas where risk of bias was greatest in terms of severity were ‘confounding’ and ‘missing data’. The bias in terms of confounding is primarily driven by study design, and missing data comes primarily from attrition/lack of outcome data for one or more groups in the study. This also means that there is not always a simple answer for the quality of evidence from these types of studies—that needs assessment on a case‐by‐case basis—but as a starting point, the study design is a sensible heuristic for risk of bias.

In the next paragraphs, we work through these domains in turn and discuss examples of assessed studies under each heading.

*Confounding:* This domain can be boiled down to whether there is something about the design that means the result might be an artefact of something else that was happening at the same time, or driven by important predictors of outcomes (whether measured or not) determining the treatment group participants belong to.

As is apparent, there were studies that were not affected by confounding, and others that were critically undermined by it. From the ‘low’ RoB studies (Barrett and Harris 2018; Gage et al. 2019), this primarily comes from the use of matching and difference‐in‐differences. Matching provides some face validity to the idea in a target trial of baseline equivalence. Difference‐in‐differences incorporates both differences in changes over time and differences between units as part of the estimation approach, allowing the use of repeated measurement to help reduce unobserved heterogeneity (Cunningham 2021). Combining the two is a design that provides plausible estimates of causal effects because units are close on observables pre‐intervention, and divergences post‐intervention are assumed to be the result of the intervention.

At the other extreme, those scored as ‘serious’ or ‘critical’ employed designs that were often confounded by existing baseline differences (e.g., Brady et al. 2007; Neace and Muñoz 2012; Thompson et al. 2021), where the design and analysis could not account for such differences (e.g., Flynn et al. 2016), or where the design rested on the use of unmatched comparison groups taken from different schools which were then analysed as though randomly assigned (Fisher and Robinson 1994).

*Selection of Participants Into Study:* Many of the studies in this review were scored as ‘low’ risk of bias. This is because, in many cases, the intervention has simply been implemented, and the evaluation is post hoc, with administrative data used to compare treatment and control groups. This means that selection processes have already happened, typically on the basis of eligibility criteria or a selection process that precludes ‘gaming’ to be added once intervention has started, although it obviously does not preclude selection out of the study. Similarly, it also means that follow‐up periods for treatment and control groups coincide.

The exception in this domain being for sub‐question 5 that asked, ‘Were adjustment techniques used that are likely to correct for the presence of selection biases?’. Across the majority of studies, this was assessed as ‘No’, on the basis that even with good face validity for baseline equivalence, little consideration was given to selection bias that was unmeasured or unmeasurable. The canonical example of ‘often unmeasured’ is motivation to change, which is partly captured by the offer of treatment being refused. As such, we know that those who have refused treatment are likely to differ from those who accept it in both observed and unobserved ways, making it unwise to compare the two. Unfortunately, one study (Thompson et al. 2021) takes precisely this approach, creating their comparison group from ‘those referred but who did not engage’ (p. 31).

*Classification of Interventions:* As discussed under domain 2, eligibility and intervention groups were typically well defined at the start of the intervention, meaning that many studies were ‘low’ risk of bias. For example, for school‐wide intervention studies, such as Sepanik et al. (2021), the whole school is either taking part or not. In Sepanik et al. (2021), schools decided whether or not to use the early indicator and intervention systems (EIIS), so it is clear which schools are treatment versus control. For pupil‐level interventions such as Greenberg Motamedi and Singh (2016) it was also clear which pupils began treatment and who did not.

*Deviations:* Domain 4 asks what was delivered/received in an intervention and did that vary from what was planned. In the context of a target trial this is subdivided into treatment compliance (e.g., did those in treatment get any treatment?) and implementation fidelity (those that did get treatment, did it adhere to what was supposed to happen?), but ROBINS‐I does not distinguish in the same way.

In the initial signal question fidelity was not covered in detail in five studies (‘no information’), and some sub‐questions relating to co‐interventions are again missing in many studies—except for Zoblotsky et al. (2020), who exclude schools with similar interventions from their study. This domain also prompts about whether deviations are unbalanced between groups, which presupposes that there is knowledge of what happens in both groups. Studies in the review were largely silent on this, but in the context of a trial, we both care and make efforts to find out what BAU is because this will help tell the treatment effect ‘story’, particularly if a null result.

For other areas of this domain, specifically what would be ‘treatment fidelity’ in a trial, Gage and Stevens (2018) actually assess treatment impact by fidelity. Similarly, Zoblotsky et al. (2020) conduct a detailed fidelity assessment, so it is clearer whether the intervention was delivered as intended. For other studies, there were few attempts at assessing the impact of compliance, for example, as equivalent to compiler‐average‐causal‐effects (CACE) in a trial.

*Missing Data:* Missing data is a domain that can quickly allow for an assessment of the plausibility of results. That is, as with an experiment, with too much missingness overall or imbalanced missingness between groups, no matter how strong the overall design is, means we should be sceptical of the results. More than half of papers—nine in total—were assessed as low risk in this domain overall (Barrett and Harris 2018; Ballard et al. 2014; Brady et al. 2007; Fisher and Robinson 1994; Gage and Stevens 2018; Gage et al. 2019; Manzeske et al. 2017; Neace and Muñoz 2012; Sepanik et al. 2021). For one study, Manzeske et al. (2017)—there was missingness on outcomes, but only for a small percentage of participants.

For other studies, for example, Barrett and Harris (2018), the use of administrative data meant that there was minimal or no missing data, so this was rated as low risk. In other studies, such as Greenberg Motamedi and Singh (2016), large proportions of missingness critically undermine the results. With Greenberg Motamedi and Singh (2016) there was 31% missingness for some outcomes for the MESA students, with those students excluded from analyses (p. 31). This was also the situation for Flynn et al. (2016), who reported ‘50% of cases had missing data, and all outcome and predictor variables had some missing data’. Flynn et al. (2016) go on to describe approaches taken to handle missingness, such as imputation, which ROBINS‐I, which can be used to assess studies. However, from the perspective of a target trial, it would be very hard to convincingly argue that the extent of missingness and the bias resulting from it can adequately be dealt with. This is particularly the case when combined with assessing baseline data in this study—Table 14 in Flynn et al. (2016) clearly indicates that the treatment and control schools are not equivalent.

**Table 14:** Sixty‐seven included studies in the updated version.

Author	Type of publication	Name of the programme	Target of the intervention	Grades at school	Outcome measured	Research design	Sample	Cluster	Analysis	Country of the sample	Evaluator role
Allen et al. (1997)	Journal article	Teen Outreach	Student	9–12 grade	Suspension	RCT	695	No	Regression	US	Unclear
Augustine et al. (2018)	Technical report	Pursuing Equitable and Restorative Communities (PERC)	Student	1–12 grade	Out of school suspension	RCT	8940	Yes	Regression (robust SE)	US	Independent
Arter (2005)	Journal article	Positive Alternative Learning Support (PALS)	Student	Secondary school	General suspension	RCT	52	No	ANOVA	US	Deliverer
Ballard et al. (2014)	Journal article	Expanded School Mental Health Model (ESMH)	Student	K5–K8	Suspension	QED	307	No	ANOVA	US	Independent
Barrett and Harris (2018)	Technical report	Positive Behavioural Interventions and Support (PBIS)	School	Elementary and middle schools	Suspension Expulsion	QED (Propensity Score Matching)	101,868	Yes	PSM, DiD	US	Independent
Borman et al. (2022)	Journal	Impact Schools Initiative	Student	Middle and high school	Suspension	QED	20 schools	Yes	Means	US	Independent
Bradshaw et al. (2012)	Journal	School‐Wide Positive Behavioural Interventions and Support (SWPBIS)	School	Elementary	Suspension	RCT	12,334	Yes	MLM	US	Independent
Berlanga (2004)	PhD Thesis	Grades, Attendance and Behaviour (GAB)	Student	8th grade	ISS, OSS, Expulsion	RCT	80	No	MANOVA	US	Independent
Bos et al. (2022)	Journal	Self‐affirmation	Student	7th grade	Suspension	RCT	2149	Yes	Hierarchical regression	US	Independent
	Technical report	Building Assets and Reducing Risks (BARR)	School	9th grade	Suspension	RCT	21,529 students; 512 teachers	Yes	Linear regression with corrected errors	US	Independent
Bragdon (2010)	PhD Thesis	Teach Team Project	Student	8th grade	General suspension	RCT	68	No	ANOVA	US	Independent
Brett (1993)	PhD Thesis	Efficacy, DC	Student	7th grade	General suspension	RCT	126	Yes	Means	US	Independent
Burcham (2002)	PhD Thesis	Social problem solving skills training	Student	Middle school	ISS, OSS	RCT	71	No	ANOVA, ANCOVA	US	Deliver
Collier (2002)	PhD Thesis	Pro‐social skills training	Student	Elementary school	General suspension	RCT	60	No	ANCOVA	US	Deliver
Cook et al. (2014)	Technical report	BAM (skills‐training) and MATCH (tutoring)	Student	High school	OSS	RCT	106	No	Means	US	Independent
Corrin et al. (2016)	Technical report	The Diplomas Now Model	School	Middle and High schools	Suspension, Expulsion	RCT	14,950	yes	MLM	US	Independent
Cornell et al. (2012)	Journal article	Threat assessment	School	1–12 grade	General suspension	RCT	201	yes	ANOVA	US	Design
Crowder (2001)	PhD Thesis	Gang Resistance, Education and Training (GREAT)	Student	7th grade	ISS, OSS	RCT	109	No	*t*‐test	US	Independent
Dymnicki et al. (2021)	Journal	Safe Communities Safe Schools (SCSS)	School	6th, 7th and 8th grade	ISS‐OSS	RCT	62,590	Yes	MLM	US	Independent
Dynarski et al. (2003, 2004)	Technical report	21st Century Community Learning	Student	Elementary school	General suspension	RCT	968	No	Percentages	US	Independent
Edmunds et al. (2012)	Journal article	Early College High School Academic skills enhancing	Student	High school	OSS	RCT	1607	No	Multivariate linear regression	US	Independent
Faria et al. (2017)	Technical report	Early Warning Intervention and Monitoring System (EWIMS)	School	9th grade	Suspension	RCT	35,558	Yes	MLM	US	Independent
Farrell et al. (2001)	Journal article	Responding in Peaceful and Positive Ways (RIPP)	Student	6th grade	ISS, OSS	RCT	626	Yes	GEE	US	Design
Feindler et al. (1984)	Journal article	Anger control training		High school	General suspension	RCT	36	No	ANCOVA	US	Unknown
Fisher and Robinson (1994)	PhD thesis	Cradock At‐Risk Prevention Project (CAPP)	Student	Middle school	General suspension	QED	74	No	ANOVA	US	Independent
Flynn et al. (2016)	Journal	Ramapo Training	Teachers	1–12 grade	Suspension	QED	1675 schools	Yes	Hierarchical regression	US	Design and delivery
Frank et al. (2017)	Journal	Transformative Life Skills	Student	6th and 9th grade	Suspension	RCT	159	No	ANCOVA	US	Design
Gage et al. (2019)	Journal	School Wide Positive Behavioural Interventions and Support (SWPBIS)	School	Elementary, secondary and high school	ISS, OSS, Expulsion	QED (Propensity Score Matching)	1186 schools	Yes	ZIP regression and PSM	US	Independent
Gage and Stevens (2018)	Journal	School Wide Positive Behavioural Interventions and Support (SWPBIS)	School	Elementary	ISS, OSS	QED (Propensity Score Matching)	1051 schools	No	Poison regression	US	Independent
Glenn et al. (2021)	Technical report	Restorative Circles (RC)	Student	NA	Suspension	QED (Propensity Score Matching)	19,569	No	DiD	US	Unclear
Gonzalez et al. (2020)	Technical report	Tools for Life (TFL)	Student	Elementary and middle schools	Suspension	RCT	2740	Yes	MLM	US	Independent
Greenberg Motamedi and Singh (2016)	Technical report	MESA after school programme	Student	Middle and high schools	Suspension	QED (PSM)	89	Yes	Regression robust SE	US	Unclear
Harding (2011)	PhD Thesis	Over to you	Student	8th grade		RCT	48	No	Means	US	Unknown
Hawkins et al. (1988)	Journal article	Proactive Classroom Management	TeacherStudent	7th grade	Suspension, expulsion	RCT	160	No	ANCOVA	US	Independent
Hirsch et al. (2019)	Technical report	After School Matters	Student	High school	General suspension	RCT	535	No	MLM	US	Independent
Hostetler and Fisher (1997)	Journal article	Project CARE (Skill for parents and children)	Student	Third grade	General suspension	RCT	317	No	MANOVA	US	Unknown
Huang et al. (2023)	Journal	Restorative Practices	Student	K12	OSS	RCT	5878	Yes	MLM	US	Independent
Humphrey et al. (2018)	Technical report	Promoting Alternative THinking Strategies (PATHS)	Student	Elementary school	Suspension	RCT	5218	Yes	Hierarchical linear model	UK	Independent
Ialongo et al. (2001)	Journal article	Two interventions i. Classroom‐centred (CC) ii. Family‐school partnership (FSP)	Student, teacher	Elementary school	General suspension	RCT	678	Yes	Regression	US	Design
Johnson (1983)	PhD Thesis	ATTEND (Counselling and monitoring)	Student	Seventh and eighth grade	General suspension	RCT	60	No	*t*‐test, chi‐squared	US	Design and delivery
Kendziora et al. (2019)	Technical report	Safe Public Spaces Programme (SPS)	School	Middle School	Suspension	RCT	24 schools	Yes	Regression robust error	US	Independent
Lewis et al. (2013)	Journal	Positive Action Programme	School	K12	Suspension	RCT	14	Yes	MLM	US	
MacIver et al. (2017)	Journal	Mentoring	Student	Middle and high schools	Suspension	QED (Propensity Score Matching)	5 school districts	No	*t*‐test	US	Independent
Mack (2001)	PhD Thesis	ICAN Kids! Behavioural group counselling		Fourth to sixth grade	OSS	RCT	20	No	ANOVA	US	Design and delivery
Manzeske et al. (2017)	Technical report	National Board Certified Teachers on Students Achievements and Behavioural Outcomes	Teacher	Elementary school	ISS, OSS	QED (Propensity Score Matching)	70,862	No	Regression	US	Independent
Martinez (2012)	PhD thesis	Literacy intervention programme	Student	High school	Suspension	RCT	621	No	MANOVA	US	Unclear
Mason et al. (2016)	Journal	Common Sense Parenting and Common Sense Parenting+	Parents	8th grade	Suspension	RCT	321 parents	No	Multivariate path analysis	US	Independent
Meadows (2018)	Master Thesis	Mindfulness Intervention	Students	Elementary and Middle schools	ISS, OSS	RCT	130	No	Mann–Whitney *U*‐test	US	Design
Neace and Muñoz (2012)	Journal	Second Step	Student	K1	Suspension	QED	2047	No	Means	US	Unclear
Obsuth et al. (2017)	Journal	Engage in Education – London	Students	Middle schools	Suspension	RCT	738	Yes	MLM	UK	Independent
Okonofua et al. (2016)	Journal article	Empathic Discipline	Teacher	Middle school	General suspension	RCT	1682	Yes	Mixed effect linear regression	US	Independent
Panayiotopoulos and Kerfoot (2004)	Journal article	Home and School Support Project (HASSP)	Student	Primary school	ISS, OSS	RCT	124	No	Means	UK	Unknown
Peck (2006)	PhD Thesis	Student Targeted with Opportunities for Prevention (STOP)	Student	Fourth to eighth grade	Suspension, expulsion	RCT	1050	No	Chi‐squared	US	Independent
Reese et al. (1981)	Journal article	Preparation through Responsive Education Programmes (PREP)	Student	Seventh to ninth grade	General suspension	RCT	98	No	ANCOVA	US	Unknown
Rentschler and Wright (1996)	PhD Thesis	Project CARE	Student	Elementary school	Suspension	RCT	128	No	ANOVA	US	Independent
Russell (2007)	PhD Thesis	Coping Power (Skills training for reducing aggression)	Student	6th grade	General suspension	RCT	61	No	ANOVA	US	Independent
Siennick et al. (2020)	Journal	Multi‐Tier System of Support (MTSS)	Student	Unknown	ISS, OSS	RCT	869	No	Cox regression	US	Independent
Sepanik et al. (2021)	Technical report	Effects of Early Indicator and Intervention Systems in Oregon	Student	9th to 12th	Suspension	QED	65 Districts	No	Means	US	Unclear
Shetgiri et al. (2011)	Journal article	Violence and drug use reduction	Student	9th grade	General suspension	RCT	108	No	Means	US	Independent
Smith (2004)	PhD Thesis	The Personal Responsibility Group (Emotional Intelligence skills)	Student	High school	ISS, OSS	RCT	40	No	MANOVA	US	Deliver
Snyder et al. (2010)	Journal article	Positive Action	School	Elementary school children	General suspension	RCT	544	Yes	*t*‐test	US	Unknown
Sprague et al. (2016)	Unpublished paper	School‐Wide Positive Behavioural Interventions and Support (SWPBIS)	School	Middle school children	ISS, OSS, Expulsion	RCT	13,498	Yes	MLM	US	Independent
Thompson et al. (2021)	Journal	Face Days	Student	Unknown	ISS. OSS	QED	417	No	*t*‐test, chi‐squared	US	Independent
Tilghman (1988)	PhD Thesis	Counsellor Peers	Student	K7–8	General suspension	RCT	100	No	*t*‐test	US	Design and delivery
Weist et al. (2022)	Journal	Interconnected Systems Framework (ISF) for School Mental Health (SMH) and Positive Behavioural Interventions and Support (PBIS)	School	Elementary schools	ISS, OSS	RCT	24 schools	Yes	MLM	US	Independent
Wyman et al. (2010)	Journal article	Rochester Resilience Programme	Student	Elementary school	OSS	RCT	226	Yes	MLM	US	Design
Zoblotsky et al. (2020)	Technical report	Get the Picture!	Student	9th grade	Suspension	QED (Propensity Score Matching)	438	Yes	MLM	US	Design and delivery

*Measurement Outcomes:* We assessed all but one study (Fisher and Robinson 1994) as ‘low’ in this domain. However, this domain raises issues for how to assess the risk of bias in studies using administrative data for measuring outcomes. It is unlikely that schoolteachers and headteachers would not know that a pupil was part of a treatment in some way, either at school or at the individual pupil level. At face value, this might be viewed as problematic because this might lead to bias; concretely, a desire to shape an outcome on the basis of knowledge of treatment participation. However, exclusion and suspension are process‐heavy and are often the punishment of last resort. In English schools, for example, headteachers are accountable for exclusions and suspensions, and in the case of permanent exclusion, there are financial costs to schools associated with it (because state funding for the pupil goes with them if they are excluded). Overall, the level of collusion required to ‘game’ suspension and exclusion as outcomes, even unblinded, is implausible, particularly given the level of scrutiny and (legal) challenge those decisions can carry with them, let alone the reputational risks for schools if they were to be manipulated. As such, unblinded assessment of those outcomes, in our view, carries a low risk of bias, particularly when compared to other forms of data collection (self‐report or teacher‐report of exclusion). For Fisher and Robinson (1994), the RoB was rated as serious because those delivering treatment also measured outcomes following treatment for the intervention and comparison groups (see pp. 96–97), which further compounds the risk of bias due to the confounding in that study.

Likewise, there is little variation between studies in terms of the comparability of outcome assessment in terms of how outcomes were assessed because all studies used administrative data for suspension and exclusion. (Noting that we have not assessed any self‐report measures.).

*Selection of the Reported Result:* Nine studies were rated as low for this domain, with five as moderate. The final domain seems to relate to multiple testing—which it does insofar as prompt questions are asking about multiple testing. However, the real focus of this section is on whether there were multiple tests undertaken then only some reported. Given the direction that a ‘target trial’ should be the starting point for ROBINS‐I, this question makes sense—undertaking many tests then only reporting the results one wants is a serious risk of bias. We admit to some difficulty in this domain for QEDs. This is on the basis that exclusion and suspension are arguably part of the same family of outcomes, so the first signal question would prompt other questions being asked. However, from the studies reviewed, we had a few concerns with studies selectively reporting outcomes. This is not because there was nothing to be worried about, but because the reference point for ROBINS‐I, as with EPOC, is whether the outcomes planned to be reported in the paper were reported in the paper. This is because in many papers, the only reference point for what is planned to be reported is the paper's abstract or the text of the paper itself, rather than a protocol or any sort of pre‐specification. So, one would expect high levels of reported consistency at that point. As an example of one paper, we rated as ‘moderate’ risk, Fisher and Robinson (1994) undertake analysis via MANOVA but only report results for one comparison group (or the two in the study) versus the intervention group.

## 6 Results of the Meta‐Analysis

In the present chapter, we report the result of the updated meta‐analysis. These chapter involves studies reported in 2018 (Valdebenito et al. 2018) plus the updated searches for RCTs and QEDs (See Table 14).

### 6.1 Primary Outcome: Overall Impact

The primary aim of this mixed‐methods systematic review was to thoroughly analyse and assess the body of evidence related to the impact of various intervention strategies carried out within educational settings to minimise the rates of disciplinary exclusions. The review's comprehensive approach involved evaluating a wide spectrum of school‐based initiatives to determine their relative efficacy in achieving this objective.

In alignment with our protocol (Valdebenito et al. 2023), all meta‐analyses were run under a multilevel, random‐effects model with robust variance estimation (Pustejovsky and Tipton 2022). This method allows for the inclusion of dependent effect sizes in a meta‐analysis, for example, effect sizes that represent the effectiveness of multiple treatment arms in comparison to one comparison group (Moeyaert et al. 2017).

The calculations for the impact of interventions on school exclusion included 276 effect sizes from 67 studies, all of which provided sufficient statistical information for a meta‐analysis. These 67 studies constitute the updated review, combining the findings from the 2018 study by Valdebenito et al. (*n* = 35) with a more recent search for evidence (*n* = 32). All in all, the evidence is based on data spanning from 1980 to 2022. These studies represent a total sample of 394,242^5^ students who took part in an RCT or quasi‐experimental evaluation of a school‐based intervention to reduce school exclusion.

Overall, school suspension was slightly reduced in the experimental group compared with the control group, after treatment. Under a multilevel (three levels), random‐effects model with robust variance estimation (Pustejovsky and Tipton 2022), the standardised mean reduction was small but statistically significant SMD = 0.106; (95% CI: 0.039 to 0.173; *p* < 0.001).

In our analysis, we sought to determine whether nesting individual effect sizes within studies improved the overall model performance. To do so, we implemented a model where the level 3 variance, which represents between‐study heterogeneity, was constrained to zero. Essentially, this approach mirrors a simple random‐effects model assumption where all effect sizes are treated as independent, despite knowing they are not. Our findings revealed that the full (three‐level) model, compared to the traditional two‐level meta‐analysis, demonstrated a superior fit. This conclusion was supported by lower values of the Akaike (AIC) and Bayesian Information Criterion (BIC) for the full model, indicating more favourable model performance overall. We found that the three‐level model provided a significantly better fit compared to a two‐level model with level 3 heterogeneity constrained to zero (*X*_1_^2^ = 102.15; *p* < 0.001).^6^

**Figure 4:**
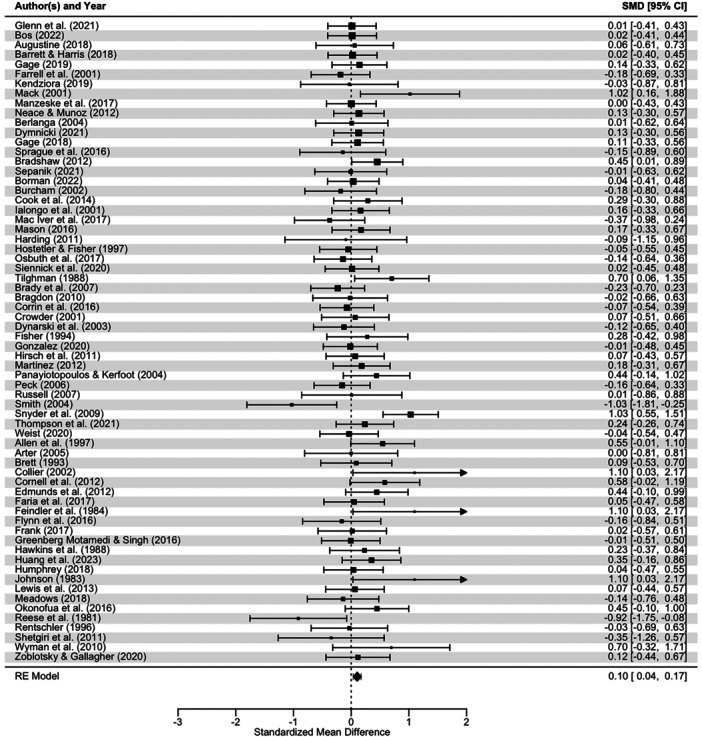
Multilevel meta‐analysis results. Overall impact of school‐based interventions on school exclusion.

Overall, the effect was small, positive and statistically highly significant, meaning that those participating in school‐based interventions were less likely to be suspended than those in the control group. To simplify the presentation of the findings, Figure 4 offers an aggregated view of the results, considering the full data set of 67 studies and 276 effect sizes.

Results exhibit significant heterogeneity (*Q* = 3505.13; *df* = 275; *p* < 0.001), which was expected in this meta‐analysis. We included different school‐based programmes, administering different ‘doses of treatment’ with participants from different locations and in different school grades, and so, variation between programmes was expected. The estimated variance components were *τ* ^2^_Level3_ = 0.044 and *τ* ^2^_Level2_ = 0.021. This means that *I* ^2^_Level3_ = 84% of the total variation can be attributed to between‐cluster (i.e., between studies) and *I* ^2^_Level2_ = 16% to within‐cluster heterogeneity (i.e., within studies).

To assess the expected range of effects in future applications of the intervention, we computed a 95% prediction interval based on the CHE (correlated and hierarchical effects) model with robust variance estimation. The prediction interval ranged from −0.402 to 0.614, suggesting substantial heterogeneity in the observed effects across studies. This means that the true effect in a new study, similar in context and design to those included in the meta‐analysis, could plausibly lie anywhere within this range—from small negative to moderate positive effects.

Importantly, the interval includes zero, indicating that while the average effect was positive, future studies could observe *no effect or even negative impacts*. This wide interval reflects both the *between‐study variance* and uncertainty in the model estimates, and it reinforces the need for caution when generalising the intervention's effectiveness across diverse settings. It also highlights the importance of considering *moderator variables* and contextual factors when interpreting the impact of these interventions.

### 6.2 Meta‐Analysis by Type of Disciplinary Suspension

We ran separate meta‐analyses for the different outcome measures found during the coding phase, that is, general suspension, in‐school suspension, out‐of‐school suspension and permanent exclusion (expulsion). Results of the four independent meta‐analyses are summarised below in Table 15. The findings suggest that the impact of included interventions on general suspension and in‐school suspension is similar to the overall effect size and also statistically significant. In the case of the most severe forms of exclusion, that is, out‐of‐school exclusion and permanent exclusion (i.e., expulsion), the impact is small, positive and non‐statistically significant.

**Table 15:** Meta‐analysis by type of disciplinary measure.

Type of suspension	SMD	95% CI	*p*‐value	*n*	*k*	Measure of heterogeneity
General suspension	0.112	(0.027; 0.198)	< 0.01	52	203	*τ* ^2^_Level3_ = 0.059, *τ* ^2^_Level2_ = 0.015; *I* ^2^_Level3_ = 89%, *I* ^2^_Level2_ = 11%
In‐school	0.086	(−0.035; 0.209)	> 0.05	13	28	*τ* ^2^_Level3_ = 0.000, *τ* ^2^_Level2_ = 0.033; *I* ^2^_Level3_ = 31%, *I* ^2^_Level2_ = 66%
Out‐of‐school	0.075	(−0.029; 0.181)	> 0.05	14	29	*τ* ^2^_Level3_ = 0.000, *τ* ^2^_Level2_ = 0.033; *I* ^2^_Level3_ = 20%, *I* ^2^_Level2_ = 77%
Permanent exclusion	0.010	(−0.187; 0.208)	> 0.05	8	16	*τ* ^2^_Level3_ = 0.000, *τ* ^2^_Level2_ = 0.0; *I* ^2^_Level3_ = 20%, *I* ^2^_Level2_ = 4%

*General Suspension:* A large majority of the studies presented data on suspension as a broad and general measure. These studies did not describe operational definitions of the type of disciplinary suspensions involved in the outcome. We have defined this category as ‘general suspension’. For the sake of transparency, we report these results separately, although this measure could involve any of the previous outcomes reported above and, therefore, be a subset of the overall effect size reported at the beginning of this section.

**Figure 5:**
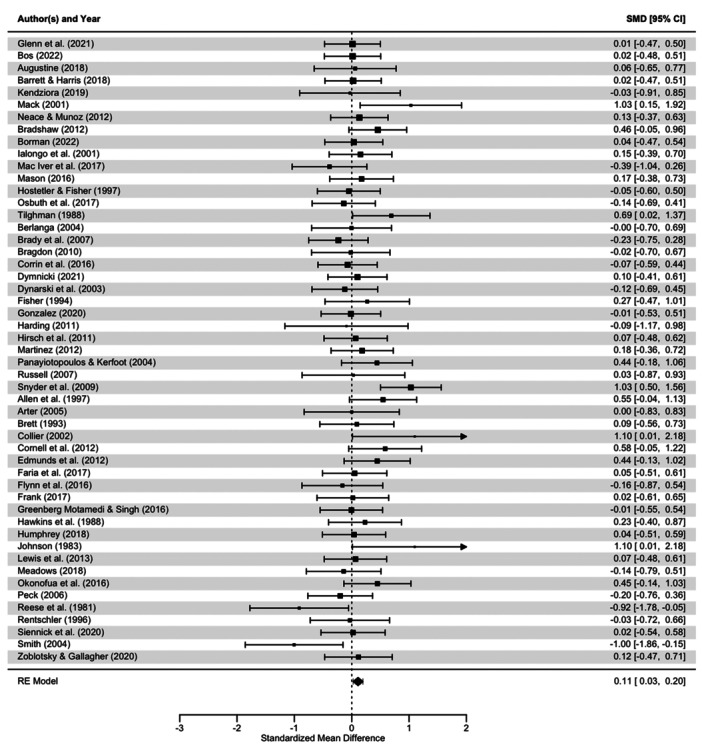
General suspension.

In total, 52 studies reported 203 effect sizes that were concerned with the impact of targeted interventions on general suspension. The mean effect was a small, positive and highly significant (SMD = 0.112 (95% CI: 0.027 to 0.198; *p* < 0.05). In addition, heterogeneity remained substantial (*Q* = 13,508; *df* = 202 *p* < 0.0001). The estimated variance components were *τ* ^2^_Level3_ = 0.059 and *τ* ^2^_Level2_ = 0.015. This means that *I* ^2^_Level3_ = 89% of the total variation can be attributed to between‐cluster, and *I* ^2^_Level2_ = 11% to within‐cluster heterogeneity (Figure 5).

*In‐School Suspension:* Thirteen studies (28 effect sizes) reported data on the impact of school‐based interventions on reducing in‐school suspension. Under a multilevel, random‐effects model with robust variance estimation, the effect of the intervention was small and non‐statistically significant. The final effect was SMD = 0.086 (95% CI: −0.035 to 0.209; *p* > 0.05). The heterogeneity was tested, showing significant variability across studies (*Q* = 7064.42; *df* = 27; *p* < 0.001). The estimated variance components were *τ* ^2^_level3_ = 0.0000 and *τ* ^2^_level2_ = 0.048. This means that *I* ^2^_Level3_ = 30% of the total variation can be attributed to between‐clusters. *I* ^2^_Level2_ = 66% was attributed to within‐cluster heterogeneity.

*Out‐of‐School Suspension:* Correspondingly, 14 studies reported 29 effect sizes for the impact of interventions on out‐of‐school suspension. Table 16 shows a small, non‐statistically significant effect of SMD = 0.075 (95% CI: −0.029 to 0.181; *p* > 0.05). The test for heterogeneity revealed the presence of large variation among the trials (*Q* = 111,176.281; *df* = 28; *p* < 0.001). The estimated variance components were *τ* ^2^_Level3_ = 0.000 and *τ* ^2^_Level2_ = 0.033. The *I* ^2^_Level3_ = 20% of the total variation can be attributed to between‐clusters, and *I* ^2^_Level2_ = 77% can be attributed to within‐cluster variation.

**Table 16:** Meta‐analysis: Impact of school‐based interventions on externalising behaviour.

Type of behaviour	SMD	95% CI	*n*	*k*	Measure of heterogeneity
Delinquency	0.073	(−0.163.309)	7	29	*τ* ^2^_Level3_ = 0.008, *τ* ^2^_Level2_ = 0.129; *I* ^2^_Level3_ = 22%, *I* ^2^_Level2_ = 75%
Violence	−0.0255	(−0.228; 0.176)	7	46	*τ* ^2^_Level3_ = 0.000, *τ* ^2^_Level2_ = 0.058; *I* ^2^_Level3_ = 23%, *I* ^2^_Level2_ = 44%
Conduct problems/antisocial	0.0141	(−0.159; 0.442)	18	51	*τ* ^2^_Level3_ = 0.303, *τ* ^2^_Level2_ = 0.010; *I* ^2^_Level3_ = 9%, *I* ^2^_Level2_ = 9%

*Permanent Exclusion (Expulsion):* The impact of school‐based interventions on permanent exclusion was small but non statistically significant. Table 16 shows that permanent exclusion was decreased by SMD = 0.010 (95% CI: −0.187 to 0.208; *p* > 0.05) with significant heterogeneity (*Q* = 48.38; *df* = 15; *p* < 0.001) based on only eight reports presenting data for analysis. The estimated variance components were *τ* ^2^_Level3_ = 0.000 and *τ* ^2^_Level2_ = 0.052. This means that *I* ^2^_Level3_ = 20% of the total variation can be attributed to between‐cluster and *I* ^2^_Level2_ = 4% to within‐cluster heterogeneity.

### 6.3 Secondary Outcomes

As stated in our protocol, for any identified study reporting data on school suspension we also coded, where possible, secondary outcomes referring to internalising and externalising behaviours.

We found a diverse range of measures referring to externalising behaviours, involving, for instance, substance misuse, violence, aggression, and problematic behaviour in school. To pool together comparable measures, we ran a meta‐analysis only on behaviours that could be categorised as antisocial, such as aggression, physical fights, delinquency, bullying and conduct disorder. An independent calculation was performed for behaviour involving the use of drugs.

**Figure 6:**
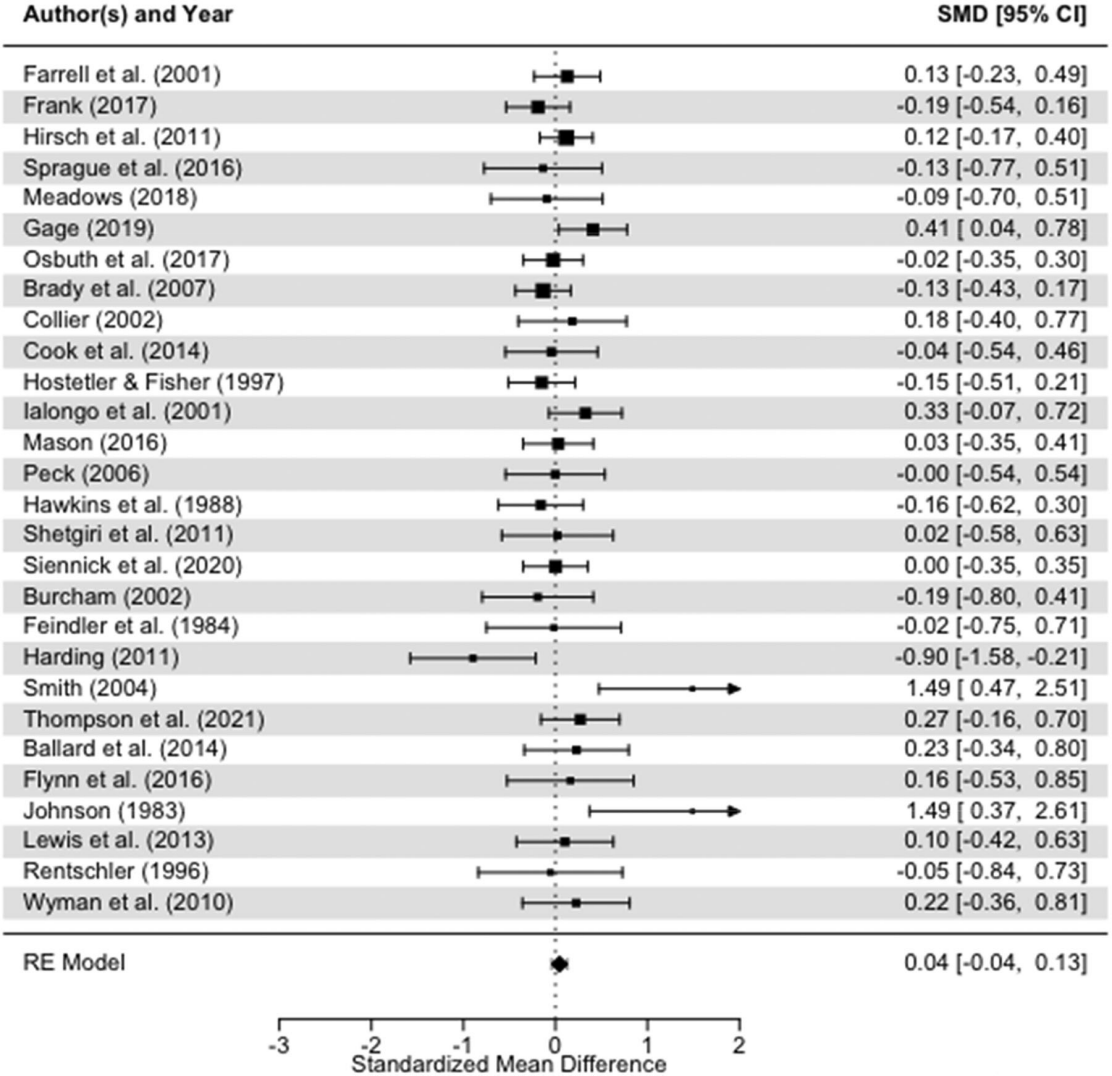
Impact of school‐based intervention on conduct problems.

*Conduct Problems:* The current analysis encompasses 126 effect sizes from 26 studies, all of which provide sufficient statistical information for a meta‐analysis. As reported in Figure 6, the overall impact of school‐based interventions on antisocial behaviour was not statistically different from zero SMD = 0.060 (95% CI: −0.065 to 0.186; *p* > 0.05), indicating an overall null effect of these programmes on reducing behavioural problems. Results showed significant heterogeneity (*Q* = 9993.76; *df* = 125; *p* < 0.001). The estimated variance components were *τ* ^2^_Level3_ = 0.039 and *τ* ^2^_Level2_ = 0.067. Subsequently, data show that *I* ^2^_Level3_ = 71% of the total variation can be attributed to between‐cluster (i.e., between studies) and *I* ^2^_Level2_ = 24% to within‐cluster heterogeneity (i.e., within studies).

It must be highlighted that some of the studies included in this overall measure reported negative effect sizes, meaning that in some specific cases the intervention had iatrogenic effects. For instance, Hawkins et al. (1988) reported the impact of an intervention focused on interactive teaching and co‐operative learning targeting low achievers in mainstream schools. The treatment group showed a reduction in the number of suspensions but an increase in the mean value for serious crime. The study reported by Hostetler and Fisher (1997) informed a similar case, Project CARE, aimed at increasing social skills to reduce risky behaviours. At post‐test, students in the treatment group reported significantly higher levels of problematic behaviours measured by the Negative Behaviour Scale (see p. 403). For its part, Obsuth et al. (2017) reported the results of a clustered RCT testing the impact of ‘Engage in Education‐London’. The intervention targeted high‐risk students, and it was aimed at improving communication and broader social skills. Results suggest that the iatrogenic effects were observable not only for exclusion but also for the case of antisocial behaviour. Finally, Frank et al. (2017) reporting the results of a yoga‐based social‐emotional wellness promotion programme, Transformative Life Skills, which showed a null effect for the primary and secondary outcomes, that is, exclusion and antisocial behaviour.

We further explore the impact of the included school‐based interventions on behavioural problems. For that aim, we decided to conduct three separate meta‐analyses for the outcomes grouped as delinquency, violence and conduct problems. As observed in Table 16, all effects were close to zero and none were statistically significant.

*Substance Use:* Eight studies and 42 effect sizes reported data involving substance use (e.g., alcohol consumption, marijuana, and other drugs). The overall impact of school‐based interventions on drug use was a negative but not statistically significant SMD = −0.055 (95% CI: −0.173 to 0.0632; *p* > 0.05), indicating an overall negative effect of these programmes on reducing substance use. Results showed significant heterogeneity (*Q* = 77.19; *df* = 41; *p* < 0.001). The estimated variance components were *τ* ^2^_Level3_ = 0.014 and *τ* ^2^_Level2_ = 0.008. When exploring the magnitude of the variation, data suggest that *I* ^2^_Level3_ = 31% of the total variation can be attributed to between‐cluster (i.e., between studies) and *I* ^2^_Level2_ = 19% to within‐cluster heterogeneity (i.e., within studies).

### 6.4 Explaining Heterogeneity: Moderator Analysis

Heterogeneity, or the variation observed among studies, can be characterised as non‐random variability. This variation can arise from several factors, such as differences in participant demographics or variations in intervention dosage (e.g., low vs. high). Given the broad scope of our study, which focuses on school‐based interventions, we anticipated encountering significant heterogeneity across the included studies. The empirical findings indeed confirmed our expectations in this regard. It is important to note that the presence of mean effect sizes accompanied by high heterogeneity does not inherently weaken the meta‐analysis. In fact, heterogeneity can be indicative of subgroup effects, which, in turn, can help answer crucial questions related to the effectiveness of interventions under specific conditions and for particular populations. These questions are of paramount importance for evidence‐based prevention efforts (Groenwold et al. 2010).

As suggested by Lipsey and Wilson (2001), meta‐analysis encompasses two distinct categories of variables: (a) effect sizes, which typically constitute the primary focus and serve as the dependent variables in meta‐analysis, and (b) descriptive variables that characterise both the effect sizes and the studies responsible for generating them, serving as the independent variables in meta‐analysis.

Building on previous research, our pre‐defined moderators encompass four key aspects: (i) demographic characteristics of the participants, (ii) methodological characteristics of the studies, (iii) the role of the evaluator, and (iv) characteristics of the interventions.

#### 6.4.1 Effects Moderated by Participants' Demographic Characteristics

For studies reporting data on gender, we ran a moderator analysis aimed at understanding variations between male and female students. As observed in Table 17, only four studies reported data adjusted by sex. Based on the meta regression analysis, the impact of the intervention was slightly higher for male students, however the difference for both groups was non statistically significant (*F* = 0.243; *df*_1_ = 1; *df*_2_ = 8; *p* > 0.05). This analysis only included the impact on female and male students. Overall effect sizes were excluded to the aim of capturing the differential impact by sex.

**Table 17:** Moderator analysis (gender).

Sex	SMD	95% CI	*n*	*k*	*Q*	*df*	*τ* ^2^_Level3_	*τ* ^2^_Level2_	*I* ^2^_Level3_	*I* ^2^_Level2_
Female	0.268	(−0.207; 0.744)	4	10	342.4	8	0.137	0.008	93%	6%
Male	0.308	(−0.142; 0.225)								

In terms of age, the best proxy variable was school grade (Table 18). This information was reported in 48 studies reporting 173 effects. To test the hypothesis that effect sizes vary by age, we ran a moderator analysis for studies involving primary (SMD = 0.174; 95% CI: 0.016 to 0.332; *p* < 0.05), middle school students (SMD = 0.133; 95% CI: −0.249 to 0.166; *p* > 0.05), and high school students (SMD = 0.060; 95% CI: −0.370 to 0.143; *p* > 0.05). The impact of the intervention was small, positive and statistically significant for students in primary school. However, the between‐effect difference was not statistically significant (*F* = 0.3799; *df*_1_ = 2; *df*_2_ = 170; *p* > 0.05).

**Table 18:** Moderator analysis (grade).

Grade at school	SMD	95% CI	*n*	*k*	*Q*	*df*	*τ* ^2^_Level3_	*τ* ^2^_Level2_	*I* ^2^_Level3_	*I* ^2^_Level2_
Primary school	0.174	(0.016; 0.332)*	48	53	7646.6	170	0.070	0.020	76%	23%
Middle school	0.133	(−0.249; 0.166)		68						
High school	0.060	(−0.370; 0.143)		52						

* *p* < 0.05.

We also coded data identifying the country of the selected sample in each primary studies included in the present multilevel meta‐analysis. As observed in Table 19, a large proportion of the studies were implemented in the United States, with only 3 out of 67 focusing in the United Kingdom. The effect in both countries was small and the effect was not statistically significant (*F* = 0.007; *df*_1_ = 1; *df*_2_ = 265; *p* > 0.05).

**Table 19:** Moderator analysis (country).

Country	SMD	95% CI	*n*	*k*	*Q*	*df*	*τ* ^2^_Level3_	*τ* ^2^_Level2_	*I* ^2^_Level3_	*I* ^2^_Level2_
United Kingdom	0.094	(−0.224; 0.412)	67	6	9032.65	265	0.065	0.008	88%	11%
United States	0.108	(−0.312; 0.341)		261						

#### 6.4.2 Effects Moderated by Characteristics of the Studies

We extracted data on characteristics of the studies, such as the methodological design used to calculate the impact of school‐based interventions on school exclusion. In this updated version of the review, we included RCTs but also quasi‐experimental studies. Table 20 suggests that the highest impact was detected using experimental designs (i.e., RCTs). In the case of quasi‐experiments, the impact was close to zero. None of the effects were statistically significant and differences by moderator analysis showed non‐significant differences (*F* = 0.631; *df*_1_ = 2; *df*_2_ = 271; *p* > 0.05).

**Table 20:** Moderator analysis (study design).

Study design	SMD	95% CI	*n*	*k*	*Q*	*df*	*τ* ^2^_Level3_	*τ* ^2^_Level2_	*I* ^2^_Level3_	*I* ^2^_Level2_
QED	0.061	(−0.154; 0.277)	274	20	8262.1	271	0.062	0.011	85%	15%
QED (PSM)	0.029	(−0.314; 0.251)		77						
RCT	0.133	(−0.158; 0.303)		177						

We coded data identifying the role of the evaluator (Table 21). Independent evaluators were those not taking part in the design or implementation of the evaluated programme. Dependent evaluators were those also contributing to the design and/or the implementation of the programme. For this analysis, we included 52 trials and 206 effect sizes. Evaluations carried out by independent evaluators produced the lowest impact SMD = 0.055 (95% CI: −0.16 to 0.176; *p* > 0.05). Unsurprisingly, based on what is known about developer‐led trials, the studies conducted by dependent evaluators (i.e., those who also developed and/or designed the intervention) produced better results. In fact, trials where the evaluator designed and delivered the intervention yielded results where SMD = 0.436 (95% CI: 0.096 to 0.680; *p* < 0.001). The meta‐regression analysis demonstrated that effect sizes are lower for studies conducted by independent evaluators. The test of moderators showed differences were statistically significant (*F* = 4.34; *df*_1_ = 2; *df*_2_ = 203; *p* < 0.0001).

**Table 21:** Moderator analysis (evaluator role).

Evaluator role	SMD	95% CI	*n*	*k*	*Q*	*df*	*τ* ^2^_Level3_	*τ* ^2^_Level2_	*I* ^2^_Level3_	*I* ^2^_Level2_
Design	0.047	(−0.106; 0.202)	52	25	8318.9	203	0.028	0.014	65%	32%
Design and delivery	0.436	(0.096; 0.680)**		14						
Independent	0.055	(−0.160; 0.176)		167						

** *p* < 0.01.

#### 6.4.3 Effect of Different School‐Based Programmes on School Suspension

As a result of the systematic searches, we found a range of school‐based interventions, each with distinct objectives and methods. Some interventions focused on implementing changes at the school‐wide level. For instance, programmes like School‐Wide Positive Behavioural Interventions and Supports (SWPBIS) adopted a tiered approach, which included implementing strategies for the entire school community and also for those with more specific risk and needs. Another subset of studies reported the impact of interventions targeting changes at the level of students' behaviour, for instance, skills training for students or cognitive behavioural treatments for reducing violence. Conversely, another category of interventions aimed to enhance teachers' skills and competencies. To explore potential differences in their effectiveness, we conducted a moderator analysis comparing school‐wide, student‐centred, and teacher‐centred interventions. Table 22 suggests that interventions centred on changes at the school level or teacher level were slightly more effective than interventions centred on students' behaviour. Even if school centred intervention reported significant results, the test of meta‐regression showed differences were not statistically significant (*F* = 0.652; *df*_1_ = 2; *df*_2_ = 263; *p* < 0.05).

**Table 22:** Moderator analysis (targeted level of intervention).

Targeted level intervention	SMD	95% CI	*n*	*k*	*Q*	*df*	*τ* ^2^_Level3_	*τ* ^2^_Level2_	*I* ^2^_Level3_	*I* ^2^_Level2_
School	0.170	(0.026; 0.314)*	65	94	8933.8	263	0.064	0.011	85%	15%
Students	0.082	(−0.257; 0.081)		159						
Teachers	0.179	(−0.29; 0.31)		13						

* *p* < 0.05.

One of the aims of the present meta‐analysis was to compare the effect of different interventions on the reduction of school suspension. Table 23 presents the SMDs, confidence intervals as well as measures of heterogeneity for each of the nine types of programmes included in the review.

**Table 23:** Moderator analysis (intervention type).

Intervention type	SMD	95% CI	*n*	*k*	*Q*	*df*	*τ* ^2^_Level3_	*τ* ^2^_Level2_	*I* ^2^_Level3_	*I* ^2^_Level2_
Enhancing Academic skills	0.11	(−0.13; 0.35)		9	8077.81	258	0.05	0.01	84%	16%
Mentoring/monitoring	−0.04	(−0.48; 0.18)		17						
Mental Health	0.31	(−0.14; 0.54)		10						
Parent skills	0.17	(−0.50; 0.62)		4						
Restorative justice	0.14	(−0.35; 0.40)		54						
Risk reduction	−0.06	(−0.52; 0.17)		11						
Student skills	−0.03	(−0.44; 0.13)		34						
School‐wide	0.18	(−0.22; 0.35)		84						
Teacher skills	0.18	(−0.29; 0.42)		13						
Violence reduction	0.31	(−0.13; 0.53)		32						

First of all, as observed in Table 23, many of the interventions are represented by a restricted number of studies/effect sizes, and therefore these results should be interpreted with caution.

Second, the SMDs of four types of programmes present positive (small) and not statistically significant results in favour of the reduction of school exclusion. Those programmes are categorised as: (i) mental health; (ii) violence reduction; (iii) teacher skills; (iv) school‐wide interventions.

Third, to test the hypothesis that differences were significant among the compared sub‐types, we ran the moderator analysis. The comparison demonstrates that differences are not statistically significant (*F* = 0.652; *df*_1_ = 9; *df*_2_ = 1.512; *p* < 0.05), meaning that variation in effect sizes cannot be explained by the type of intervention.

Finally, we run a multiple‐predictor meta‐regression using the metafor package in r‐studio. The regression analysis included the role of the evaluator, grade at school, percentage of male students and a variable representing the score of RoB for each included study. The results suggested one statistically significant finding. The intervention effect was larger for interventions centred on teachers' skills (*b* = 0.77, *p* > 0.01). However, it is important to keep in mind that the number of studies centred on teachers' skills improvement was limited in this study (i.e., 4 studies and 10 effect sizes).

### 6.5 Publication Bias Analysis

Publication bias in systematic reviews occurs when the included set of manuscripts fails to systematically represent the whole population of completed studies that should have been included. The whole population of studies can involve a range of results that must be present in a meta‐analysis to make it valid. However, consistent evidence indicates that studies presenting large effects are more likely to be published than those presenting null or modest effects (Rothstein et al. 2006).

**Figure 7:**
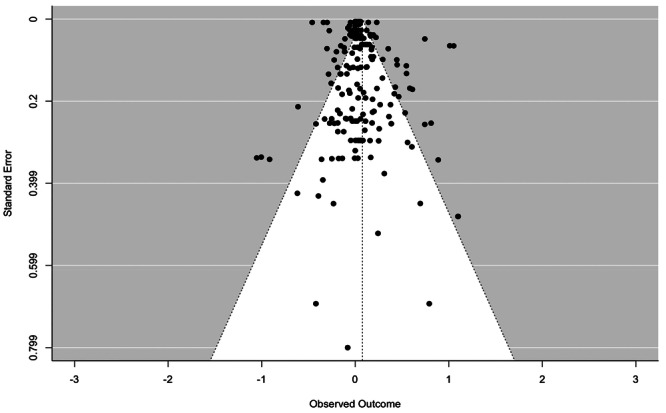
Funnel plot (publication bias).

As originally proposed, we use statistical procedures to quantify potential bias that could affect our analysis. First, we produced a funnel plot of SEs by standardised differences in means, presented in Figure 7. In theory, symmetrical distributions of dots under the funnel represent a normal distribution of studies. As anticipated, our studies mostly fall under the funnel, and they are distributed around the main effect. However, since the evaluation of funnel plots can be subjective, we conducted additional statistical measures of publication bias, specifically, Duval and Tweedie's trim‐and‐fill analysis.

**Figure 8:**
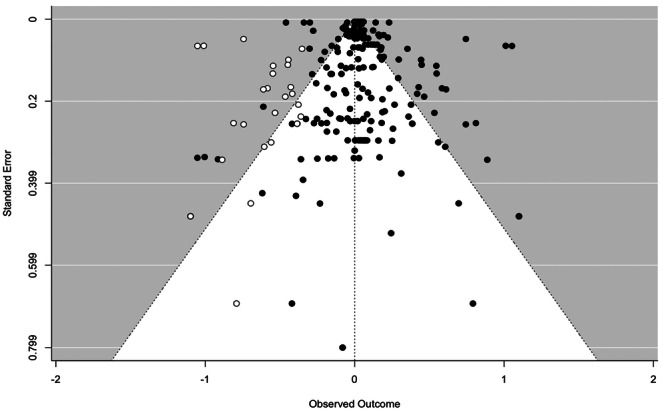
Duval and Tweedie's trim‐and‐fill analysis.

Duval and Tweedie's trim‐and‐fill analysis compares the differences in effect sizes that could potentially be attributed to bias (Figure 8). The technique imputes effect sizes until the error distribution gets close to normality. In this way, the test offers the best estimate of the unbiased effect (Rothstein et al. 2006). Results of Duval and Tweedie's trim‐and‐fill analysis suggest that there were differences in effect sizes attributable to bias.

This result suggests that there are an estimated 34 missing studies on the left side of the funnel plot (white dots). These are studies that, based on the observed asymmetry in the funnel plot, are assumed to be missing from the analysis. The SE associated with this estimate is 10.1008. The analysis is conducted using an equal‐effects model, which implies that all studies in the meta‐analysis are assumed to have a common true effect size. The total number of studies included in the analysis (*k*) is 276. The Trim‐and‐Fill method has been used to adjust for potential publication bias by estimating missing studies on the left side of the funnel plot, but the effect size estimate remains close to zero and non‐significant. To assess the presence of small‐study effects, we conducted an Egger‐type meta‐regression by regressing SMDs (Cohen's *d* or SMD) on their SEs. A multilevel meta‐analytic model was fitted using restricted maximum likelihood estimation, including random effects at the study and within‐study level (author/id). Robust SEs were computed using the clubSandwich CR2 estimator, with clustering at the study level (author), to account for dependence among effect sizes.

The regression coefficient for SE was positive and statistically significant (*β* = 0.437, robust SE = 0.158, *t*(56.1) = 2.77, *p* = 0.007), suggesting evidence of small‐study effects. The 95% confidence interval ranged from 0.121 to 0.753. These results indicate potential funnel plot asymmetry and raise the possibility of publication bias. The intercept term was not statistically significant, further suggesting that the observed asymmetry is primarily related to the precision of the studies rather than systematic inflation of effect sizes.

### 6.6 Sensitivity Analysis

The *r* parameter (ICC) is often used to model the dependence between effect sizes from the same cluster or study. We corrected the value of the variance by assuming a value of correlation equal to 0.80. To test the robustness of this assumption, we ran sensitivity analysis with a correlation equal to 0.60. Table 24, panel A, shows that overall results remain stable when the correlation is smaller.

**Table 24:** Sensitivity analysis.

Covariate	SMD	95% CI	SE	*n*	*k*		
Panel A: With outliers Windsorised							
0.80	0.106	0.037; 0.175**	0.034	67	276	***τ*** ^**2**^_**Level3**_ = 0.044, ***τ*** ^**2**^_**Level2**_ = 0.021	
0.60	0.106	0.037; 0.175**	0.034	67	276	***τ*** ^**2**^_**Level3**_ = 0.048, ***τ*** ^**2**^_**Level2**_ = 0.014	
Panel B: With outliers							
0.80	0.106	0.037; 0.175**	0.034	67	276	***τ*** ^**2**^_**Level3**_ = 0.044, ***τ*** ^**2**^_**Level2**_ = 0.021	
0.60	0.106	0.037; 0.175**	0.034	67	276	***τ*** ^**2**^_**Level3**_ = 0.048, ***τ*** ^**2**^_**Level2**_ = 0.014	

** *p* < 0.01.

Another decision was related to the presence of outliers. We found eight studies presenting an effect size more than three standard deviations from the mean effect size, which was defined as an outlier. We tested the impact of the outlier and also the impact of winsorisation (Lipsey and Wilson 2001). The size of the effects, their direction and their significance were similar. See Table 24, panel B.

## 7 Results: Qualitative Evidence Synthesis

### 7.1 Description of Studies

#### 7.1.1 Results of the Search

Overall, our electronic database searches for qualitative process evaluations of school‐based interventions to reduce school exclusions returned 13,733 search results. Search results were first uploaded to Endnote for title and abstract screening. The software identified 3517 duplicates and a further 51 were removed manually.

Therefore, 10,165 studies were screened by title and abstract. These studies were screened by one screener, and 50% were screened by H.G. and 50% were screened by S.H. using Endnote. The majority of studies were excluded based on their title and abstract (*n* = 10,070), and a total of 95 studies were retained for full‐text screening. One additional study was identified in our supplementary searches (i.e., Ashworth 2018 was identified in hand searches of the EthOS database), bringing the total to 96 studies screened at full text.

**Figure 9:**
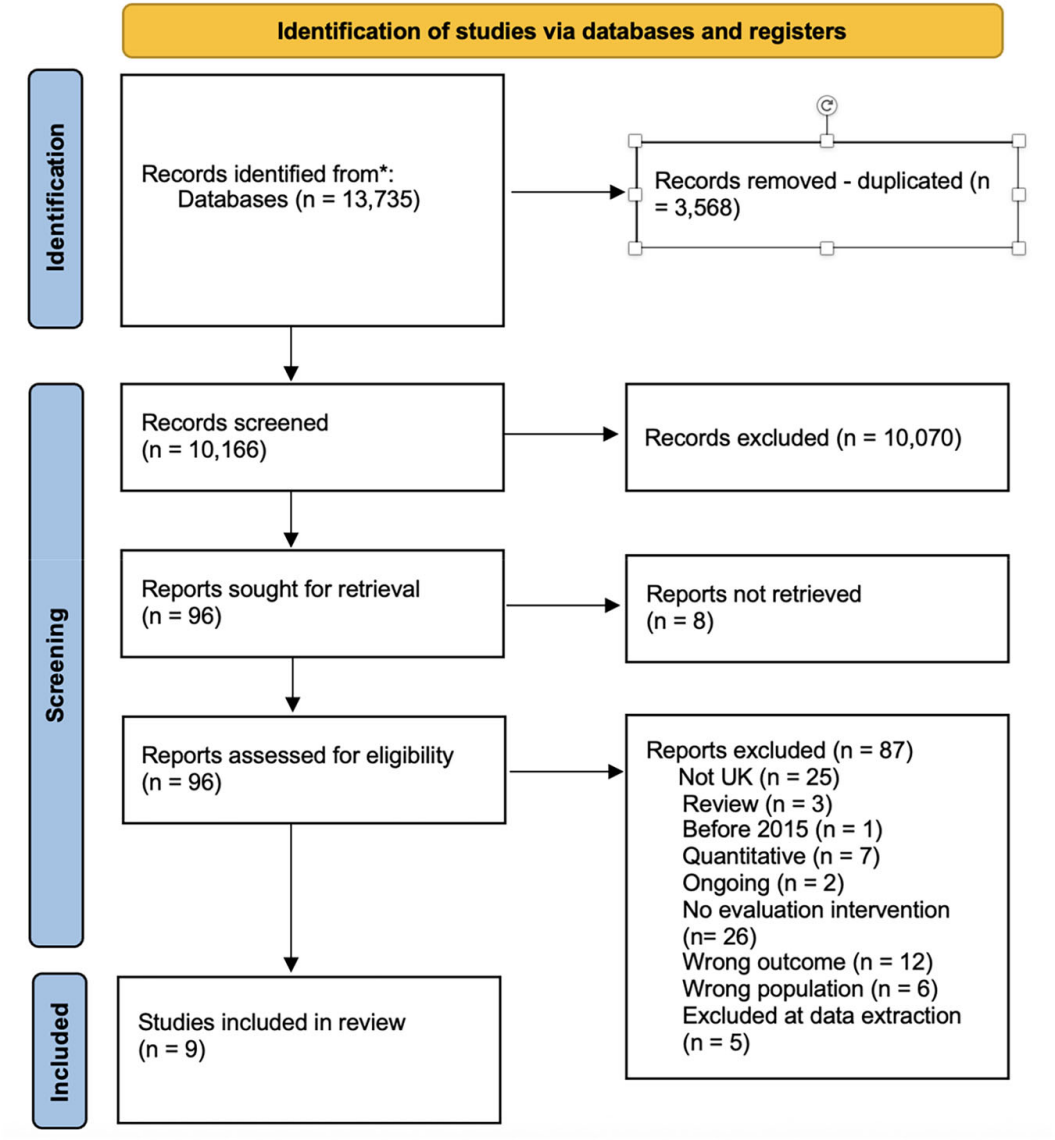
PRISMA flowchart for qualitative searches. *Source:* Page et al. 2021. *BMJ* 372: n71. https://doi.org/10.1136/bmj.n71.

All studies were retrieved and screened based on the full text by two members of the research team independently, and conflicts were resolved in discussion (H.G. and S.H.). Full text screening was conducted in Covidence, and the results are represented in Figure 9. The majority of conflicts arose due to selecting different reasons for exclusion, as even at full‐text screening, there were often multiple reasons to exclude a study. The inter‐rater reliability was (0.75) and the proportion agreement was (93.6%).

Most studies were subsequently excluded at this stage (*n* = 82), the majority of which were studies that discussed school exclusions in the United Kingdom but did not evaluate an intervention (*n* = 26) or studies that were conducted outside of the United Kingdom (*n* = 25). Other reasons for exclusion included, wrong outcomes (i.e., did not measure/discuss school exclusions or student disruptive behaviour; *n* = 12), quantitative study (*n* = 7), review (*n* = 3), wrong population (i.e., not mainstream school students or teachers or school staff; *n* = 6), protocol study (*n* = 2), or the study was published before 2015 (*n* = 1).

As such, 14 studies were retained for inclusion in the qualitative evidence synthesis (i.e., Allen et al. 2023; Ashworth 2018; Blandford‐Elliott 2021; Calcutt 2020; Humphrey et al. 2018; Knowler et al. 2019; Marchant et al. 2019; Middleton 2022; Reynolds 2021; Rivers 2016; Stanbridge and Campbell 2016; Sparling et al. 2022; Warren et al. 2020; Wright 2020).

However, five studies were subsequently excluded at data extraction phase (Calcutt 2020; Knowler et al. 2019; Marchant et al. 2019; Reynolds 2021; Rivers 2016). These studies are described in further detail in Section 7.1.5, alongside an explanation for their exclusion following data extraction.

In total, nine process evaluations were therefore included in our qualitative evidence synthesis (i.e., Allen et al. 2023; Ashworth 2018; Blandford‐Elliott 2021; Humphrey et al. 2018; Middleton 2022; Stanbridge and Campbell 2016; Sparling et al. 2022; Warren et al. 2020; Wright 2020). Ashworth (2018) and Humphrey et al. (2018) drew on findings from participants involved in the same trial. The characteristics and features of these nine process evaluations are summarised in Table 25. In addition, a brief narrative summary of each evaluation, with a specific focus on the intervention implemented, is outlined in Appendix S1F.

**Table 25:** Summary of included process evaluations.

Author	Type of publication	Name of intervention	Sample characteristics	Data collection	Analysis
Allen et al. (2020)	Journal article	Incredible Years teacher classroom management programme	44 teachers that took part in the STARS trial. The majority of the sample were identified as female (77%) and the mean age for focus groups was 34.6 years old. The mean age for interviewed teachers was 30.3 years old. The mean years in teaching was 6–7 years. Three teachers were members of school leadership teams, and two were classified as NQT.	Focus groups, lasting approximately 90 min and coordinated by a trained research‐facilitator with 31 teachers. Telephone interviews were offered to participants who could not attend focus groups (*n* = 13). Data saturation was achieved after five focus groups.	Thematic analysis
Ashworth (2018)	Thesis	Good Behaviour Game	Participants were primary school teachers, members of school leadership teams and students. Demographic information about participants is not reported. Sample was the same sample as included by Humphrey et al. (2018).	Semi‐structured interviews and focus groups in the six case study schools. Focus groups with staff members, such as teachers or members of school leadership lasted approximately 30 min and focus groups with students lasted 20 min approximately.	Thematic analysis
Blandford‐Elliott (2021)	Thesis	SWPBIS	Participants were headteachers (*n* = 3), members of school leadership teams (*n* = 2), class teachers (*n* = 5), deputy head teachers (*n* = 2) and teaching assistants (*n* = 1). The majority of participants were identified as female (85%).	Virtual semi‐structured interviews, lasting 18–54 min. Interviews were online due to COVID‐19 lockdowns.	Thematic analysis
Humphrey et al. (2018)	Research report	Good Behaviour Game	Participants were primary school teachers, members of school leadership teams and students. Demographic information about participants is not reported. Sample was the same sample as included by Ashworth (2018).	Interviews, focus groups, observations and document analysis in six case study schools. Visits to case study schools to collect data occurred before and during implementation of the intervention.	Thematic analysis
Middleton (2022)	Journal article	NurtureUK Violence Reduction Unit	Participants were from mainstream primary and secondary schools in Kent (Medway) and London, and one alternative provision setting. Participants were members of school leadership (*n* = 3) or heads of departments (*n* = 2). Participants were also identified as Inclusion Leaders (*n* = 1), social‐emotional and mental health lead (*n* = 1) and nuture lead (*n* = 1).	Virtual focus groups with groups of participants across participating settings. Three focus groups were run and lasted approximately 90 min. Groups 1 and 2 had three participants, group 3 had two participants.	Deductive thematic analysis
Stanbridge and Campbell (2016)	Journal article	Planning interventions	19 students and staff members from two rural schools were interviewed. No information about student or staff demographics is reported.	Semi‐structured interviews.	Thematic analysis following an inductive approach to interpretation.
Sparling et al. (2022)	Journal article	ACE‐informed schools	Demographic information about purposefully sampled participants is not provided.	Semi‐structured interviews that took 24–72 min. There were 3 phases of data collection, individual interviews with project team members, interviews with head teachers, and a follow‐up focus group with project team members.	Thematic content analysis
Warren et al. (2020)	Journal article	Learning Together whole‐school health‐based intervention	Participants were members of staff leading the intervention, staff leading the social‐emotional learning curriculum, students involved in the project action group, staff members of the action group, and two students involved in restorative conversations as part of the intervention. Demographic information is not provided.	Telephone interviews with 45 staff members and students across 3 case study schools, in the first 2 years of interventions. Three annual focus groups were conducted with school staff and students. Interviews and focus groups lasted 15–90 min. Researcher also kept field notes.	Thematic content analysis
Wright (2020)	Thesis	Risk Avert	Participants were categorised as being employed in student support roles (*n* = 7), teachers (*n* = 1), and members of school leadership teams (*n* = 1). Four participants were labelled as non‐teaching staff and all participants had been in their current role for less than 15 years. Participants were identified as mostly female (*n* = 7; 78%). Participants were also mostly identified as White (*n* = 8; 89%).	Semi‐structured interviews with staff across eight schools, lasting between 20 and 73 min. Researcher also kept field notes.	Thematic analysis

Abbreviations: NQT, newly qualified teacher; SWPBIS, School‐wide Positive Behavioural Intervention and Support.

#### 7.1.2 Characteristics of Included Studies

A variety of interventions were evaluated in the qualitative process evaluations included in the present review. Two studies examined participants' perceptions of the Good Behaviour Game (GBG), an intervention developed in the United States but implemented globally primarily in primary schools (Ashworth 2018; Humphrey et al. 2018). The findings reported in these separate studies are from the same evaluation of the GBG in English primary schools. Other interventions included the Incredible Years teacher classroom management programme (Allen et al. 2020), the Risk Avert screening tool (Wright 2020), the nurtureUK Violence Reduction Unit (VRU) programmes (Middleton 2022), an intervention planning and screening tool (multi‐element plans [MEPS] and target‐monitoring and evaluation [TME]; Stanbridge and Campbell 2016), School‐Wide Positive Behavioural Supports (SWPBIS; Blandford‐ElliotT 2021); a health‐based intervention ‘Learning Together’ (Warren et al. 2020) and a trauma‐informed whole‐school intervention (Sparling et al. 2022). Narrative summaries of each evaluation with a specific focus on the intervention evaluated are provided in Appendix S1F.

The majority of qualitative evaluations were undertaken in English schools (*n* = 7), with one evaluation in Welsh schools (i.e., Blandford‐Elliott 2021), and one evaluation did not provide the geographical location of schools, beyond that they were in the United Kingdom (i.e., Stanbridge and Campbell 2016). Most included process evaluations were of interventions implemented in primary schools (*n* = 5). Two evaluations (i.e., Warren et al. 2020; Wright 2020) assessed interventions implemented in secondary schools and one evaluation included participants from both primary and secondary schools (i.e., Middleton 2022). One evaluation included participants from an alternative provision setting (Middleton 2022).

Generally, included process evaluations included perspectives from teachers, school leadership (e.g., headteachers or deputy headteachers), or other members of school staff (e.g., teaching assistants). Only one evaluation included perspectives from teachers only (i.e., Allen et al. 2020), but as this programme was considered a teacher training intervention, this is appropriate. Furthermore, only three process evaluations included perspectives of students from the students themselves (i.e., Ashworth 2018; Humphrey et al. 2018; Warren et al. 2020).

All included process evaluations used thematic analysis, or a variation of this approach, to code their findings and the majority used a combination of focus groups and interviews to collect data (*n* = 4). Three process evaluations used interviews only (i.e., Blandford‐Elliott 2021; Warren et al. 2020; Wright 2020). Process evaluations were published between 2016 and 2022 and mostly in peer‐reviewed journal articles (*n* = 4). Three theses were included and one research report (i.e., Humphrey et al. 2018).

#### 7.1.3 Assessment of Quality of Process Evaluations

The CASP tool for quality appraisal in qualitative evidence synthesis was used in the present review to examine the quality of included process evaluations. The items included in this tool were previously outlined in Table 8 (see Section 4.4.12).

This tool is recommended for novice researchers in qualitative evidence synthesis, and experts have noted that some modifications are necessary (Long et al. 2020). Thus, in the context of the present review, we often felt that the ‘can't tell’ response option was unclear and inappropriately added ambiguity to the assessment. As such, we modified this to a ‘somewhat’ criteria whereby when the response was not a clear ‘yes’ or a clear ‘no’, an appropriate option was available. The addition of a fourth response option in this vein is endorsed by other users (e.g., Long et al. 2020).

As previously outlined, the CASP checklist includes 3 sections and 10 questions related to various aspects of qualitative studies. Appendix S1H provides a detailed breakdown of our assessments for the included process evaluations. Two members of the research team conducted an assessment of quality for the included process evaluations, and the mean percentage agreement across all nine studies was 66%. Overall, the result suggests that the quality of the included qualitative process evaluations was good. The following sections narratively describe the findings.

Section A: Are the results valid?

1. *Was there a clear statement of the aims of the research?*
  This item requires the assessor to consider the goals, importance, and relevance of the study. The majority of evaluations (*n* = 7) did include a clear statement of the aims of the process evaluation, and only two evaluations (*n* = 2; Blanford‐Elliott 2021; Wright 2020) did not. In the case of Blandford‐Elliott (2021), this study scored a ‘no’ because while the aims were outlined, the relevance and goals of the qualitative strand of this evaluation were not. Wright (2020) did not clearly outline the aims, over and above referring to the need for more qualitative studies.
2. *Is a qualitative methodology appropriate?*
  This item requires the assessor to consider the suitability of qualitative research as an approach to their research questions and aims. All but one process evaluation was marked as a ‘yes’ for this question on the (Critical Appraisal Skills Programme 2024) checklist. Stanbridge and Campbell (2016) were marked ‘somewhat’ in relation to the appropriateness of a qualitative methodology. This was a mixed‐methods evaluation, and the reasons for including a qualitative analysis were not clearly defined. The rationale for including a qualitative methodology alongside their quantitative assessments should have been provided.
3. *Was the research design appropriate to address the aims of the research?*
  This item requires the assessor to consider whether or not the researcher has justified the qualitative approach and provided an explanation for why a particular method was used. The majority of process evaluations included in our qualitative evidence synthesis did address this item sufficiently (*n* = 6), but three were marked as ‘somewhat’ (i.e., Allen et al. 2020; Sparling et al. 2022; Stanbridge and Campbell 2016). Stanbridge and Campbell (2016) were assessed in this way for similar reasons as provided for question 2. Allen et al. (2020) and Sparling et al. (2022) were marked as ‘somewhat’ because the reasons for conducting focus groups, instead of interviews for example, was not provided.
4. *Was the recruitment strategy appropriate to the aims of the research?*
  This item requires the assessor to consider whether or not the recruitment of participants was sufficient. The majority of process evaluations (*n* = 6) were assessed as a ‘yes’ on this item, as they provided appropriate detail about why schools, teachers, or groups of teachers were recruited to the study. Moreover, process evaluations that used purposively sampled participants (e.g., Middleton 2022) explained sufficiently why these participants were targeted and what the implications were for these decisions. Three evaluations were assessed as a ‘no’ on this item, due to missing information about recruitment (i.e., Allen et al. 2020; Blandford‐Elliott 2021; Sparling et al. 2022).
5. *Was the data collected in a way that addressed the issue?*
  This item requires the assessor to evaluate the description and detail provided about data collection methods. All but one process evaluation (i.e., Stanbridge and Campbell 2016) was marked as a ‘yes’ on this item due to sufficient reporting of the format and approach to interviews/focus groups, data transcription and other logistical factors. Stanbridge and Campbell (2016) was marked as a ‘somewhat’ due to the limited information provided.
6. *Has the relationship between researcher and participants been adequately considered?*
  This item requires the assessor to consider whether or not a study has appropriately described the relationship between the researcher conducting interviews and/or focus groups and participants. This is important because participants' responses may vary depending on the nature or degree of a relationship and familiarity with the researcher. Only two evaluations (i.e., Allen et al. 2020; Blandford‐Elliott 2021) were marked as a ‘yes’ on this question. All other evaluations failed to describe the relationship between participants and researcher.
  Section B: What are the results?
7. *Have ethical considerations been taken into consideration?*
  This item requires the assessor to examine whether any relevant ethical issues, such as informed consent or participant anonymity, have been taken into consideration. Only one evaluation (i.e., Stanbridge and Campbell 2016) was marked as a ‘no’ on this item, as the report failed to refer to any ethical procedures or consent forms.
8. *Was the data analysis sufficiently rigorous?*
  This item requires the assessor to examine the data analysis and the approach to interpreting qualitative evidence. Issues like the robustness of thematic analysis, the consideration of saturation and differing findings, and the possible bias from the researcher are considered. The majority of process evaluations were marked as a ‘yes’ on this item (*n* = 6), despite the general lack of consideration of any bias on behalf of the researcher and discussion of saturation. Sparling et al. (2022) was marked as a ‘no’ because there was a lack of supporting quotations and Stanbridge and Campbell (2016) was also marked as a ‘no’. In the case of Stanbridge and Campbell (2016), this was due to a lack of information about the actual analysis and a description of the analytical approach.
9. *Is there a clear statement of findings?*
  This item requires the assessor to consider whether the study has sufficiently summarised and interpreted the findings. Almost all process evaluations included in our qualitative evidence synthesis were marked as a ‘yes’ (*n* = 7), with one marked as a ‘no’ (i.e., Humphrey et al. 2018) and one marked as a ‘somewhat’ (i.e., Blandford‐Elliott 2021). Humphrey et al. (2018) was marked down as this was a mixed‐methods evaluation, and the summary of findings related mostly to the quantitative results. Blandford‐Elliott (2021) was marked down due to a lack of information.
  Section C: Will the results help locally?
10. *How valuable is the research?*
  This final item on the CASP checklist requires the assessor to examine whether the results and findings are adequately contextualised and described in terms of future research and policy impact. Five process evaluations were marked as a ‘yes’, due to sufficiently describing the value and relevance of the findings not only to future research on the specific intervention, but also more broadly across school‐based interventions and in the United Kingdom or global schools. Three process evaluations were marked as ‘somewhat’ because they only discussed the implications for future research on the specific intervention (i.e., Blandford‐Elliott 2021; Stanbridge and Campbell 2016; Wright 2020), and one was marked as a ‘no’ because no implications or policy relevance were discussed specifically in relation to the qualitative findings (i.e., Humphrey et al. 2018).

#### 7.1.4 Perceptions of Impact on School Exclusions

As previously discussed, there was a parity of process evaluations that met our inclusion criteria. The most relevant study we identified in our systematic searches was the mid‐programme progress report by Middleton (2022). This study used a focus group methodology to explore the effectiveness of nurtureUK VRU programmes implemented in London, Kent, and Medway. The findings on the facilitators and barriers to implementation of these programmes are discussed in full in Section 7.2, but a brief description of the findings in relation to impact on school exclusions is provided here due to the relevance to the current mixed methods review and the UK policy context.

This process evaluation looked at programmes informed by ‘nurture practice’, a form of trauma‐informed intervention. A full review of this approach is beyond the scope of the present review, but a brief overview is included in Appendix S1F. Middleton (2022) conducted focus groups with project leaders who led school‐based restorative and nurturing programmes between 2020 and 2022. Nine participants took part, including senior leadership from mainstream secondary schools and alternative provision institutions. Using thematic analysis, Middleton (2022) found that overall, the participants perceived the intervention as effective in reducing school exclusions. One participant noted:

We have used [nurturing practice] in a small area to begin with and have seen a reduction in verbal abuse towards staff, whilst another outlined: Two boys who were looking at permanent exclusion now come in … and are doing so well.

(Middleton 2022, 76)

#### 7.1.5 Excluded Studies: Process Evaluations

Fourteen studies were originally retained for inclusion in our qualitative evidence synthesis, but during data extraction five studies were excluded (i.e., Knowler et al. 2019; Marchant et al. 2019; Reynolds 2021; Rivers 2016). These studies were excluded because, upon further inspection during data extraction, it was concluded that they did not meet our inclusion criteria. A brief description of these programmes is provided in Appendix S1G.

The primary reason for excluding these process evaluations was that the findings did not sufficiently address student behaviour, which could lead to a child being excluded from school. Three evaluations (i.e., Marchant et al. 2019; Rivers 2016) were excluded at the data extraction phase for this reason, as during coding of findings, it was apparent that the findings related to general classroom behaviour rather than disruptive or misbehaviour that we would consider indicative of increased likelihood for exclusion. The evaluations by Knowler et al. (2019) and Reynolds (2021) were excluded because the intervention setting was not mainstream schools, and as such did not meet our inclusion criteria.

### 7.2 Qualitative Evidence Synthesis

#### 7.2.1 Overview of Thematic Framework

In our synthesis of process evaluations for this review, we were mostly interested in exploring findings related to what participants perceived as facilitators to implementation and/or barriers to implementation. We applied a thematic framework, grouping findings from individual process evaluations into themes and subthemes and categorising them as either a facilitator or a barrier.

The main themes were: (1) Intervention format; (2) (in)Consistency; (3) Buy‐in; and (4) Seeing results. Multiple subthemes were identified for each of these themes, and many subthemes related to both facilitators and barriers. These themes and the corresponding subthemes are outlined in the following sections alongside accompanying extracts and quotations from process evaluations and participants themselves as evidentiary support.

#### 7.2.2 Main Theme: Intervention Format

Many process evaluations reported that the intervention format was a significant facilitator, but aspects of the intervention format were also noted as a barrier to implementation. By intervention format in this context, we refer to any aspect of the composition of the intervention programme implemented, for example, the inclusion of group activities, implementation of teacher training, or the materials used to implement the intervention.

In addition to the subthemes relating to intervention format which are outlined in the following sections, there were some general overarching themes that emerged in our qualitative evidence synthesis. Notably, a significant facilitator to implementation was that teachers, school staff, and students viewed the intervention itself quite positively and reported enjoying the activities.

Students were found to have enjoyed intervention programmes in evaluations by Blandford‐Elliott (2021) and Humphrey et al. (2018). However, few evaluations interviewed students directly about their perception of the intervention. In an evaluation of the GBG, Humphrey et al. (2018) report that students enjoyed the intervention activities and said:

…it was the best game, learning game, in the world.

(Student; Humphrey et al. 2018, 54)

In an evaluation of the Learning Together programme, students enjoyed their involvement in the intervention activities and involving the student voice was perceived as a facilitator to implementation. For example, as one member of staff reported:

I think that the students will certainly enjoy the fact that we're doing something like this so they can be involved in it and that they can actually have their voice heard, that they can feel safe at school, that they can feel they're listened to.

(Staff member; Warren et al. 2020, 7)

Participants in a process evaluation of SWPBIS appreciated the involvement of researchers and felt they benefited from the ability to ‘ask questions and gain a deeper understanding of the rationale for the systems’ (Blandford‐Elliott 2021, 68). Other participants commented positively on the evidence‐based nature of the intervention programme. For example, one headteacher noted:

So when I met [researcher's supervisor] and you, and when you opened up the world of research to say, “Look, this works and this is why it works,” and the evidence that backed it up, it just gave that strength of conviction that, A, what I believe is true and B, here it is. Here it is, that's worked. And C, here it is in action, it's fascinating. Absolutely brilliant.

(Headteacher; Blandford‐Elliott 2021, 67)

Similarly, the evidence base supporting the impact of SWPBIS was received positively by teachers interviewed by Blandford‐Elliott (2021). For example, one teacher commented that ‘…knowing it had been used in other classrooms or whatever … and that it wasn't just a theoretical thing based on somebody who's written a book somewhere about their opinions’ (Blandford‐Elliott 2021, 68) was a positive aspect of the intervention.

In the same vein, teachers interviewed by Allen et al. (2020) in an evaluation of the Incredible Years Teacher classroom management programme appreciated the aspects of the programme that explained the theoretical framework of the intervention. Allen et al. (2020) identified that this appreciation motivated teachers to implement the intervention. For example, one teacher commented:

…really understanding where those strategies come from as opposed to just going “Oh yeah we'll give you stickers because that's nice” … It's really going through why you're saying what you're saying and what effect that that's having so it's much more thought through.

(Teacher; Allen et al. 2020, 1167)

These theoretical frameworks and evidence‐based were generally presented to teachers in designated training sessions. Middleton (2022, 79) report that participants perceived training sessions positively, and a significant facilitator was that the content ‘complemented existing knowledge, provided additional knowledge and increased the confidence of staff to meet the needs of pupils’. Moreover, having experts with both intervention knowledge and previous teaching experience conduct the training was also perceived positively (Middleton 2022).

However, teachers interviewed by Humphrey et al. (2018, 58) did not appreciate the theoretical focus of training sessions, and would have preferred practical, ‘streamlined’ and shorter training sessions. Likewise, training content in the ACE‐Informed Schools intervention that was seen as

…quite heavily scientific was a barrier for some participants, and head teachers commented that staff would prefer ‘more practical things’.

(Head teacher; Sparling et al. 2022, 45)

Teachers interviewed by Stanbridge and Campbell (2016) appreciated the early intervention approach and the fact that the purpose of the intervention was to understand and address the reasons for misbehaviour, instead of simply reacting to the behaviour. As one teacher commented:

I think it's just really, really thinking about what that behaviour means and why it's happening. Rather than just addressing it like we tend to do … [] rather than just addressing the behaviour on the spot, you're actually looking into why it's happening. And I think that's the key thing.

(Teacher; Stanbridge and Campbell 2016, 273)

This approach was a fundamental element of the intervention evaluated, and it was well received by participants. Others noted that the targeted children were those who were not quite eligible for Child and Adolescent Mental Health services (CAMHs) at the time of the intervention, but that without it, they may end up needing these services. Indeed, staff identified that by intervening early, instead of letting problems become ‘embedded’ (Stanbridge and Campbell 2016, 276), the outcomes and trajectory of these students can be improved. This may include the risk of being excluded from school.

…be school failures, you know, they're gonna be the people that don't leave um … education with the qualifications they need to do what they want in life. So it's about getting in there early for children that, that just, just don't reach that high threshold.

(School staff; Stanbridge and Campbell 2016, 276)

Similarly, in the ACE‐Informed Schools model, training facilitated a ‘mindset change’ among teachers and staff to understand the causes and explanations for misbehaviour better (Sparling et al. 2022). This led to teachers adopting a more nurturing and empathetic approach to students who have experienced adversity, and as Sparling et al. (2022) outline, this led to improved teaching and also more inclusive schools.

Overall, although the format and type of interventions varied between included process evaluations, participants generally viewed the intervention format positively; however, there were some notable barriers and limitations. The following sections describe the facilitators and barriers with respect to the sub‐themes related to intervention format in more detail.

##### 7.2.2.1 Subtheme Facilitator: Group Discussion and Collaboration

Group discussion was identified by Middleton (2022) as a key facilitator in the nurtureUK VRU programmes to reduce school exclusions. For example, one participant noted:

It's prevented two boys from permanent exclusion … The staff all looked at the six principles to decide what were our strengths and weaknesses. It's allowed for staff discussion around nurture and attachment theory.

(Participant; Middleton 2022, 77)

Collaboration was a key component in the group format that facilitated the implementation of interventions. The benefits of collaboration were mentioned by many participants that took part in many process evaluations, especially in instances where the programme incorporated a group element. This was often because collaboration was an integral part of the intervention, for example, through group work or whole‐school meetings. The group format aided in the impact of the intervention and also made teachers feel supported.

Furthermore, having opportunities for school staff to work collaboratively to address the needs of children whose behaviour is causing concern can promote a solution‐ and outcome‐orientated approach … As well as a sense of peer support.

(Researcher; Stanbridge and Campbell 2016, 275)

Students, too, were found to enjoy group work when interviewed as part of multiple process evaluations (e.g., Humphrey et al. 2018; Warren et al. 2020). In the GBG, students enjoyed working with their peers, as one commented:

[I] like it when you work in partners.

(Student; Humphrey et al. 2018, 54)

In an evaluation of the Learning Together intervention, students enjoyed being members of the intervention action group with teachers and members of the school staff. A key facilitator here was, similar to teachers, students appreciated getting others' perspectives and different opinions:

I think mainly just having other people's, seeing other people's views and seeing how … if we had the same views or … hearing someone else's point of view and thinking, “Oh yeah.”

(Student; Warren et al. 2020, 7)

Interestingly, Warren et al. (2020, 8) note how the collaboration and open discussion between students and teachers led to an increase in positive relationships and that students hearing from staff about the impact that disruptive behaviour from just one student was a key facilitator. Working together and student–teacher collaboration in this intervention led to increased respect, something students viewed favourably:

…much more respectful … yeah, they treat you with the same amount of respect as they would do their colleagues.

(Student; Warren et al. 2020, 8)

Participants, particularly teachers, noted that the opportunity to collaborate and work with individuals outside of their own school was a particularly desirable aspect of the intervention: ‘I've enjoyed working collaboratively with other schools and with outside agencies’ (Teacher; Blandford‐Elliott 2021, 69). Even teachers from schools with low fidelity of implementation commented positively about this element of collaboration when interviewed as part of the process evaluation of SWPBIS. For example, one teacher noted:

…it was really nice to have the meetings that we had. It was really nice to come together and listen to what other people were doing and you know, how it was working. I found it really helpful, and I think that was a really good aspect of the project that we were allowed time to meet and it was useful to hear what other schools were doing and what worked and possibly what didn't. I really valued that time.

(Teacher; Blandford‐Elliott 2021, 69)

This allowed teachers to consider different perspectives and gain valuable insights from their peers from diverse backgrounds in other settings who were also taking part in the intervention (Allen et al. 2020). Teachers in particular viewed hearing from other participants about their struggles or challenges was a positive aspect of collaboration and the group format. The idea that they were not alone in finding particular aspects difficult, or that they were not the only school struggling with student behaviour was viewed quite positively.

It was nice to hear the other stories from other schools … about what was working, what wasn't working.

(Teacher; Humphrey et al. 2018, 58)

This was particularly true when groups were non‐judgemental and supportive. For example, one teacher commented:

For me it was like therapy for teachers […] having the time to come away from the classroom and realise that those little things that really bug you on a day to day basis everyone feels the same and it's ok to get you know, to feel at times frustrated […] just be reminded of the strategies and ways to deal with it, and that it's OK, was really powerful for me and I went back to school each time for that next sort of few weeks feeling really great.

(Teacher; Allen et al. 2020, 1165)

Those implementing the intervention also noted that group meetings served a practical purpose. One facilitator noted that when meetings were forthcoming, it served as a reminder to check the progress of the implementation and monitor the progress in their school (Blandford‐Elliott 2021).

One process evaluation involved an intervention that was delivered to groups of students who were considered high‐risk for behavioural problems that may lead to suspension or exclusion. In this case, a key facilitator to implementation was the informal and confidential nature of student groups. Participants felt that this allowed students to ‘open up’ and discuss issues freely, without the fear of judgement, thereby being a facilitator to the purpose of the intervention (Wright 2020).

The informal nature of these groups was also important to students, with many teachers noting that through sharing their own stories and perspectives and removing the ‘student–teacher’ formality, students were able to engage better with the intervention.

I think they are seeing me as the year's gone on as a more real person to them [mmhmm] ya know and not somebody that's a little bit colder or more in place as a teacher is.

(Teacher; Wright 2020, 253)

I was telling em real life stories of their friends and them themselves, me as a mum and how I've viewed risk and … children that I've known in the school doing really risk things of year eight and they really engaged with that.

(Teacher; Wright 2020, 262)

Therefore, as the findings from included process evaluations demonstrate, participants in school‐based interventions appreciated the opportunity to work in groups, particularly when these groups included other participants from other schools. Collaborating with others was a facilitator to intervention implementation, as participation in group sessions often offered support and encouragement to teachers. Students, too, benefited from group discussion and collaboration, particularly when these groups were more informal in nature. We consider group discussion and collaboration as a facilitator to the implementation of the intervention, as it seemed to increase participants' interaction and engagement with intervention activities. It is a simple aspect of the programme, but it could substantially impact several aspects of implementation adherence and the effect on students.

##### 7.2.2.2 Subtheme Facilitator: Group Leaders

Group formats in included interventions worked particularly well when group leaders were viewed positively. Many participants noted that supportive, non‐judgemental, and informal relationships with the group leaders were positive aspects of included intervention programmes (e.g., Humphrey et al. 2018; Middleton 2022).

Staff felt they did not need direct support but were comforted that they knew it could be accessed as required.

(Researcher; Humphrey et al. 2018, 62)

[The coach is] really positive with the feedback that she gives, she sends quite detailed reports back through … she always offers you that next step with where to go next.

(Teacher; Humphrey et al. 2018, 59)

Participants who experienced the nurtureuk VRU programmes, which specifically aimed to reduce school exclusions, appreciated leaders' enthusiasm and belief in the intervention, and also the supportive guidance provided:

[We] couldn't be without the [LC] – she's instrumental! You can tell she's doing it because she believes in it – she's amazing!

[The LC] has listened intently to our ideas and projects and has provided us with support and guidance.

(Participants; Middleton 2022, 77)

In interventions that involved group sessions, the role of group leaders was a fundamental facilitator. On one hand, patronising or inflexible group leaders who were not ‘open to new ideas’ were not successful, but Allen et al. (2020) noted that group leaders who were seen as welcoming, friendly, open, and supportive were perceived well by teachers. One teacher reported:

There was a lot of humour but also they didn't ever make you, they didn't put you in a position where you felt awkward or uncomfortable, you were invited to share but not forced to and I think that is quite important.

(Teacher; Allen et al. 2020, 1166)

Regular meetings with group leaders were also a notable facilitator in interventions to reduce children being excluded from school (Middleton 2022). Meetings occurred both in‐person and virtually, but the regularity allowed participants to develop effective partnerships, generate ideas and work with the lead consultant on practical implementation steps (Middleton 2022, 78). Group leaders in this intervention were not only seen as supportive, but they were also a source of ideas and practical advice:

Gave us lots of ideas; whatever is asked gets delivered and more! [They] put things into intelligent, practical terms.

(Participant; Middleton 2022, 78)

Overall, group leaders were viewed positively by participants in the included process evaluations. We identified that, as an aspect of the intervention format, supportive and non‐judgmental group leaders were a considerable facilitator to implementation. Similarly, regular meetings with group leaders were a facilitator, especially in an intervention to reduce school exclusions, as participants appreciated regular and practical support with implementation (e.g., Middleton 2022).

##### 7.2.2.3 Subtheme Facilitator: Flexibility

A further aspect of the intervention format that was perceived positively by participants was the flexible nature of the intervention. For example, participants noted that the flexibility of the intervention being at the core of the theory of change was a significant facilitator. Teachers commented that they liked that they could adapt, change, or modify aspects to suit the needs of their students, and they also appreciated that this was indeed the purpose of the intervention materials in many cases. This is perhaps particularly important when considering school‐based interventions, and even more so with school‐based interventions to address difficult behavioural or disciplinary issues such as school exclusions. As Allen et al. (2020, 1167) note, ‘no one strategy will work for everyone or forever’.

Some teachers adapted specific terminology in the intervention (GBG) to increase specificity. One of the four rules in the GBG is to have students remain seated, but some teachers modified the terminology to make the rule clearer to students. For example, one teacher changed the phrasing to ‘TNT – tummies near tables’, another used ‘BBC – bums and backs on chairs’ or ‘six legs on the floor’ (Teachers; Humphrey et al. 2018, 53). This flexibility is ingrained into the intervention and one teacher who had begun implementing the rules very literally was told by their intervention coach: ‘do what makes sense to the kids that you've got in front of you.’ (Teacher; Humphrey et al. 2018, 53).

Participants could also be flexible in adjusting the format of particularly favourable aspects of the intervention. For example, action groups with both students and teachers were a key facilitator in the Learning Together intervention, as previously discussed, but teachers reported adjusting the arrangement of these groups to further the positive impacts:

What I've tried to do is tried to be able to break away into smaller groups as often as we can in those meetings so that they can be able to have a one‐on‐one with the members of staff. And as soon as that happens … that's when really the conversations start in there.

(Staff; Warren et al. 2020, 8)

Flexibility was especially important in interventions where there was a possible stigma attached to participation for students. Wright (2020) interviewed participants who had taken part in an evaluation for high‐risk students, and participants commented that, as such, there could be a stigma for students who take part. In such instances, the flexibility and ability to adapt materials were seen as a facilitator to implementation. As one teacher commented:

I liked the templates for the letters erm I did make a few adaptations I just wanted to word something a little bit differently … we worded it more that we just feel that at this age it's worth going through this course … just so that parents didn't automatically feel that your child's been chosen … we wanted to make it sound a little bit more like any child could get chosen.

(Teacher; Wright 2020, 268)

Others noted that the flexible structure of the intervention meant that it could be implemented throughout the school day, which was a significant facilitator (e.g., Humphrey et al. 2018; Wright 2020). In an evaluation of the GBG, teachers appreciated the flexibility to decide, based on their specific classrooms, which lessons were best suited to the intervention. For example, while one teacher said:

…started using it within my English lessons … more difficult in other subjects, for instance, Maths, because sometimes you need the children to talk to each other.

(Teacher; Humphrey et al. 2018, 54)

Another noted:

[GBG] works really well in Maths because we can use it for a lot of starter activities … it doesn't work well in English because they need a lot of support.

(Teacher; Humphrey et al. 2018, 54)

Thus, the flexibility to decide when and in what contexts to implement intervention activities was a facilitator to implementation for these teachers. Moreover, some teachers found that ‘transition’ points during the school day (e.g., following lunch or breaks) were especially well suited to the GBG intervention, and that this flexibility in the timing of implementation throughout the school day was a facilitator.

…a really constructive way to get them refocused in the afternoon and ready.

(Teacher; Humphrey et al. 2018, 54)

When there was a lack of flexibility, this was identified as a barrier to implementation. For example, when speaking about the required lack of interaction between teachers and students in the GBG, one teacher said:

I don't like having rigidities that you can't give a little bit of support … and some of our children do need support when the game's on … they do need that little bit of interaction from adults because sometimes they're not able themselves to express how they're feeling…

(Teacher; Ashworth 2018, 219)

Overall, flexible intervention formats were seen as a facilitator not only to implementation during an evaluation, but also to continued implementation and integration of new strategies and approaches into daily practice. Teachers appreciate the ability to adjust materials, intervention content/messaging, and timing of implementation to best suit their needs and the needs and abilities of the students they teach.

##### 7.2.2.4 Subtheme Facilitator: Structure

Above and beyond the flexible structure and adaptability of interventions, the intervention implementing a structure was also perceived as a facilitator, particularly for children with special educational needs. These groups appreciated the structure implemented during the GBG and the clear rules involved. One teacher noted:

There's a lot of children that thrive on structure and because it gives … such a basis of structure … it's worked really well… Yeah again the structure really helps them so I've got … eleven children on the SEN register in my class but … just because the rules are so explicit and because it like the directions are so explicit they just really thrive under it and know exactly what to do and like the … team work element's been massive for them.

(Teacher; Ashworth 2018, 221)

In particular, when the intervention fit the school context and addressed the school's vision, the structure provided by interventions facilitated long‐term support (e.g., Middleton 2022; Sparling et al. 2022):

The philosophy and principles of [the programme] meet the needs of our school context and has led to a review of how we approach inclusion and supporting all learners, but especially our most vulnerable.

(Participant; Middleton 2022, 77)

Teachers also found that the structure of the intervention, being centred around planning, was a facilitator to implementation. Stanbridge and Campbell (2016) found that participants appreciated not only the actual plan itself, but the process of creating a plan. This time was useful to further their understanding of student behaviour and the necessary response. Another key facilitator was that the intervention allowed participants to consider the whole child, instead of addressing a few key issues.

Not only was having a plan a positive aspect of the intervention, teachers also reported that the process of creating a plan was beneficial.

(Stanbridge and Campbell 2016, 273)

I think it's good, it's a pretty comprehensive format actually, I think. I think it's really well done, and it focuses on the child, and the holistic child rather than just bits of the child. It's the whole child, not just the behaviour. It helps you to focus on the child, rather than he's a naughty boy.

(Lead Behaviour Coordinator; Stanbridge and Campbell 2016, 275)

However, as previously outlined, flexibility and the opportunity to modify the dosage of the intervention were also desirable factors for participants interviewed by Stanbridge and Campbell (2016). For example, one lead behaviour coordinator noted:

And sometimes when children have got very very difficult, to give them more and more and more time to dwell on that, but in a non‐power position, from the victim, hinders their progress. You know, yes they need time to talk, but then they need time to draw the lid, leave and get on with what they're really here for.

(Lead Behaviour Coordinator; Stanbridge and Campbell 2016, 275).

Participants also found that having designated time away from teaching and normal school activities was a positive element of interventions. Allen et al. (2020, 1166) found that time away from the day‐to‐day stresses and demands of the classroom to reflect on practice was pivotal in the success of the intervention to change and improve their skills in classroom management. In a practical sense, too, teachers interviewed by Allen et al. (2020) found that the structure and intensity of the intervention were desirable. The Incredible Years classroom management programme was implemented for 6 months, and teachers attended training sessions (in groups of 8–12 participants) 1 day per month. Teachers noted that this duration allowed them to evolve and deepen their understanding of the intervention and also grow relationships within their groups (Allen et al. 2020).

In general, across process evaluations, interventions that provided structure and involved creating a plan to address student behaviour were viewed positively. Flexible structures were particularly favourable to teachers.

##### 7.2.2.5 Subtheme Barrier: Materials

The overall view across process evaluations was that intervention materials were often a barrier to implementation. In this context, when referring to materials, we are describing the actual intervention content and how it was delivered. Included within our conceptualisation of materials thus, are elements of interventions such as student worksheets or booklets, videos used in training sessions, or any visual representations used to explain concepts included in the intervention.

For example, some participants commented that intervention materials were costly, both in terms of the financial investment and time‐consuming nature (e.g., Allen et al. 2020). Others felt that the materials were not suitable for their students or their classrooms. Materials were often viewed as inappropriate for older groups of students. As one teacher noted:

The only thing I haven't really used is the like the commentary … I think as they're a little bit older it's not, I don't feel its was as effective […] I don't think it's quite worked as well as it would if they were younger children.

(Teacher; Allen et al. 2020, 1168)

It was also an issue when participants perceived materials as being outdated. This was seen as a barrier to implementation. For example, participants interviewed by Wright (2020) took part in an intervention whereby behaviour and risk factors for behaviour were explained using a turning cog chart. Teachers felt that this approach was outdated, noting that some students in modern times may never have seen cogs.

Well I had a think it was what leads on from what maybe a flow chart [ok] would be better [yeah] I think that the erm the mechanics of a turning cog I'm not sure they've ever seen turning cogs.

(Teacher; Wright 2020, 258)

Video materials in training sessions were also often not perceived well by teachers. Some felt the scenarios depicted in videos (e.g., of a teacher observing a class of students engaged in an activity in silence) were unrealistic. As one teacher said: ‘I was thinking “you can't do that in the middle of Maths.”’ (Teacher; Humphrey et al. 2018, 57). Including old and outdated video footage was also noted as a barrier to implementation, as it impacted the relatability of the content.

Other elements of the programme that were mentioned as those that students did not like were … the videos related to peer pressure, which were felt to include footage that was too old and out‐of‐date and therefore not relatable.

(Researcher; Wright 2020, 258)

We interpreted this as a barrier to implementation, as participants are less likely to engage or show enthusiasm for materials that they do not view in a positive light. A lack of enthusiasm from teachers or other staff responsible for using these materials to deliver the intervention is likely to hinder student engagement, too. Some of the intervention materials were deemed to be unsuitable for the intended participants, as teachers interviewed by Wright (2020) noted:

…there was an awful lot of writing expected [ok] so erm I tried to sort of turn it into a more of a conversation and just write down a word…. they don't really wanna be writing a lot down and ya know that if they were in class they would be the the class that doesn't write a lot down.

(Teacher; Wright 2020, 256)

Some teachers also noted that some of the training content was too simplistic, but that eventually they understood the purpose of including basic and introductory level materials:

The first few sessions I felt were kind of like behaviour management 101 and [I] thought well, been doing this for many moons and I thought a lot of it was very, very basic […] Looking at it at the end, overall I can understand why there was an emphasis on that bit because it all sort of falls into perspective as to what is most important.

(Teacher; Allen et al. 2020, 1166)

Similarly, participants also noted that they had difficulties with the physical, or ‘hardcopy’, versions of intervention materials. For example, teachers generally perceived the Incredible Years manual favourably; some commented that they would have preferred an electronic copy (Allen et al. 2020). Other physical intervention materials, such as the booklets in the GBG, were confusing to children and were not received well. As teachers interviewed by Humphrey et al. (2018) commented: ‘they hate the books […] they disrupt the flow and … it's very confusing for the kids – they just stamp in random places.’ (Teacher; Humphrey et al. 2018, 58).

Despite this general dislike of intervention materials, some participants did find positives in the materials used to implement certain programmes. For example, participants in one intervention (SWPBS) noted that, when additional materials were required to implement intervention activities, the fact that these could be acquired at a relatively low cost was a positive.

…the low cost element of it, because the minute someone comes back with an idea that is gonna cost money, it doesn't happen

(Teaching assistant; Blandford‐Elliott 2021, 70)

In addition, participants who took part in the Incredible Years classroom management programme received a copy of the intervention manual. Teachers commented that this was helpful to ‘dip in and out of’ a useful reference to have when discussing ideas with their colleagues or parents (Allen et al. 2020).

Some participants in some included interventions noted that the intervention materials could be easily used in other classes, and this was considered a facilitator to the implementation of the intervention (e.g., Humphrey et al. 2018). It follows that a facilitator for implementing interventions to reduce school exclusions or suspensions is to involve intervention materials that work across all contexts and throughout the school day.

Overall, intervention materials were often cited by teachers as barriers to implementation and issues with generalisability and transferability, across contexts, age groups and abilities often impacted the delivery of intervention materials. This was seen in relation to materials used to deliver the intervention to students (e.g., Humphrey et al. 2018; Wright 2020) and materials used to train teachers (e.g., Allen et al. 2020).

##### 7.2.2.6 Subtheme Barrier: Transferability

In the same vein, many participants in included interventions found that a significant barrier to implementation was the transferability of the programme. Many noted that the interventions were ‘Americanised’ and as such were not suitable in UK classrooms. For example, in a teacher training intervention, video content was not applicable to a UK context due to the size of the classrooms depicted (i.e., classrooms were larger than the typical UK primary school; Allen et al. 2020). Others could identify when an intervention had not been designed for the UK education system, commenting:

…maybe the people that designed it hadn't designed it with necessarily … [with] the British curriculum in mind, there's too many things for us to have to cram into a year.

(Teacher, exit interview; Humphrey et al. 2018, 42)

Others noted that the interventions were not transferable across different age groups that they teach. Applicability to different age groups was a common barrier mentioned throughout process evaluations. Some participants noted that certain aspects of intervention programmes were seen as ‘immature’ or too young for older students. This is important, as it is reasonable to assume that students would be more likely to disengage from an intervention that they perceive as being condescending, immature, or childish. For example, in relation to a ‘Traffic Light system’ used in the Risk Avert intervention, Wright (2020, 258) noted: ‘…the traffic lights, which students felt were too boring and/or too immature for them.’

Transferability based on student ability was also a notable barrier. In evaluations of the GBG, evaluations found that there were differential effects based on student ability, with higher ability children becoming bored with the activities and lower ability children ‘switching off’ and relying too heavily on their peers to engage (Humphrey et al. 2018, 55).

Initially I played that game rigidly, but it became demotivating for the really low achieving students who, as much as I wanted to encourage to work independently, and that’ exactly what I wanted to do, some just couldn't access it.

(Teacher; Ashworth 2018, 212)

Specific aspects of the programme, such as the lack of interaction and student‐led activities, were particularly difficult for students with special educational needs or lower ability (Ashworth 2018). Not only did this lack of interaction affect the impact of the intervention for these students, but it also reduced teacher enthusiasm and enjoyment of the activities. For example:

Very low children are just used to an adult being with them all the time and encouraging the, or … boosting their confidence or even just kind of modelling through what they need to do, and I find it really difficult to do an independent numeracy task because they're just not used to that independent learning.

(Teacher; Ashworth 2018, 212)

As such, when interventions were identified as not being a ‘good fit’ for UK classrooms or involved activities that were not suitable for all students in a class, this was a considerable barrier. Transferability across different age groups was also a common theme. This may relate to the fact that interventions were developed in the United States, where the structure of the education system is different to that in the United Kingdom. For example, in the United States, an intervention may be designed to be implemented in middle schools, as these are separate educational institutions from high schools. Thus, when transferred to the United Kingdom, where schools will typically include those eligible for middle and high school in the same system, there may be issues with transferability.

##### 7.2.2.7 Subtheme Barrier: Timing

The timing of the intervention was also identified as a barrier to implementation by many participants across multiple process evaluations. For example, school staff who were interviewed in the evaluation by Stanbridge and Campbell (2016) noted how the ‘relatively short length of the intervention period, the timing (summer terms and Christmas), and the time pressures on staff within the school’ were notable barriers to the impact of the intervention (Stanbridge and Campbell 2016, 272).

Similarly, teachers interviewed by Allen et al. (2020) felt that implementing an intervention from November to April was not effective, as they would have preferred to be able to implement strategies from the beginning of the academic year. Participants who experienced the nurtureuk VRU programmes between 2020 and 2022 were inevitably impacted by the COVID‐19 pandemic and the lockdown of UK schools. This was perceived as a barrier to implementation due to practical reasons. However, participants did note that the move to online remote teaching led to increased parental involvement (Middleton 2022). Aspects of the intervention were identified by participants as helpful in structuring conversations with parents and improving those relationships. For example:

The Boxall Profile helps to structure conversations with parents and ‘Parents are more forthcoming and it's helped break down barriers. It's changed a lot of parents' attitudes.

(Participants; Middleton 2022, 78)

Time was also an important barrier when the intervention was perceived as being too time‐intensive, and participants struggled to implement it alongside their regular busy schedules. This led teachers not to want to continue with implementation, as Humphrey et al. (2018) found:

…that's time out of the curriculum and these days schools can be quite sensitive about that.

(SENCO teacher, exit interview; Humphrey et al. 2018, 42)

It took too long to implement based on the beginning parts and the end parts.

(Teacher, exit interview; Humphrey et al. 2018, 42)

Time to implement interventions is a key component to its success, but often participants felt they did not have sufficient time available. Sparling et al. (2022) report that some teams just did not have the capacity to implement the intervention on a wider scale. Indeed, for some participants in the nurtureuk VRU programmes, a lack of time was the only significant barrier to implementation. For example,

The only major obstacle has been time to implement the project.

(Participant; Middleton 2022, 80)

Some participants noted that they struggled to get leadership to give the time needed for implementation: ‘Getting leadership to give time [due to] lack of funds [for] staff training and the resistant teachers.’ (Participant; Middleton 2022, 81). Similarly, practical issues are cited by Sparling et al. (2022) as a key barrier to implementation. Often, training and key aspects of the intervention occurred during teaching hours and finding staff to cover teaching responsibilities and financial support was difficult and a barrier to participation (Sparling et al. 2022).

Unsurprisingly, timing and a lack of time to implement interventions was a common barrier identified by teachers who took part in evaluations of school‐based interventions. Availability of teachers and staff to implement interventions is a valuable resource required to implement interventions in schools, but this is limited, as there are several competing demands. Over and above teachers' time, the timing of implementation was also a barrier, with some interventions impacted by school lockdowns due to the COVID‐19 pandemic and others identifying that implementing interventions during busy terms is difficult.

#### 7.2.3 (In)Consistency

Consistency was a recurring theme identified across the included process evaluations. Many participants noted that consistency of implementation and intervention policies was a notable facilitator. However, in contrast, participants also found that inconsistency of leadership (e.g., staff turnover), implementation across the whole school, and between new and old policies, was a significant barrier. The following sections will explain the theme of (in)consistency in further detail.

##### 7.2.3.1 Subtheme Facilitator: Consistent Policies

Overall, many participants in multiple process evaluations found that their schools' existing behaviour policies were not consistent, and they appreciated that the intervention led to the creation and implementation of consistent policies. This gave them clarity. As one teacher commented:

…every child and member of staff knows exactly what the expectations are. They all know what to do in a certain situation.

(Headteacher; Blandford‐Elliott 2021, 63)

Teachers labelled previous policies as ‘reactive approaches to risk behaviour’, which led to inconsistencies (Wright 2020, 233), and the new policies implemented as part of the intervention were more proactive and consistent. Therefore, while new policies were identified as clashing or being inconsistent with those previously in place, teachers often favoured the change. For example, interventions allowed teachers to adopt additional steps into their schools' approach to student misbehaviour:

I'm still following my behaviour policy but I've put so much more in place that I don't hit the first step of it yet […] so for instance our [school policy] is name of the board, three dots, headteacher […] but I'm getting to the point now where I'm putting in so much in before that I'm not needing to put a name of the board […] because that's personally something I don't agree with.

(Teacher; Allen et al. 2020, 1170)

In a process evaluation of SWPBIS, teachers noted that the new behaviour policies implemented as part of the intervention were more consistent than the policies schools already had in place. This increase in consistency of policies was identified as having a positive impact on student behaviour and increased staff buy‐in. One teacher noted:

[Talking about the behaviour policy] It wasn't clear, it wasn't consistent. I'd been there four years and I still didn't know which types of behaviour I should be dealing with within a classroom setting … it's nice to know that we've got this plan that we're all on the same page with everything. So I think that's had a really good impact on the school and on the behaviour in class and, yeah, I think that was great and helped a lot of staff get on board.

(Teacher; Blandford‐Elliott 2021, 64)

Inconsistency with previous policies was also identified in a process evaluation of the GBG (Humphrey et al. 2018). One teacher commented that their school's previous policy relied heavily on sanctions for poor behaviour, whereas the intervention implemented strategies they preferred as it allowed ‘children [] make a mistake and learn from it’ (Teacher; Humphrey et al. 2018, 55).

Overall, participants in included process evaluations noted that schools' existing behaviour policies were inconsistent, and that new approaches and strategies introduced during intervention programmes were more consistent. This was seen as an improvement and was a facilitator not only to implementation during the evaluation, but also to continued implementation and use of intervention policies after the evaluation had ended.

##### 7.2.3.2 Subtheme Facilitator: Whole‐School Approach

Consistency of the intervention implementation across the whole‐school community and different settings was also a significant facilitator in many evaluations. Creating a school environment where all members of staff were adopting the same approach and following clearly outlined rules and regulations was noted as having a positive impact on students. Similarly, ensuring that the intervention message was consistent and visible across all aspects of the physical school environment was seen as a facilitator.

…I think for the children it's important to have the continuity, and that every adult that they see in school is saying the same kind of thing … so if they're in the corridor or if they're in the classroom or anywhere like that, that they've got the same message coming

(Teacher/Deputy Headteacher; Blandford‐Elliott 2021, 63)

Outside of the formal intervention implementation, this consistency between members of staff was something that teachers wanted to continue. Thirteen participants interviewed by Blandford‐Elliott (2021) noted that consistency of the approach was a facilitator and that consistency across all settings was something they wanted to continue. For example, as one member of a senior leadership team noted:

More consistency. Cause now we've got this student behaviour management process that's in every classroom, it's up in the hall. You know, it's everywhere the children go. So all staff, now, are singing off the same hymn sheet … everybody should be treated equally and fairly throughout the school, and I want that to continue.

(Teacher/Senior Leadership Team; Blandford‐Elliott 2021, 64)

##### 7.2.3.3 Subtheme Barrier: Lack of a Whole‐School Approach

While some interventions evaluated by included process evaluations involved a whole‐school approach (e.g., SWPBIS, Blandford‐Elliott 2021), this is not true across all included process evaluations. Furthermore, where an intervention did not use a whole‐school approach, this was identified as a barrier to implementation as it created inconsistency across members of staff. For example, one teacher who participated in the Incredible Years classroom management programme commented:

You need everybody on board really don't you because if you're going to be consistent with your behavioural management you need not just you to be doing, you need the other adults that you work with to be doing the same thing.

(Teacher; Allen et al. 2020, 1169)

A core aspect of this intervention was an approach to ‘low‐level poor behaviour’ whereby teachers are instructed to ignore and not react to misbehaviour by students. However, when other staff (e.g., teaching assistants) did not understand, accept, or support this approach, they may ‘step in’ and undermine what the teacher was trying to achieve (Allen et al. 2020). Some teachers even noted that this could have a detrimental impact on student behaviour:

They don't understand that they just can't be like that with particular children because it does not get the best out of them […] it's just going to make them more angry and they're going to end up doing something else wrong which will get into a spiral of negativity.

(Teacher; Allen et al. 2020, 1169)

Thus, consistency between members of staff at the classroom level is vital to the successful implementation of this intervention. Participants in this process evaluation also commented that they struggled with this practice of ignoring some behaviours, and so having a whole‐school approach and support from their colleagues may be even more important. These approaches were mentioned in relation to OFSTED evaluations and observations, and teachers highlighted the need to justify practices that could be interpreted as poor practice (Allen et al. 2020).

Inconsistency at the school‐level between members of staff was also an important finding that emerged from our qualitative evidence synthesis. Teachers interviewed by Allen et al. (2020) really advocated for a whole‐school approach and recommended that lunchtime assistants were also trained and incorporated into the Incredible Years programme. Moreover, Warren et al. (2020) found that specific aspects of the intervention (e.g., RP) were not implemented consistently enough across members of staff in order for the change to become regular practice. This did not occur across all schools that took part, but was a specific barrier in one school where there was less need for the intervention.

##### 7.2.3.4 Subtheme Barrier: Staff Turnover

Inconsistency in staff and high levels of staff turnover were also barriers to the implementation of interventions. Often, when intervention programmes, like the GBG, were perceived as being time‐intensive and demanding, inconsistency among the staff members was a challenge to implementation (Humphrey et al. 2018). Schools have many competing priorities, and as such, implementing a programme was difficult under normal circumstances, but harder with high staff turnover. As one head teacher noted:

It is proving increasingly difficult to maintain the expectations for training and visits. I am losing another member of staff this term to be replaced possibly by another NQT … the team needs to be allowed to work on … getting it right for the children. They are finding it difficult to meet the pressures of teaching and find the GBG [Good Behaviour Game] is placing more strain on them as teachers and on the school in general, finding available staff to cover for training and meeting purposes, etc.

(Head teacher; Humphrey et al. 2018, 44)

External pressures and changes in staff and leadership was also a key barrier for participants interviewed by Sparling et al. (2022). Turnover led to the intervention not being implemented in a consistent manner, a notable barrier to implementation and impact.

#### 7.2.4 Buy‐in

Buy‐in was an interesting theme that emerged from evidence across the included process evaluations. When referring to ‘buy‐in’, we are discussing the concept of participants engaging in, accepting, and actively supporting an intervention programme. Generally, a good degree of buy‐in from members of the school community was a facilitator to implementation, and a lack of buy‐in was a barrier. The following sections discuss these subthemes related to good ‘buy‐in’ and a lack of ‘buy‐in’ in further detail.

##### 7.2.4.1 Subtheme Facilitator: Good ‘Buy‐in’

Overall, when staff and school leaders were seen to buy into an intervention, this was mentioned as a significant facilitator to implementation. Buy‐in was highly related to seeing results and seeing the beneficial impact of the intervention, particularly in an evaluation of nurtureUK VRU programmes to reduce school exclusions. Participants in focus groups conducted by Middleton (2022) reported that seeing the positive effect of the approach led to increased buy‐in:

Come September there will be a team of teachers, one in each department – they volunteered – this will then be rolled out in every department. If the impact is shown, then more will be allowed.

(Participant; Middleton 2022, 77)

Even in situations where teachers had struggled to get their colleagues to buy into the intervention, they noted the importance of facilitating implementation and that it was not necessarily ever going to be an easy task. For example,

…it's like a universal change, isn't it? You know, and I think that takes time and it's gonna be an ongoing process.

(Teacher/Senior Leadership team; Blandford‐Elliott 2021, 65)

At the leadership level, buy‐in was facilitated by an openness to change from senior leadership teams and enthusiasm for adopting new strategies to meet the needs of their students (Humphrey et al. 2018). As one Deputy Headteacher said:

We just thought it was a brilliant opportunity to see another strategy or another way that it could be used so that maybe we could implement it, ourselves afterwards or completely change our behaviour strategies … [the school is] quite open to anything really … [it] just sounded like the kind of thing that we are into when we initially read about it.

(Deputy Headteacher; Humphrey et al. 2018, 62)

##### 7.2.4.2 Subtheme Barrier: Lack of ‘Buy‐in’

Unsurprisingly, the converse was also true. When members of the school community did not support or engage with the intervention, this was a notable barrier to implementation. Participants noted that a lack of buy‐in from their colleagues was a barrier to implementation, but that this was not unique to the specific intervention, and one way to overcome this was to highlight students' enjoyment and enthusiasm for the intervention. As one teacher who took part in the evaluation of SWPBIS in Welsh primary schools noted:

Barrier‐wise … just that initial getting everyone onboard, persuading them, “Look, this is gonna work,” which happens with any new initiative that you take on any way. You always get the odd one or two that are wary … You lead by example and you just keep positive … show them the little rewards that are happening … and the children, getting them enthused about it, staff can't say no. Once you've got enthusiastic children that love something, that's it.

(Teacher/Senior Leadership team; Blandford‐Elliott 2021, 65)

A lack of buy‐in and support from school leadership made it difficult for participants in some interventions to make the necessary changes and thus, this was a notable barrier (e.g., Allen et al. 2020; Middleton 2022). Buy‐in was a barrier identified by Middleton (2022), and in this study, a lack of buy‐in was attributed to a lack of time for training and developing staff confidence in new approaches—primarily caused by COVID‐19 lockdowns.

Lack of buy‐in was often attributed to a lack of motivation to change, with some members of staff described as preferring a ‘more traditional approach’ and wishing to continue their normal practice (Sparling et al. 2022, 46), also not homogenous across all members of the school community. Blandford‐Elliott (2021) found that certain members of staff were particularly reluctant to engage with the intervention and the change that ensued. For example,

…one of the main barriers was mobilising, motivating certain members of staff initially, some staff were like, “Well, we don't need it. We haven't got a problem with behaviour in my classroom.”

(Teacher/Senior Leadership team; Blandford‐Elliott 2021, 65)

…we all had to live it, we all had to believe it … Getting everyone to buy into it really was the tricky part, and I don't think some would ever buy in to anything. I think they've been teaching a little bit too long, and they believe they've seen everything come and go, haven't they.

(Teacher/Deputy Headteacher; Blandford‐Elliott 2021, 65)

Buy‐in was a particular challenge when it was leadership that were not engaged. This greatly impacted the implementation of the intervention activities, such as meetings, and was also hindered by high levels of changeover in school leadership (Blandford‐Elliott 2021).

#### 7.2.5 Seeing Results

Many process evaluations noted that seeing the impact of the intervention was a facilitator to implementation and was perceived as improving buy‐in, increasing engagement and overall, contributing to participants' positive perception of the intervention programmes. In the context of our qualitative evidence synthesis, this impact refers to changes in student behaviour and/or discipline. However, some participants also highlighted how not seeing a desirable impact was a barrier to implementation. The following sections outline these subthemes in further detail.

##### 7.2.5.1 Subtheme Facilitator: Identifying Impact

When participants were able to identify the impact that the intervention had, or was having, on students in their schools and classrooms, this was a considerable facilitator to implementation. For example, Middleton (2022) reported that participants who saw the impact of the intervention on school exclusions were more likely to want to continue participation, but also roll out implementation on a larger scale. For example:

The Head[teacher] is impressed and wants to push it out further next year using [nurture practice] in bigger groups.

(Participant; Middleton 2022, 77)

Many participants noted that they felt the intervention had a positive impact on their students' behaviour in class, and this was an important facilitator to implementation. Some evidence of positive impacts came from anecdotal evidence in the evaluation of ACE‐Informed Schools (Sparling et al. 2022). In particular, teachers noted specific improvements for students with internalising and externalising behavioural needs, who may be at increased risk of being excluded from school. In this evaluation, teachers appreciated the opportunity to try to understand why some children behave in certain ways and to modify how they respond accordingly:

…if he does have an outburst, we can … I mean, sometimes you just have to send him out, say you need to go out and you need to bounce, or whatever, but actually everybody's like that in a situation sometimes, and it's much better than he's not, um … lashing out so much. We have had a couple of incidents, but it is much better, which is good. And he, he can talk things through and understand things a bit more…

(Teacher, Stanbridge and Campbell 2016, 272)

This was true too in the evaluation of nurtureuk. The positive impact on students at‐risk of exclusion led to a change in staff attitudes and increased inclusion in classroom settings. Middleton (2022) noted that following positive changes due to participation in the intervention, staff seemed to be less likely to exclude these children from their classrooms and were motivated to find solutions and help these children. For example:

Staff who wouldn't have them in their class before are now outside with them and able to give [them] praise … staff are excited – they want to work with them.

(Participant; Middleton 2022, 77)

Often, this impact was used by participants to encourage their colleagues to continue and persist with implementation. For example, a teacher interviewed by Blandford‐Elliott 2021, 67) in a process evaluation of SWPBIS said:

What they [headteacher and deputy headteacher] would do is because you would naturally have days where you've had a bad day with your class and you'd go, “This isn't working” and they would say, “But this isn't going to be an overnight success. This isn't something that's going to work for you after two or three lessons, and you're gonna have bad days.” And they would point out X‐child in the classroom that is responding really well to it … And then you get a pick‐me‐up that way.

Teachers commented that seeing results and the impact the intervention could have made them more likely to continue implementation and to use the intervention strategies in the future. Members of school leadership teams also noted that seeing an impact across the school was a key facilitator. Seeing the change in behaviour not only increased engagement, but it also improved buy‐in across the school community, as is discussed in Section 7.2.4. Students also noted that the impact of the intervention was a significant facilitator. In an evaluation of the Risk Avert screening tool and small group workshops, students commented that the intervention was effective – and that they knew some of their peers would also benefit from participation.

In fact one group of girls said … we think more girls need to do this.

(Teacher; Wright 2020, 254)

Similarly, in an evaluation by Stanbridge and Campbell (2016), students themselves were able to identify the ways in which the intervention had impacted them personally, and teachers/staff noted that participants had made progress on both internalising and externalising behavioural issues. One teacher in particular said that the intervention may have reduced the need to exclude or remove children from the classroom:

…if he does have an outburst, we can … I mean, sometimes you just have to send him out, say you need to out and bounce, or whatever, but actually everybody's like that in a situation sometimes, and it's much better that he's not, um … lashing out so much. We have had a couple of incidents, but it is much better, which is good. And he, he can talk things through and understand things a bit more.

(Teacher; Stanbridge and Campbell 2016, 272)

Warren et al. (2020) observed that participation in action groups, as part of the Learning Together intervention, had a positive impact on the behaviour of students who were involved in these groups. However, as it would not be feasible to include every student in these action groups, this could be a limitation of the intervention. Yet, Warren et al. (2020) found that by selecting students carefully for participation, this could actually have a wide‐reaching impact on behaviour across the school. As one teacher reported:

We have a boy … A proper naughty boy. But he has shown such maturity in the final part of his year‐11 and he's been, I think, an outstanding student in year‐12. But all the kids know who he is or they know of the local family. And so, if he's on board [with the action group], that sends a really important message. And I think that is critical.

(Staff; Warren et al. 2020, 9)

##### 7.2.5.2 Subtheme Barrier: Failing to See Results

When participants failed or struggled to identify the impact of interventions, this was often a barrier to implementation and discouraged teachers and school staff from continuing with implementation. For example, a lack of seeing results was a clear barrier to implementation of the GBG as noted by teachers interviewed by Humphrey et al. (2018). When teachers were expected to invest a lot of time into implementation, but could not see the impact, this discouraged them from continuing with the programme. As one teacher, from a school that ultimately exited the evaluation early, commented:

I found that every single time … I was getting quite frustrated with how the game was working … I was waiting for it to get better, and it wasn't getting better.

(Teacher, exit interview; Humphrey et al. 2018, 42)

Multiple participants in included evaluations also noted a limited impact of the intervention on certain students (e.g., Stanbridge and Campbell 2016). Teachers interviewed by Allen et al. (2020) who took part in an evaluation of the Incredible Years classroom management programme found that the impact of the intervention was limited for students with challenging behaviour. For example, as one teacher noted:

I'm just not convinced that [the course] has worked for my main offenders if you like, the ones who have ODD and ADHD and all of that kind of thing. I don't think it's really worked for them […] but for general classroom management it definitely has worked.

(Teacher; Allen et al. 2020, 1167)

While this does not seem to be seen as a barrier to implementation by teachers themselves, it is a barrier in the context of the current synthesis, as these students with challenging behaviour may be at increased risk of exclusion. Therefore, it is possible that an intervention may be perceived as effective for the classroom, but not for those most in need.

Yet, an interesting contrast is that teachers in the evaluation of the GBG thought that the intervention did have a desirable impact on children in their classroom who were identified as being at‐risk for conduct disorder. One teacher said, speaking in relation to two male students with behaviour plans:

…trying to engage those two boys in particular, was really tricky. [But as time went on] … he's really turned it round he's been a little superstar recently … he quickly proved himself and he's already, I've put him as a team leader now because he's responsible … so it's having an effect on him.

(Teacher; Humphrey et al. 2018, 65)

A key difference between these two intervention programmes may be the reason for this contrast. While the Incredible Years classroom management programme for teachers, the GBG focuses on a specific activity to be implemented with students and involves collaborative group work among students. This contrast demonstrates that addressing the behaviour of students, and reducing the need to exclude children from school, is not a one‐size‐fits‐all exercise.

As such, interventions that are effective and result in desirable changes in student behaviour, such as, decreased disruptive behaviour and the need to use exclusionary discipline, facilitated the implementation of school‐based interventions and improved participants' perceptions of programmes. Overall, when the intervention was seen as ‘not working’, this was a barrier to implementation.

## 8 Discussion and Conclusions

### 8.1 Quantitative Analysis

The present multi‐level meta‐analysis investigated the effectiveness of school‐based interventions in reducing school suspension and related outcomes. The analysis revealed several noteworthy findings that shed light on the complex relationship between these interventions and disciplinary measures.

#### 8.1.1 Overall Reduction in School Suspension

The primary finding of this meta‐analysis indicates that school‐based interventions had a small, positive, and statistically significant impact on reducing school exclusions when comparing the intervention group to the control group (SMD = 0.106; [95% CI: 0.039 to 0.173]; *p* < 0.001). This suggests that participants in these interventions were less likely to be excluded from school. However, it is important to note that the effect size, while statistically significant, was relatively small. This suggests that while school‐based interventions are associated with a reduction in suspension rates, the practical significance of this reduction may vary across different contexts and populations.

The results of this updated meta‐analysis differ from the first version of this review (Valdebenito et al. 2018), where the overall impact reported was higher (SMD = 0.30; [95% CI: 0.20 to 0.41]; *p* < 0.001). There are several factors that may contribute to this difference. For example, the updated version includes a larger number of studies and also expanded the scope of the previous review by including both QED and RCTs. What is more, the updated version uses refined analytical methods to account for correlated outcomes, that is, the use of multilevel meta‐analysis techniques with robust errors. On the other hand, Mielke and Farrington (2021) conducted a meta‐analysis sharing a similar objective with the present review by evaluating the effectiveness of school‐based interventions in reducing suspensions and related outcomes. However, there are key differences in scope, methodology, and findings. Mielke and Farrington's review included only 14 RCTs conducted between 2008 and 2019 with a minimum sample size of 100 students, focusing exclusively on arrests and suspensions. Their results showed very small, non‐significant reductions in suspensions and arrests overall, but highlighted that well‐implemented interventions and those delivered in high schools produced more favourable effects. In contrast, our review employed a broader inclusion strategy encompassing both RCTs and high‐quality QEDs, resulting in a larger evidence base (67 studies and over 276 effect sizes), spanning a wider time frame (1980–2022) and addressing both primary (suspensions) and secondary outcomes (e.g., delinquency, conduct problems). Our results display a null or negative effect on behavioural outcomes.

The present review reports the findings from additional separate analyses for different types of school exclusion to examine whether there were any possible differences. While the results for ‘general suspension’, showed a small, positive, and statistically significant reduction, indicating that school‐based interventions were most effective in reducing this form of disciplinary action this was not the case for all types of school exclusion. For more severe disciplinary measures, such as out‐of‐school suspension and expulsion, the interventions had a smaller and often non‐statistically significant impact. This suggests that while these interventions can be effective in addressing less severe behavioural issues, they may be less effective in preventing more serious disciplinary issues, as far as we know at present.

Secondary outcomes: The exploration of secondary outcomes related to the impact of interventions designed to reduce school exclusion on externalising behaviours, including conduct problems, delinquency, violence, and substance use, revealed mixed results. In some cases, the interventions had no significant impact, and in others, they even demonstrated possibly harmful effects. For example, the evaluation by Obsuth et al. (2017) reported that the intervention resulted in an increase in problematic behaviours. As such, school‐based interventions may have unintended consequences on these secondary outcomes if not carefully designed and implemented. The findings of the present review also found no impact of school‐based interventions on behavioural problems such as conduct problems, violence, delinquency, or substance use. In summary, the interventions designed to mitigate school exclusion showed only a modest effect, with outcomes indicating either no discernible impact or, in some cases, a potentially adverse effect on behaviour and substance use. From a practical perspective, it was evident that students who underwent disciplinary measures for school exclusion did not manifest significant changes in their serious behavioural issues. This observation underscores the complexity of addressing the underlying causes of exclusionary behaviour within school settings. It suggests that a one‐size‐fits‐all approach may not adequately address the needs of students, particularly those grappling with more severe behavioural challenges. As suggested by previous research (e.g., Kovalenko et al. 2022), it is increasingly apparent that students exhibiting violent or delinquent behaviours may require more tailored and specialised interventions to effectively address their individual circumstances and foster positive outcomes. Therefore, future research may benefit from a nuanced understanding of the diverse needs of students and the implementation of targeted interventions that cater to their specific requirements.

#### 8.1.2 Moderator Analysis

Heterogeneity in Results: The significant heterogeneity observed in the meta‐analysis results is not surprising given the wide range of school‐based interventions included in the studies. Moreover, heterogeneity is somewhat expected in a meta‐analysis, and is not necessarily a limitation (Higgins and Green 2011). Post hoc analysis of heterogeneity allowed for the examination of the possible sources and causes of differences, thus furthering our understanding of ‘what works, for whom, and in what context’. These interventions varied in terms of their content, duration, and target populations. Additionally, the studies encompassed different geographic locations (including different countries) and school grade levels, all of which contributed to the inherent variability in outcomes.

We included several possible moderator variables that may explain the heterogeneity between primary evaluations. Overall, the findings were mixed. For example, the gender of participants did not significantly influence the effectiveness of interventions, suggesting that these programmes may be equally beneficial for male and female students. The age of participants was also explored as a possible moderator. Age was represented by school grade, and the results suggest that school‐based interventions to reduce school exclusions had an overall desirable impact in primary schools, although the mean effect size was relatively small. This effect did not significantly vary for middle and high school students, indicating that we should seek to understand more about which interventions work ‘best’ for which age groups.

Our findings also suggested that the role of the evaluator had a significant impact on the effectiveness of included interventions. As in the previous review (Valdebenito et al. 2018), we observed that independently conducted evaluations (i.e., those where evaluation teams were not programme developers or part of the delivery team) yielded smaller effect sizes when compared to studies carried out by researchers intimately involved in programme development and implementation. This finding is consistent with previous research on the influence of the involvement of programme developers in assessment of effectiveness (Eisner 2009; Eisner and Humphreys 2012; Lösel and Beelmann 2006; Petrosino and Soydan 2005). Indeed, previous meta‐analyses of school‐based interventions have found that when an intervention is evaluated by the developer, the overall impact is higher (Gaffney et al. 2019). Therefore, this result highlights the advantages of conducting independent trials to generate more precise results.

A moderator analysis of the different types of school‐based interventions showed mixed results. Overall, there were no significant differences in the mean effect sizes for different types of interventions. While some interventions, such as academic skills enhancement and mental health programmes, were associated with small positive effects, others, such as mentoring, were associated with a null effect. The lack of significant differences suggests that the effectiveness of these interventions may depend on various contextual factors, and no single type of intervention emerges as superior.

In relation to restorative programmes, a recent non‐probability survey conducted among teachers in the United Kingdom revealed some notable insights. It was found that approximately half of primary school teachers and nearly 6 out of every 10 secondary school teachers reported that restorative justice or restorative conversations were explicitly mentioned in their school's behaviour policy (Allen et al. 2023). However, despite the prevalence of these practices, their effectiveness in reducing exclusion or suspension rates remains somewhat limited based on these findings.

It is possible that the effectiveness of RP, as reported in the present review, is explained by methodological limitations of the included evaluations. For example, two included evaluations of RP, Augustine et al. (2018) and Huang et al. (2023), could be described as underpowered cluster‐RCTs. Huang et al. (2023) had the larger sample size of these two evaluations, and yet it is still underpowered. This evaluation measured the impact of RP with 18 clusters (in this case, schools) and a total of 5878 students, which averages to approximately 327 students per school. When we assess the statistical power of this study, and assume a normally distributed outcome, without considering potential benefits from factors like stratification or baseline controls, the estimated statistical power of the study is only 40%. In practical terms, this means that even if RP had a real effect, studies of this size would only have a 40% chance of detecting it in 40 out of 100 similar studies.

We conducted a multiple‐predictor meta‐regression using the *metafor* package in r‐studio. The regression analysis examined various factors, including the role of the evaluator, grade at school, percentage of male students, and a variable representing the score of RoB for each included study. From the results, one significant finding emerged: the intervention effect was notably higher for interventions focused on improving teachers' skills. It is worth noting, however, that the number of studies centred on teachers' skills improvement in this analysis was limited (i.e., 4 studies and 10 effect sizes).

Overall, school‐based interventions have the potential to reduce school suspension rates, especially for general suspension, but their impact may be limited in preventing more severe forms of exclusion and addressing externalising behaviours. The significant heterogeneity across studies underscores the need for tailored approaches that consider the specific context, target population, and goals of interventions. Further research is essential to identify the most effective strategies for improving student behaviour, reducing disciplinary actions, and promoting a positive school environment. Additionally, careful programme design and evaluation by those intimately involved in the intervention's development and implementation may enhance their effectiveness.

### 8.2 Qualitative Analysis

Our qualitative evidence synthesis included nine process evaluations of school‐based interventions implemented in UK schools. Findings concerning participants' perceptions of barriers and facilitators to the implementation of interventions were extracted, and using a thematic approach, four main themes were identified. These were: (1) Intervention format; (2) (in)Consistency; (3) Buy‐in; and (4) Seeing results. Multiple subthemes were identified for each main theme, relating to both facilitators and barriers to implementation. The following section summarises the main findings relating to each of the four main themes.

#### 8.2.1 Intervention Format

Overall, the format of the intervention (i.e., how and what was actually implemented) was perceived as a facilitator to implementation. As highlighted in the supporting quotes from teachers themselves, positive elements of the intervention format related to the inclusion of group discussion and collaboration, flexibility, and structure. Teachers appreciated the opportunity to work in groups, especially with supportive group leaders (e.g., Allen et al. 2020; Humphrey et al. 2018). Group discussion was particularly positive when it allowed for discussion and collaboration with participants from other schools; providing different perspectives and shared experiences (e.g., Warren et al. 2020). Flexible interventions that were adaptable to different contexts, classrooms, and individual students were also viewed positively. Intervention formats that involved a ‘planning’ element were also perceived well (e.g., Stanbridge and Campbell 2016).

However, elements of the intervention format were also perceived as barriers to implementation—for example, outdated, unsuitable, and expensive intervention materials (e.g., Wright 2020). Moreover, a common barrier to implementation was when teachers noted that the intervention did not fit the context of a UK school or classroom, or in other words, there was a barrier in the transferability of the programme (e.g., Ashworth 2018; Humphrey et al. 2018). This was particularly an issue with ‘Americanised’ interventions, or those which may have been developed in the United States and not adapted or adjusted appropriately before implementation in the United Kingdom.

#### 8.2.2 (In)Consistency

Many teachers noted how their existing school policies on behaviour and discipline were inconsistent, and as such, a facilitator to implementation and continued implementation of interventions was when new policies were seen as being consistent (e.g., Blandford‐Elliott 2021). Interventions that involved a whole‐school approach encouraged consistent implementation across all members of the school community, and this was a notable facilitator to implementation (e.g., Blandford‐Elliott 2021). In the same vein, interventions that did not involve a whole school approach often faced the barrier of inconsistent implementation (e.g., Allen et al. 2020). Teachers felt that if all staff members are not trained or educated about intervention strategies, this may ultimately undermine the impact and delivery of these approaches.

#### 8.2.3 Buy‐in

Buy‐in, and a lack thereof, was another main theme that emerged from the data in our qualitative evidence synthesis. When there was a good degree of ‘buy‐in’ from staff and participants, this was a notable facilitator to implementation, especially when school leaders bought into the intervention (e.g., Middleton 2022). Teachers who took part in intervention programmes also noted that a lack of buy‐in was a significant barrier, and that they often struggled to encourage their colleagues to respond to new approaches or strategies (e.g., Blandford‐Elliott 2021). Buy‐in was particularly difficult with certain groups of staff, such as colleagues who may not teach or interact with children with disruptive behaviour or colleagues with many years of experience (e.g., Sparling et al. 2022).

#### 8.2.4 Seeing Results

Finally, the effect of seeing the impact of an intervention on student behaviour was a considerable facilitator to the implementation of programmes (e.g., Middleton 2022; Warren et al. 2020). Although the purpose of our qualitative evidence synthesis was not to assess participants' perceptions of impact, many teachers and school leaders noted that when they could see desirable changes in student behaviour because of the intervention, this was a key facilitator. Seeing results was not only a key facilitator to implementation, but also to continued implementation of programmes following cessation of the evaluation (e.g., Middleton 2022). Often, desirable changes did not need to be observed across a whole school, or even multiple children in a classroom; teachers responded positively and favourably when change was observed with just one student (e.g., Warren et al. 2020). This has particular importance considering of the findings from our meta‐analytical examination of impact evaluations. The overall finding suggests that included evaluations had a relatively small impact on school exclusions. However, it may be that even small changes are desirable to teachers and indicate a need for further and wider implementation of intervention programmes.

### 8.3 Limitations and Future Research

#### 8.3.1 Quantitative Analysis

The present review faces several limitations that may impact the generalisability and reliability of the results. It is crucial to acknowledge that the included studies provide limited information for assessing quality bias. Notably, approximately 36% of the studies included in our analysis reported results based on samples comprising fewer than 200 participants. The small sample sizes in some primary research studies impose clear constraints on our ability to accurately estimate the effects of interventions accurately, even when aggregated across a meta‐analysis. Another source of bias, one that is arguably beyond the control of a meta‐analyst, is the large number of studies that did not conduct an independent evaluation. In 45% of included evaluations, the developer of the intervention was also involved in the evaluation of impact, and these studies were associated with higher overall effectiveness. As such, our findings may be biased due to this issue. However, future research is needed to determine whether this is truly a malicious form of bias. For example, it is plausible that evaluations that involve the programme developer benefit from greater implementation fidelity and this is the cause behind the larger effects (Valdebenito et al. 2018). Indeed, findings from our qualitative evidence synthesis suggest that teachers in UK schools appreciated involvement from researchers and those with expert knowledge. This was perceived as a facilitator to implementation, and as such, further investigation to better understand this relationship is needed.

Regarding the searches, although we try to be inclusive and comprehensive, we did not conduct dedicated searches of conference proceedings databases or major education conference archives (e.g., AERA, EERA, BERA). However, throughout the screening process, any conference abstracts or proceedings that appeared in the general database results were screened for eligibility on a case‐by‐case basis. We acknowledge that this may have resulted in the omission of some relevant grey literature, and we agree that this represents a limitation. In addition, we acknowledge that, while we did not apply any language restrictions in our eligibility criteria, the scope of our database searches was limited by resource constraints. As noted, SciELO was included specifically to capture relevant content from Latin American and Iberian sources. Although we were not able to search more databases that index non‐English language studies, two members of the review team are fluent Spanish speakers, which allowed us to screen and assess relevant Spanish‐language literature retrieved through our existing strategy.

Regrettably, a notable limitation of the present review is the disproportionate inclusion of research conducted in the United States. However, this is not certainly an issue unique to the present meta‐analysis. While the United States has produced a substantial body of research on interventions aimed at reducing school suspensions, the overrepresentation of American studies raises concerns about the generalisability of the findings to other regions and educational contexts (e.g., Cartwright and Hardie 2012). This concentration of evidence from a single country limits our ability to draw generalisable conclusions and insights, as educational systems and practices vary significantly between countries. Studies conducted in the United States may offer valuable insights into interventions' efficacy; however, the applicability of these findings to educational landscapes worldwide may be limited. The cultural, socioeconomic, and systemic differences between different countries could significantly influence the effectiveness of interventions targeting school exclusion. Therefore, it is essential to acknowledge this geographical bias and exercise caution when extrapolating the results of American studies to educational settings in other parts of the world. To establish a more comprehensive and globally relevant understanding of interventions to reduce school exclusion, future research efforts should strive for greater geographical diversity in their study samples and contexts. The inclusion of languages other than English could also add more evidence to the analysis.

#### 8.3.2 Qualitative Analysis

The main limitation to our qualitative evidence synthesis was the lack of available and includable research in the United Kingdom on interventions to reduce school exclusions. We initially intended to synthesise the qualitative literature on perceptions of barriers and facilitators to the implementation of school‐based interventions to reduce or prevent children being excluded from school in the United Kingdom. There is quite a lot of literature in a UK context surrounding the issue of school exclusions, but existing studies tend to either interpret national statistics (e.g., Black 2022) or explore a range of perspectives on the use and experiences of exclusionary discipline (e.g., Done and Knowler 2021; Embeita 2019). We were only able to identify one UK process evaluation that explored participants' experiences of interventions to specifically reduce or address the issue of school exclusions (i.e., Middleton 2022). The remaining included evaluations focused on interventions to reduce disruptive behaviour in school.

Given the polarised debate in the United Kingdom, especially in England and Wales, surrounding the use of exclusionary discipline in schools, future research could aim to synthesise studies that examined teachers, school leadership, students, and parents' views and experiences of school exclusion. In particular, future qualitative evidence syntheses could explore the evidence on the reasons and justifications for excluding a child from school and the use of other alternatives that are possibly used (e.g., ‘off‐rolling’ and ‘managed moves’). Similarly, future research could examine the experiences, barriers and facilitators to Alternative Provision services for children who are excluded from school. School exclusion, while the focal outcome here, is effectively an indicator of student conduct or a point at which modifications in the management and/or response to student behaviour can be introduced. We also have to be realistic that the elimination of exclusion as a disciplinary sanction, as is sometimes advocated, would likely be met with strong opposition, particularly from teachers. Again, based on data from UK teachers, nearly 90% who responded to a survey (with responses weighted) disagreed with the statement ‘schools should never exclude students, for any reason’ (Allen et al. 2023). Furthermore, removing the option of excluding a child from school may create situations where schools are unsafe for all students and teachers, particularly when interventions to address the root causes of exclusion (i.e., disruptive and often violence/aggressive behaviour) are not put in place. That would not rule out, for example, approaches to try and reduce the use of exclusion in high‐excluding schools that relied more on ‘light touch’ approaches, such as behavioural nudges, as has been used effectively in health care settings (Hallsworth and Sanders 2016).

Another possible limitation to our qualitative evidence synthesis is our use of thematic analysis to interpret findings. Thematic analysis is often seen as a flexible approach to understanding qualitative data, and a number of steps and guidelines are available (Braun and Clarke 2006). However, we used an inductive data‐driven approach to the interpretation of qualitative data from included process evaluations. Some experts in primary qualitative research suggest that a hybrid approach to thematic analysis, whereby both data‐driven and theoretical frameworks are used to interpret findings (Proudfoot 2023). Similarly, the methods and development of qualitative evidence synthesis is a growing area of research, and so perhaps future updates of this review will avail of more advanced methods.

Our qualitative evidence synthesis could also be improved by incorporating global research to improve the generalisability of the findings to additional educational contexts. Despite a vast variety of educational systems, approaches, and standards worldwide, there are lessons to be learned from looking at evidence outside of a national scope, such as in our review. This is an interesting avenue for future qualitative evidence syntheses. Similarly, the generalisability and comparability of our findings may be impacted by the broad range of interventions included in the review. It is possible that teachers' perceptions of the barriers and facilitators to intervention implementation may vary across different types of interventions. A possible avenue for future research would be to examine the qualitative evidence specific to one type of intervention (e.g., whole‐school approaches) or one frequently evaluated intervention (e.g., SWPBIS only). Moreover, perhaps future research could compare the perceived barriers and facilitators to implementation across different types of interventions.

One key takeaway from our qualitative evidence synthesis is that there is a need for more research in this area, as well as a need for continued development, implementation, and evaluation of school‐based programmes to reduce the use of, and need for, exclusionary discipline. As one teacher noted, ‘no one strategy will work for everyone or forever’ (Allen et al. 2020). Therefore, we must continue to improve our knowledge of what works and how to best implement effective interventions in this area.

### 8.4 Conclusions

Based on the evidence presented in this review, several key conclusions can be drawn. First, the meta‐analysis revealed that school‐based interventions produced a small but statistically significant reduction in school exclusions, indicating that such programmes can play a modest role in reducing the use of suspensions. However, the effect size was smaller than reported in the previous version of this review, likely due to the inclusion of a broader range of study designs (including quasi‐experiments) and a more conservative analytical approach using multilevel modelling with robust variance estimation.

Second, while interventions were somewhat effective in reducing general suspensions, they were notably less effective in preventing more severe forms of exclusion, such as out‐of‐school suspensions and expulsions. Furthermore, secondary outcomes relating to externalising behaviours—such as conduct problems, violence, and substance use—showed limited or no beneficial effects. In some cases, interventions were associated with adverse impacts, highlighting the importance of careful programme design, implementation, and monitoring.

Third, moderator analyses suggested that context and implementation characteristics matter. For example, interventions implemented in primary schools tended to show better outcomes than those in secondary schools. Additionally, independent evaluations were associated with smaller effect sizes compared to those conducted by programme developers—raising important considerations for bias and fidelity. Notably, interventions focused on improving teacher skills were associated with more promising results, though this finding was based on a small number of studies.

Fourth, the qualitative synthesis added further nuance, identifying four major themes—intervention format, consistency, buy‐in, and perceived impact—that shaped the success or failure of implementation efforts in UK schools. These themes underscored the importance of flexible, context‐sensitive delivery and strong support from school leadership. Teachers were more likely to sustain interventions when they observed even small behavioural improvements in students.

Together, the quantitative and qualitative findings indicate that school‐based interventions can contribute to reducing exclusions, particularly for less severe behaviours. However, they are not a panacea. The effects are generally modest, context‐dependent, and likely require complementary strategies tailored to individual and school‐level needs. Interventions for students exhibiting more serious behavioural issues may need to be more specialised and intensive. As such, future efforts should focus on designing, implementing, and evaluating targeted programmes that respond to the complex factors underlying school exclusion.

## Transparent Peer Review

The peer review history for this article is available at: https://www.webofscience.com/api/gateway/wos/peer-review/10.1002/cl2.70063.

## Supporting information

APPENDIX.

## Endnotes

1 We use UK‐specific terminology to describe students with additional learning needs in line with language and labels used by the Department for Education (DfE 2023). These labels and the thresholds used to identify students with additional learning needs may vary between contexts and countries.

2 In the United Kingdom, private schools can also be referred to as independent schools or public schools. Our review only includes state schools, and similar institutions across the world. These are schools that are publicly funded and follow a national curriculum (https://www.gov.uk/types-of-school).

3 Originally, searches for qualitative evidence synthesis were performed in November 2022. However, due to an administrative error, the exact syntax for these searches was not retained. Therefore, these searches were replicated in September 2023 to meet MECCIR reporting standards. The same timeframe and keywords were used and no new studies were identified in these later searches.

4 Indeed, many combinations of search terms were tested in these databases alongside indicators for UK‐based studies. No search results were returned in any of our pilot searches.

5 This total number includes 57 studies. Ten other papers did not report the number of students involved in the analysis. They mention number of schools or districts.

6 Employing a three‐tier model is justified only if it captures the nuances of data variability more effectively than a two‐tier model. Should a two‐tier model exhibit an equivalent level of fit to that of a three‐tier model, the principle of *Occam's razor* suggests a preference for the simpler two‐tier model, as it offers an equally satisfactory explanation of our data with reduced complexity (Harrer et al. 2021). When tested, the three‐tier model shows a better fit with the AIC and BIC being lower (favourable performance).
