# Supplementary material for: School‐Based Interventions for Reducing Disciplinary School Exclusion. An Updated Systematic Review
Source: Campbell Syst Rev. 2025 Oct 22;21(4):e70063. doi: 10.1002/cl2.70063 (PMC12541690; doi:10.1002/cl2.70063)
Supplement: Supplementary file 1 — APPENDIX. [file CL2-21-e70063-s001.docx]

**APPENDIX A: Search syntax**

**Electronic searches**

**Australian Education Index (via ProQuest)**

**Conducted:** 11/11/2022

**Timeframe:** Oct/2015 – Nov/2022

**Total results:** 74

**Note:** results imported to Covidence

**Final searches string:**

1. (school exclusion OR school suspension) AND (rct OR experiments OR quasi-experiments OR impact OR intervention OR program)
2. ((evaluation) OR (effectiveness) OR (program*) OR (impact) OR (effect) OR (experimental) OR (quasi-experimental) OR (RCT) or ("random evaluation") OR ("efficacy trial") OR ("process evaluation"))

AND (("disciplinary methods") OR ("token economy") OR ("classroom management") OR ("school management") OR ("early intervention") OR ("school support project") OR ("skills training"))

AND ((schoolchildren) OR (pupils) OR (children) OR (adolescents) OR ("school-aged children") OR (student) OR (youth) OR ("young people"))

AND (("school exclusion") OR ("suspension reduction") OR ("out-of-school suspension") OR ("in-school suspension") OR ("out-of-school exclusion") OR ("in-school exclusion") OR (suspended) OR (suspension) OR (expelled) OR (expulsion) OR ("outdoor suspension") OR (stand-down) OR ("exclusionary discipline") OR (discipline))

**British Education Index (via EBSCO)**

**QUANT**

**Conducted:** 8/11/2022

**Timeframe:** Oct/2015 – Nov/2022

**Total results**: 95

**Note:** results imported to Covidence

**Final searches string:**

((evaluation) OR (effectiveness) OR (program*) OR (impact) OR (effect) OR (experimental) OR ("quasi-experimental") OR (RCT) or ("random evaluation") OR ("efficacy trial") OR ("process evaluation"))

AND (("disciplinary methods") OR ("token economy") OR ("classroom management") OR ("school management") OR ("early intervention") OR ("school support project") OR ("skills training"))

AND ((schoolchildren) OR (pupils) OR (children) OR (adolescents) OR ("school-aged children") OR (student) OR (youth) OR ("young people"))

AND (("school exclusion") OR ("suspension reduction") OR ("out-of-school suspension") OR ("in-school suspension") OR ("out-of-school exclusion") OR ("in-school exclusion") OR (suspended) OR (suspension) OR (expelled) OR (expulsion) OR ("outdoor suspension") OR (stand-down) OR ("exclusionary discipline") OR (discipline))

**QUAL**

**Search #1**

**Conducted:** 16/09/23

**Timeframe:** Oct/2015 – Dec/2022

**Total results:** 3

**Final searches string:**

(“Effect” OR “Effectiveness” OR “Evaluation” OR “Impact” OR “Intervention” OR “Process Evaluation” OR “Programme” OR “Programme Effectiveness”) AND (“Classroom intervention” OR “Classroom Interventions” OR “Classroom management” OR “Classroom management programme” OR “Classroom Strategies” OR “Disciplinary methods” OR “Early intervention” OR “Early interventions” OR “School management” OR “School support projects” OR “Skills training OR Token economy”) AND (“Adolescent” OR “Adolescents” OR “Children” OR “Pupils” OR “School children” OR “School-aged children” OR “Student” OR “Students” OR “Young People” OR “Youth”) AND (“Access” OR “Barriers” OR “Discipline” OR “Exclusionary discipline” OR “Expelled” OR “Expulsion” OR “Facilitators” OR “Implementation” OR “In-school exclusion” OR “In-school suspension” OR “Out-of-school exclusion” OR “Out-of-school suspension” OR “Outdoor suspension” OR “School exclusion” OR “School exclusion reduction” OR “Stand-down” OR “Suspended” OR “Suspension” OR “Suspension reduction”) AND (“Ethnography” OR “Focus group” OR “Interview” OR “Qualitative” OR “Qualitative data”) AND (“United Kingdom”)

**Search #2**

**Conducted:** 16/09/23

**Timeframe:** Oct/2015 – Dec/2022

**Total results:** 2

**Final searches string:**

(“evaluation” OR “effectiveness” OR “intervention” OR “programme” OR “process evaluation” OR “impact” OR “effect” OR “process”) AND (“disciplinary methods” OR “token economy” OR “classroom management” OR “classroom intervention” OR “classroom strategies” OR “school management” OR “early intervention” OR “school support projects” OR “skills training”) AND (“school children” OR “pupils” OR “adolescent” OR “student” OR “youth” OR “young people” or “school-aged children”) AND (“school exclusion” OR “suspension” OR “suspended” OR “expelled” OR “expulsion” OR “outdoor suspension” OR “stand-down” OR “exclusionary discipline” OR “implementation” OR “facilitators” OR “access” OR “barriers”) AND (“qualitative” OR “qualitative data” OR “interviews” OR “focus groups” OR “ethnography”) AND (“United Kingdom”)

**Search #3**

**Conducted:** 16/09/23

**Timeframe:** Oct/2015 – Dec/2022

**Total results:** 2

**Final searches string:**

(“process evaluation” OR “intervention” OR “programme” OR “effectiveness”) AND (“token economy” OR “classroom management” OR “early intervention” OR “skills training”) AND (“pupils” OR “students” OR “youth” OR “adolescent” OR “children”) AND (“school exclusion” OR “expulsion” OR “expelled” OR “suspended” or “suspension” OR “discipline” OR “access” OR “barriers” OR “facilitators”) AND ("qualitative” OR “interview” OR “focus group” OR “ethnography”) AND  (“United Kingdom”)

**Search #4**

**Conducted:** 16/09/23

**Timeframe:** Oct/2015 – Dec/2022

**Total results:** 245

**Final searches string:**

1. “school exclusion” AND “qualitative” [12 results]
2. “Suspension” AND “intervention” AND “evaluation” [1 result]
3. (“School”) AND (“suspension” OR “expulsion” OR “exclusion”) AND (“interview” OR “qual*” OR “process” OR “evaluation”) [232 results]

**BMJ Controlled Trials**

**QUANT**

**Conducted:** 8/11/22

**Timeframe:** Oct/2015 – Nov/2022

**Total results:** 2

**Note:** results cannot be exported and imported to Covidence, screening done on the website.

**Saved:** 0

**Final searches string:**

1. school exclusion - in title or abstract and in full text (phrase)
2. school suspension - in title or abstract and in full text (phrase)
3. school expulsion - in title or abstract and in full text (phrase)

**QUAL**

**Conducted:** 16/09/23

**Timeframe:** Oct/2015 – Dec/2022

**Total results:** 35

**Note:** results cannot be exported and imported to Covidence, screening done on the website.

**Saved:** 0

**Final searches string:**

1. “school exclusion” AND “qualitative” AND “United Kingdom”

**CBCA Education (Canada)**

**Conducted:** 14/02/2023

**Timeframe:** 2015 –2022

**Total results:** 12

**Note:** results imported to Covidence

**Final searches string:**

| 1 | MAINSUBJECT.EXACT("Expulsions & suspensions") |
| --- | --- |
| 2 | TI,AB(expuls* OR expel* OR suspen* OR "stand down") |
| 3 | 1 or 2 |
| 4 | MAINSUBJECT.EXACT("Secondary schools") OR MAINSUBJECT.EXACT("Private schools") OR MAINSUBJECT.EXACT("Middle schools") OR MAINSUBJECT.EXACT("Elementary schools") OR MAINSUBJECT.EXACT("Public schools") |
| 5 | MAINSUBJECT.EXACT("Middle school students") OR MAINSUBJECT.EXACT("Secondary school students") OR MAINSUBJECT.EXACT("Elementary school students") |
| 6 | TI,AB(school or schools) |
| 7 | TI,AB((school* OR elementary OR primary OR "middle school*" OR "junior high" OR secondary OR "high school*" OR grade*) NEAR/4 (student* OR children)) |
| 8 | 4 or 5 or 6 or 7 |
| 9 | TI,AB(intervention* OR program* OR prevention* OR policy OR policies OR initiative* OR strateg*) |
| 10 | MAINSUBJECT.EXACT("Quantitative analysis") OR MAINSUBJECT.EXACT("Qualitative research") |
| 11 | TI,AB("randomi* control trial*" or rct or experiment* or "quasi experiment*" or impact* or effect* or efficacy or efficien* or evaluation* or assess* or qualitative or implement* or process*) |
| 12 | 10 or 11 |
| 13 | 3 and 8 and 9 and 12 |

**ClinicalTrials.gov**

**QUANT**

**Conducted:** 8/11/22

**Timeframe:** 01/Oct/2015 – 08/Nov/2022

**Total results:** 0

**Final searches string:**

1. "school exclusion"
2. "school suspension"
3. "school expulsion"

Also searched without using quotes, none were related.

**QUAL**

**Conducted:** 16/09/23

**Timeframe:** 01/Oct/2015 – 31/Dec/2022

**Total results:** 150

**Notes:** results could not be exported, hand searched. No relevant results.

**Final searches string:**

1. "school exclusion" [0 results]
2. "school suspension" [0 results]
3. "expulsion" [150 results]

**Criminal Justice Abstracts (via EBSCO)**

**QUANT**

**Search #1**

**Conducted:** 8/11/22

**Timeframe:** Oct/2015 – Nov/2022

**Total results:** 33

**Note:** results imported to Covidence

**Final searches string:**

((evaluation) OR (effectiveness) OR (program*) OR (impact) OR (effect) OR (experimental) OR (quasi-experimental) OR (RCT) or ("random evaluation") OR ("efficacy trial") OR ("process evaluation"))

AND (("disciplinary methods") OR ("token economy") OR ("classroom management") OR ("school management") OR ("early intervention") OR ("school support project") OR ("skills training"))

AND ((schoolchildren) OR (pupils) OR (children) OR (adolescents) OR ("school-aged children") OR (student) OR (youth) OR ("young people"))

AND (("school exclusion") OR ("suspension reduction") OR ("out-of-school suspension") OR ("in-school suspension") OR ("out-of-school exclusion") OR ("in-school exclusion") OR (suspended) OR (suspension) OR (expelled) OR (expulsion) OR ("outdoor suspension") OR (stand-down) OR ("exclusionary discipline") OR (discipline))

**Search #2**

**Conducted:** 22/11/22

**Timeframe:** Oct/2015 – Nov/2022

**Total results:** 113

**Final searches string:**

1. "school exclusion"
2. "school suspension"
3. "school expulsion"
4. school suspended
5. school expelled

**QUAL**

**Search #1**

**Conducted:** 16/09/23

**Timeframe:** Oct/2015 – Dec/2022

**Total results:** 1

**Final searches string:**

(“Effect” OR “Effectiveness” OR “Evaluation” OR “Impact” OR “Intervention” OR “Process Evaluation” OR “Programme” OR “Programme Effectiveness”) AND (“Classroom intervention” OR “Classroom Interventions” OR “Classroom management” OR “Classroom management programme” OR “Classroom Strategies” OR “Disciplinary methods” OR “Early intervention” OR “Early interventions” OR “School management” OR “School support projects” OR “Skills training OR Token economy”) AND (“Adolescent” OR “Adolescents” OR “Children” OR “Pupils” OR “School children” OR “School-aged children” OR “Student” OR “Students” OR “Young People” OR “Youth”) AND (“Access” OR “Barriers” OR “Discipline” OR “Exclusionary discipline” OR “Expelled” OR “Expulsion” OR “Facilitators” OR “Implementation” OR “In-school exclusion” OR “In-school suspension” OR “Out-of-school exclusion” OR “Out-of-school suspension” OR “Outdoor suspension” OR “School exclusion” OR “School exclusion reduction” OR “Stand-down” OR “Suspended” OR “Suspension” OR “Suspension reduction”) AND (“Ethnography” OR “Focus group” OR “Interview” OR “Qualitative” OR “Qualitative data”) AND (“United Kingdom”)

**Search #2**

**Conducted:** 16/09/23

**Timeframe:** Oct/2015 – Dec/2022

**Total results:** 1

**Final searches string:**

(“evaluation” OR “effectiveness” OR “intervention” OR “programme” OR “process evaluation” OR “impact” OR “effect” OR “process”) AND (“disciplinary methods” OR “token economy” OR “classroom management” OR “classroom intervention” OR “classroom strategies” OR “school management” OR “early intervention” OR “school support projects” OR “skills training”) AND (“school children” OR “pupils” OR “adolescent” OR “student” OR “youth” OR “young people” or “school-aged children”) AND (“school exclusion” OR “suspension” OR “suspended” OR “expelled” OR “expulsion” OR “outdoor suspension” OR “stand-down” OR “exclusionary discipline” OR “implementation” OR “facilitators” OR “access” OR “barriers”) AND (“qualitative” OR “qualitative data” OR “interviews” OR “focus groups” OR “ethnography”) AND (“United Kingdom”)

**Search #3**

**Conducted:** 16/09/23

**Timeframe:** Oct/2015 – Dec/2022

**Total results:** 2

**Final searches string:**

(“process evaluation” OR “intervention” OR “programme” OR “effectiveness”) AND (“token economy” OR “classroom management” OR “early intervention” OR “skills training”) AND (“pupils” OR “students” OR “youth” OR “adolescent” OR “children”) AND (“school exclusion” OR “expulsion” OR “expelled” OR “suspended” or “suspension” OR “discipline” OR “access” OR “barriers” OR “facilitators”) AND ("qualitative” OR “interview” OR “focus group” OR “ethnography”) AND (“United Kingdom”)

**Search #4**

**Conducted:** 16/09/23

**Timeframe:** Oct/2015 – Dec/2022

**Total results:** 5

**Final searches string:**

(“Qualitative” OR “process” OR “evaluation”) AND (“school exclusion” OR “school suspension” OR “expulsion" OR “suspension”) AND (“United Kingdom”)

**Cochrane Central Register of Controlled Trials (CENTRAL)**

**QUANT**

**Conducted**: 8/11/22

**Timeframe:** 2015 – 2022

**Total results:** 24

**Note:** results imported to Covidence

**Final searches string:**

((evaluation) OR (effectiveness) OR (program*) OR (impact) OR (effect) OR (experimental) OR ("quasi-experimental") OR (RCT) or ("random evaluation") OR ("efficacy trial") OR ("process evaluation"))

AND (("disciplinary methods") OR ("token economy") OR ("classroom management") OR ("school management") OR ("early intervention") OR ("school support project") OR ("skills training"))

AND ((schoolchildren) OR (pupils) OR (children) OR (adolescents) OR ("school-aged children") OR (student) OR (youth) OR ("young people"))

AND (("school exclusion") OR ("suspension reduction") OR ("out-of-school suspension") OR ("in-school suspension") OR ("out-of-school exclusion") OR ("in-school exclusion") OR (suspended) OR (suspension) OR (expelled) OR (expulsion) OR ("outdoor suspension") OR (stand-down) OR ("exclusionary discipline") OR (discipline))

**QUAL**

**Search #1**

**Conducted:** 16/09/23

**Timeframe:** Oct/2015 – Dec/2022

**Total results:** 11

**Final searches string:**

(“Effect” OR “Effectiveness” OR “Evaluation” OR “Impact” OR “Intervention” OR “Process Evaluation” OR “Programme” OR “Programme Effectiveness”) AND (“Classroom intervention” OR “Classroom Interventions” OR “Classroom management” OR “Classroom management programme” OR “Classroom Strategies” OR “Disciplinary methods” OR “Early intervention” OR “Early interventions” OR “School management” OR “School support projects” OR “Skills training OR Token economy”) AND (“Adolescent” OR “Adolescents” OR “Children” OR “Pupils” OR “School children” OR “School-aged children” OR “Student” OR “Students” OR “Young People” OR “Youth”) AND (“Access” OR “Barriers” OR “Discipline” OR “Exclusionary discipline” OR “Expelled” OR “Expulsion” OR “Facilitators” OR “Implementation” OR “In-school exclusion” OR “In-school suspension” OR “Out-of-school exclusion” OR “Out-of-school suspension” OR “Outdoor suspension” OR “School exclusion” OR “School exclusion reduction” OR “Stand-down” OR “Suspended” OR “Suspension” OR “Suspension reduction”) AND (“Ethnography” OR “Focus group” OR “Interview” OR “Qualitative” OR “Qualitative data”) AND (“United Kingdom”)

**Search #2**

**Conducted:** 16/09/23

**Timeframe:** Oct/2015 – Dec/2022

**Total results:** 14

**Final searches string:**

(“evaluation” OR “effectiveness” OR “intervention” OR “programme” OR “process evaluation” OR “impact” OR “effect” OR “process”) AND (“disciplinary methods” OR “token economy” OR “classroom management” OR “classroom intervention” OR “classroom strategies” OR “school management” OR “early intervention” OR “school support projects” OR “skills training”) AND (“school children” OR “pupils” OR “adolescent” OR “student” OR “youth” OR “young people” or “school-aged children”) AND (“school exclusion” OR “suspension” OR “suspended” OR “expelled” OR “expulsion” OR “outdoor suspension” OR “stand-down” OR “exclusionary discipline” OR “implementation” OR “facilitators” OR “access” OR “barriers”) AND (“qualitative” OR “qualitative data” OR “interviews” OR “focus groups” OR “ethnography”) AND (“United Kingdom”)

**Search #3**

**Conducted:** 16/09/23

**Timeframe:** Oct/2015 – Dec/2022

**Total results:** 26

**Final searches string:**

(“process evaluation” OR “intervention” OR “programme” OR “effectiveness”) AND (“token economy” OR “classroom management” OR “early intervention” OR “skills training”) AND (“pupils” OR “students” OR “youth” OR “adolescent” OR “children”) AND (“school exclusion” OR “expulsion” OR “expelled” OR “suspended” or “suspension” OR “discipline” OR “access” OR “barriers” OR “facilitators”) AND ("qualitative” OR “interview” OR “focus group” OR “ethnography”) AND (“United Kingdom”)

**Database of Abstracts of Reviews of Effects (DARE)**

**QUANT**

**Conducted:** 19/10/22

**Timeframe:** 2015 – 2022

**Total results:** 0

**Final searches string:**

1. ((evaluation) OR (effectiveness) OR (program*) OR (impact) OR (effect) OR (experimental) OR (quasi-experimental) OR (RCT) or (random evaluation) OR (efficacy trial) OR (process evaluation))

AND ((disciplinary methods) OR (token economy) OR (classroom management) OR (school management) OR (early intervention) OR (school support project) OR (skills training))

AND ((schoolchildren) OR (pupils) OR (children) OR (adolescents) OR (school-aged children) OR (student) OR (youth) OR (young people))

AND ((school exclusion) OR (suspension reduction) OR (out-of-school suspension) OR (in-school suspension) OR (out-of-school exclusion) OR (in-school exclusion) OR (suspend*) OR (expelled) OR (expulsion) OR (outdoor suspension) OR (stand-down) OR (exclusionary discipline) OR (discipline))

1. ("school exclusion") OR ("suspension reduction") OR ("out-of-school suspension") OR ("in-school suspension") OR ("out-of-school exclusion") OR ("in-school exclusion") OR (suspended) OR (suspension) OR (expelled) OR (expulsion) OR ("outdoor suspension") OR (stand-down) OR ("exclusionary discipline") OR (discipline)
2. School exclusion
3. School suspension
4. School expulsion

Same results using or not using quotation marks.

**QUAL**

**Search #1**

**Conducted:** 16/09/23

**Timeframe:** Oct/2015 – Dec/2022

**Total results:** 0

**Final searches string:**

(“Effect” OR “Effectiveness” OR “Evaluation” OR “Impact” OR “Intervention” OR “Process Evaluation” OR “Programme” OR “Programme Effectiveness”) AND (“Classroom intervention” OR “Classroom Interventions” OR “Classroom management” OR “Classroom management programme” OR “Classroom Strategies” OR “Disciplinary methods” OR “Early intervention” OR “Early interventions” OR “School management” OR “School support projects” OR “Skills training OR Token economy”) AND (“Adolescent” OR “Adolescents” OR “Children” OR “Pupils” OR “School children” OR “School-aged children” OR “Student” OR “Students” OR “Young People” OR “Youth”) AND (“Access” OR “Barriers” OR “Discipline” OR “Exclusionary discipline” OR “Expelled” OR “Expulsion” OR “Facilitators” OR “Implementation” OR “In-school exclusion” OR “In-school suspension” OR “Out-of-school exclusion” OR “Out-of-school suspension” OR “Outdoor suspension” OR “School exclusion” OR “School exclusion reduction” OR “Stand-down” OR “Suspended” OR “Suspension” OR “Suspension reduction”) AND (“Ethnography” OR “Focus group” OR “Interview” OR “Qualitative” OR “Qualitative data”) AND (“United Kingdom”)

**Search #2**

**Conducted:** 16/09/23

**Timeframe:** Oct/2015 – Dec/2022

**Total results:** 0

**Final searches string:**

(“evaluation” OR “effectiveness” OR “intervention” OR “programme” OR “process evaluation” OR “impact” OR “effect” OR “process”) AND (“disciplinary methods” OR “token economy” OR “classroom management” OR “classroom intervention” OR “classroom strategies” OR “school management” OR “early intervention” OR “school support projects” OR “skills training”) AND (“school children” OR “pupils” OR “adolescent” OR “student” OR “youth” OR “young people” or “school-aged children”) AND (“school exclusion” OR “suspension” OR “suspended” OR “expelled” OR “expulsion” OR “outdoor suspension” OR “stand-down” OR “exclusionary discipline” OR “implementation” OR “facilitators” OR “access” OR “barriers”) AND (“qualitative” OR “qualitative data” OR “interviews” OR “focus groups” OR “ethnography”) AND (“United Kingdom”)

**Search #3**

**Conducted:** 16/09/23

**Timeframe:** Oct/2015 – Dec/2022

**Total results:** 0

**Final searches string:**

(“process evaluation” OR “intervention” OR “programme” OR “effectiveness”) AND (“token economy” OR “classroom management” OR “early intervention” OR “skills training”) AND (“pupils” OR “students” OR “youth” OR “adolescent” OR “children”) AND (“school exclusion” OR “expulsion” OR “expelled” OR “suspended” or “suspension” OR “discipline” OR “access” OR “barriers” OR “facilitators”) AND ("qualitative” OR “interview” OR “focus group” OR “ethnography”) OR (“United Kingdom”)

**Search #4**

**Conducted:** 16/09/23

**Timeframe:** Oct/2015 – Dec/2022

**Total results:** 0

**Final searches string:**

1. “school exclusion”
2. “expulsion”
3. “suspended”
4. “suspension”

**Educational Resources Information Center – ERIC (via EBSCO)**

**QUANT**

**Search #1**

**Conducted:** 9/11/22

**Timeframe:** Oct/2015 – Nov/2022

**Total results:** 303

**Note:** results imported to Covidence

**Final searches string:**

((evaluation) OR (effectiveness) OR (program*) OR (impact) OR (effect) OR (experimental) OR (quasi-experimental) OR (RCT) or ("random evaluation") OR ("efficacy trial") OR ("process evaluation"))

AND (("disciplinary methods") OR ("token economy") OR ("classroom management") OR ("school management") OR ("early intervention") OR ("school support project") OR ("skills training"))

AND ((schoolchildren) OR (pupils) OR (children) OR (adolescents) OR ("school-aged children") OR (student) OR (youth) OR ("young people"))

AND (("school exclusion") OR ("suspension reduction") OR ("out-of-school suspension") OR ("in-school suspension") OR ("out-of-school exclusion") OR ("in-school exclusion") OR (suspended) OR (suspension) OR (expelled) OR (expulsion) OR ("outdoor suspension") OR (stand-down) OR ("exclusionary discipline") OR (discipline))

**Search #2**

**Conducted:** 22/11/22

**Timeframe:** Oct/2015 – Nov/2022

**Total results:** 875

**Note:** results imported to Covidence

**Final searches string:**

1. "school exclusion" AND rct
2. "school exclusion" AND quasi-experiment*
3. "school exclusion" AND evaluation
4. "school exclusion" AND impact
5. "school exclusion" AND effect
6. "school exclusion" AND program*
7. "school suspension" AND rct
8. "school suspension" AND quasi-experiment*
9. "school suspension" AND evaluation
10. "school suspension" AND impact
11. "school suspension" AND effect
12. "school suspension" AND program*
13. "school expulsion" AND rct
14. "school expulsion" AND quasi-experiment*
15. "school expulsion" AND evaluation
16. "school expulsion" AND impact
17. "school expulsion" AND effect
18. "school expulsion" AND program*

**Search #3**

**Conducted:** 17/02/2023

**Timeframe:** Oct/2015 – Nov/2022

**Total results:** 287

**Note:** results imported to Covidence

**Final searches string:**

| 1 | MAINSUBJECT.EXACT("Suspension") OR MAINSUBJECT.EXACT("Zero Tolerance Policy") OR MAINSUBJECT.EXACT("Expulsion") |
| --- | --- |
| 2 | TI,AB(expuls* OR expel* OR suspen* OR "stand down") |
| 3 | 1 or 2 |
| 4 | MAINSUBJECT.EXACT("Schools") OR MAINSUBJECT.EXACT("Middle Schools") OR MAINSUBJECT.EXACT("Private Schools") OR MAINSUBJECT.EXACT("Junior High Schools") OR MAINSUBJECT.EXACT("High Schools") OR MAINSUBJECT.EXACT("Public Schools") OR MAINSUBJECT.EXACT("Elementary Schools") OR MAINSUBJECT.EXACT("Secondary Schools") |
| 5 | MAINSUBJECT.EXACT("High School Students") OR MAINSUBJECT.EXACT("Junior High School Students") OR MAINSUBJECT.EXACT("Middle School Students") OR MAINSUBJECT.EXACT("Secondary School Students") OR MAINSUBJECT.EXACT("Elementary School Students") |
| 6 | TI,AB(school or schools) |
| 7 | TI,AB((school* OR elementary OR primary OR "middle school*" OR "junior high" OR secondary OR "high school*" OR grade*) NEAR/4 (student* OR children)) |
| 8 | 4 or 5 or 6 or 7 |
| 9 | TI,AB(intervention* OR program* OR prevention* OR policy OR policies OR initiative* OR strateg*) |
| 10 | MAINSUBJECT.EXACT("Randomized Controlled Trials") |
| 11 | TI,AB("randomi* control trial*" or rct or experiment* or "quasi experiment*" or impact* or effect* or efficacy or efficien*) |
| 12 | 10 or 11 |
| 13 | 3 and 8 and 9 and 12 |

Search was initially done on both the ERIC website and through EBSCO, EBSCO generated more results and thus those were kept.

**QUAL**

**Search #1**

**Conducted:** 16/09/23

**Timeframe:** 01/Oct/2015 – 08/Nov/2022

**Total results:** 1,258

**Final searches string:**

(“School exclusion” OR “School suspension” OR “exclusion” OR “suspension”) AND (“qual*” OR “process” OR “interview”)

**Search #2**

**Conducted:** 16/09/23

**Timeframe:** 01/Oct/2015 – 08/Nov/2022

**Total results:** 132

**Final searches string:**

(“School exclusion” OR “School suspension” OR “exclusion” OR “suspension”) AND (“qual*” OR “process” OR “interview”) AND ("united kingdom")

**Search #3**

**Conducted:** 16/09/23

**Timeframe:** 01/Oct/2015 – 08/Nov/2022

**Total results:** 134

**Final searches string:**

("process evaluation") OR ("school exclusion") AND ("qualitative") AND ("united kingdom")

**Search #4**

**Conducted:** 16/09/23

**Timeframe:** 01/Oct/2015 – 08/Nov/2022

**Total results:** 14

**Final searches string:**

(“Effect” OR “Effectiveness” OR “Evaluation” OR “Impact” OR “Intervention” OR “Process Evaluation” OR “Programme” OR “Programme Effectiveness”) AND (“Classroom intervention” OR “Classroom Interventions” OR “Classroom management” OR “Classroom management programme” OR “Classroom Strategies” OR “Disciplinary methods” OR “Early intervention” OR “Early interventions” OR “School management” OR “School support projects” OR “Skills training OR Token economy”) AND (“Adolescent” OR “Adolescents” OR “Children” OR “Pupils” OR “School children” OR “School-aged children” OR “Student” OR “Students” OR “Young People” OR “Youth”) AND (“Access” OR “Barriers” OR “Discipline” OR “Exclusionary discipline” OR “Expelled” OR “Expulsion” OR “Facilitators” OR “Implementation” OR “In-school exclusion” OR “In-school suspension” OR “Out-of-school exclusion” OR “Out-of-school suspension” OR “Outdoor suspension” OR “School exclusion” OR “School exclusion reduction” OR “Stand-down” OR “Suspended” OR “Suspension” OR “Suspension reduction”) AND (“Ethnography” OR “Focus group” OR “Interview” OR “Qualitative” OR “Qualitative data”) AND (“United Kingdom”)

**EMBASE (via Ovid)**

**QUANT**

**Search #1**

**Conducted:** 19/10/22

**Timeframe:** 2015 – 2022

**Total results:** 101

**Note:** results imported to Covidence

**Final searches string:** 52

((evaluation) OR (effectiveness) OR (program*) OR (impact) OR (effect) OR (experimental) OR (quasi-experimental) OR (RCT) or ("random evaluation") OR ("efficacy trial") OR ("process evaluation"))

AND (("disciplinary methods") OR ("token economy") OR ("classroom management") OR ("school management") OR ("early intervention") OR ("school support project") OR ("skills training"))

AND ((schoolchildren) OR (pupils) OR (children) OR (adolescents) OR ("school-aged children") OR (student) OR (youth) OR ("young people"))

AND (("school exclusion") OR ("suspension reduction") OR ("out-of-school suspension") OR ("in-school suspension") OR ("out-of-school exclusion") OR ("in-school exclusion") OR (suspended) OR (suspension) OR (expelled) OR (expulsion) OR ("outdoor suspension") OR (stand-down) OR ("exclusionary discipline") OR (discipline))

**Search #2**

**Conducted:** 22/11/22

**Timeframe:** 2015 – 2022

**Total results:** 101

**Note:** results imported to Covidence

**Final searches string:**

1. ("school exclusion" OR "school suspension") AND (rct OR experiments OR quasi-experiments OR impact OR intervention OR program*)
2. "school exclusion" AND rct
3. "school exclusion" AND quasi-experiment*
4. "school exclusion" AND evaluation
5. "school exclusion" AND impact
6. "school exclusion" AND effect
7. "school exclusion" AND program*
8. "school suspension" AND rct
9. "school suspension" AND quasi-experiment*
10. "school suspension" AND evaluation
11. "school suspension" AND impact
12. "school suspension" AND effect
13. "school suspension" AND program*
14. "school expulsion" AND rct
15. "school expulsion" AND quasi-experiment*
16. "school expulsion" AND evaluation
17. "school expulsion" AND impact
18. "school expulsion" AND effect
19. "school expulsion" AND program*

**QUAL**

**Search #1**

**Conducted:** 16/09/23

**Timeframe:** 2015 – 2022

**Total results:** 18

**Final searches string:**

(Effect OR Effectiveness OR Evaluation OR Impact OR Intervention OR Process Evaluation OR Programme OR Programme Effectiveness) AND (Classroom intervention OR Classroom Interventions OR Classroom management OR Classroom management programme OR Classroom Strategies OR Disciplinary methods OR Early intervention OR Early interventions OR School management OR School support projects OR Skills training OR Token economy) AND (Adolescent OR Adolescents OR Children OR Pupils OR School children OR School-aged children OR Student OR Students OR Young People OR Youth) AND (Access OR Barriers OR Discipline OR Exclusionary discipline OR Expelled OR Expulsion OR Facilitators OR Implementation OR In-school exclusion OR In-school suspension OR Out-of-school exclusion OR Out-of-school suspension OR Outdoor suspension OR School exclusion OR School exclusion reduction OR Stand-down OR Suspended OR Suspension OR Suspension reduction) AND (Ethnography OR Focus group OR Interview OR Qualitative OR Qualitative data) AND (United Kingdom)

**Search #2**

**Conducted:** 16/09/23

**Timeframe:** 2015 – 2022

**Total results:** 24

**Final searches string:**

(process evaluation OR intervention OR programme OR effectiveness) AND (token economy OR classroom management OR early intervention OR skills training) AND (pupils OR students OR youth OR adolescent OR children) AND (school exclusion OR expulsion OR expelled OR suspended or suspension OR discipline OR access OR barriers OR facilitators) AND (qualitative OR interview OR focus group OR ethnography) AND (United Kingdom)

**Search #3**

**Conducted:** 16/09/23

**Timeframe:** 2015 – 2022

**Total results:** 2,432

**Final searches string:**

("school exclusion") AND ("qualitative") AND ("united kingdom")

**Search #4**

**Conducted:** 16/09/23

**Timeframe:** 2015 – 2022

**Total results:** 2,144

**Final searches string:**

("school suspension") AND ("qualitative") AND ("united kingdom")

**EThOS**

**QUANT**

**Conducted:** 3/11/22

**Timeframe:** 2015 – 2022

**Total results:** 66

**Note:** results cannot be exported and imported to Covidence, screening done separately.

**Saved**: 2 (systematic reviews to check references: Alabbad et al., 2020; Hindmarch, 2017 – both already in Covidence)

Final searches string:

1. “school exclusion”
2. “school suspension”
3. “school expulsion”

**QUAL**

**Search #1**

**Conducted:** 16/09/23

**Timeframe:** 2015 – 2022 (cannot filter by publication date)

**Total results:** 1 (hand searched, irrelevant – published in 2013)

**Final searches string:**

**Search #2**

**Conducted:** 16/09/23

**Timeframe:** 2015 – 2022 (cannot filter by publication date)

**Total results:** 1 (hand searched, irrelevant – published in 2013)

**Final searches string:**

("school exclusion") AND ("qualitative") AND ("united kingdom")

**Search #3**

**Conducted:** 17/09/23

**Timeframe:** 2015 – 2022 (cannot filter by publication date)

**Total results:** 14 (hand searched – 1 relevant and retained)

**Final searches string:**

(“intervention” OR “classroom intervention” OR “effect”) AND “pupils” AND “interviews”

**Additional searches**

**Conducted:** 16/09/23

**Timeframe:** 2015 – 2022 (cannot filter by publication date)

**Total results:** 0

**Final searches string:**

1. “School exclusion” AND “qual*”
2. “School suspension” AND “qual*”
3. (“School exclusion” OR “School suspension” OR “exclusion” OR “suspension”) AND (“qual*” OR “process” OR “interview”)

**Google Scholar**

**QUANT**

**Conducted:** 8/11/22

**Timeframe:** 2015 – 2022

**Total results:** 986

**Note:** results imported to Covidence

**Final searches string:**

("school exclusion" OR "school suspension" OR "school expulsion") AND (impact OR rct OR quasi experiment) AND (children OR students) AND ("disciplinary methods" OR token economy OR "classroom management" OR "school management" OR intervention)

**QUAL**

**Search #1**

**Conducted:** 16/09/23

**Timeframe:** 2015 – 2022 (cannot sort by date)

**Total results:** 967

**Final searches string:**

("school exclusion" OR "suspension" OR "expulsion") AND (qualitative or "interview") AND (children OR students) AND ("disciplinary methods" OR token economy OR "classroom management" OR "school management" OR intervention) AND ("United Kingdom")

**Search #2**

**Conducted:** 17/09/23

**Timeframe:** 2015 – 2022 (cannot sort by date)

**Total results:** 560

**Final searches string:**

("pupils") AND ("school exclusion") AND ("United Kingdom") AND ("qualitative")

**Institute of Education Sciences – What Works Clearinghouse**

**QUANT**

**Conducted:** 7/11/22

**Timeframe**: cannot be filtered by date

**Total results:** 192 (any publication date)

**Notes:** references cannot be exported, not uploaded to Covidence. Screening done separately.

**Saved:** 0 (studies found had been already identified through other databases or did not meet criteria)

**Final searches string:**

1. school exclusion (design: rct)
2. school exclusion (design: quasi-experimental)
3. school suspension (design: rct)
4. school suspension (design: quasi-experimental)
5. school expulsion (design: rct)
6. school expulsion (design: quasi-experimental)

**QUAL**

**Conducted:** 16/09/23

**Timeframe:** 2015 – 2022 (cannot sort by date)

**Total results:** 3

**Notes:** hand-searched, could not be exported. 0 relevant.

**Final searches string:**

1. school exclusion AND qualitative AND United Kingdom
2. School exclusion
3. Expulsion [3 results]

**ISI Web of Science**

**QUANT**

**Search #1**

**Conducted:** 8/11/22

**Timeframe:**

**Total results:** 291

**Note:** results imported to Covidence

**Final searches string:**

((evaluation) OR (effectiveness) OR (program*) OR (impact) OR (effect) OR (experimental) OR (quasi-experimental) OR (RCT) or ("random evaluation") OR ("efficacy trial") OR ("process evaluation"))

AND (("disciplinary methods") OR ("token economy") OR ("classroom management") OR ("school management") OR ("early intervention") OR ("school support project") OR ("skills training"))

AND ((schoolchildren) OR (pupils) OR (children) OR (adolescents) OR ("school-aged children") OR (student) OR (youth) OR ("young people"))

AND (("school exclusion") OR ("suspension reduction") OR ("out-of-school suspension") OR ("in-school suspension") OR ("out-of-school exclusion") OR ("in-school exclusion") OR (suspended) OR (suspension) OR (expelled) OR (expulsion) OR ("outdoor suspension") OR (stand-down) OR ("exclusionary discipline") OR (discipline))

**Search #2**

**Conducted:** 11/11/22

**Timeframe:** 01/Oct/2015 – 08/Nov/2022

**Total results:** 157

**Note:** results imported to Covidence

**Final searches string:**

"school exclusion" or "school suspension" AND rct or experiments or quasi-experiments or impact or intervention or program

**Search #3**

**Conducted:** 22/11/22

**Timeframe:** 01/Oct/2015 – 22/Nov/2022

**Total results:** 1,241

**Note:** results imported to Covidence

**Final searches string:**

1. (experiment*) OR (evaluation) OR (random*) OR (intervention) OR (effective*) OR (efficacy) OR (quasi) OR (impact) OR (RCT)

AND (school) AND (*exclusion)

1. (school) AND (*suspension*)

AND (experiment*) OR (evaluation) OR (random*) OR (intervention) OR (effective*) OR (efficacy) OR (quasi) OR (impact) OR (RCT)

1. (school) AND (suspended)

AND (experiment*) OR (evaluation) OR (random*) OR (intervention) OR (effective*) OR (efficacy) OR (quasi) OR (impact) OR (RCT)

**Refined by research area:** Refined by: Psychology or Family Studies Or Education Educational Research Or Behavioral Sciences Or Psychiatry Or Criminology Penology Or Sociology Or Ethnic Studies Or Social Work Or Urban Studies Or Social Sciences Other Topics Or Social Issues

**QUAL**

**Search #1**

**Conducted:** 16/09/23

**Timeframe:** 1/Oct/2015 – 31/Dec/2022

**Total results:** 10

**Final searches string:**

ALL=((“Effect” OR “Effectiveness” OR “Evaluation” OR “Impact” OR “Intervention” OR “Process Evaluation” OR “Programme” OR “Programme Effectiveness”) AND (“Classroom intervention” OR “Classroom Interventions” OR “Classroom management” OR “Classroom management programme” OR “Classroom Strategies” OR “Disciplinary methods” OR “Early intervention” OR “Early interventions” OR “School management” OR “School support projects” OR “Skills training OR Token economy”) AND (“Adolescent” OR “Adolescents” OR “Children” OR “Pupils” OR “School children” OR “School-aged children” OR “Student” OR “Students” OR “Young People” OR “Youth”) AND (“Access” OR “Barriers” OR “Discipline” OR “Exclusionary discipline” OR “Expelled” OR “Expulsion” OR “Facilitators” OR “Implementation” OR “Outdoor suspension” OR “School exclusion” OR “School exclusion reduction” OR “Stand-down” OR “Suspended” OR “Suspension” OR “Suspension reduction”) AND (“Ethnography” OR “Focus group” OR “Interview” OR “Qualitative” OR “Qualitative data”) AND (“United Kingdom”))

**Search #2**

**Conducted:** 16/09/23

**Timeframe:** 1/Oct/2015 – 31/Dec/2022

**Total results:** 7

**Final searches string:**

(“process evaluation” OR “intervention” OR “programme” OR “effectiveness”) AND (“token economy” OR “classroom management” OR “early intervention” OR “skills training”) AND (“pupils” OR “students” OR “youth” OR “adolescent” OR “children”) AND (“school exclusion” OR “expulsion” OR “expelled” OR “suspended” or “suspension” OR “discipline” OR “access” OR “barriers” OR “facilitators”) AND ("qualitative” OR “interview” OR “focus group” OR “ethnography”) AND (“United Kingdom”)

**Search #3**

**Conducted:** 16/09/23

**Timeframe:** 1/Oct/2015 – 31/Dec/2022

**Total results:** 7

**Final searches string:**

(“evaluation” OR “effectiveness” OR “intervention” OR “programme” OR “process evaluation” OR “impact” OR “effect” OR “process”) AND (“disciplinary methods” OR “token economy” OR “classroom management” OR “classroom intervention” OR “classroom strategies” OR “school management” OR “early intervention” OR “school support projects” OR “skills training”) AND (“school children” OR “pupils” OR “adolescent” OR “student” OR “youth” OR “young people” or “school-aged children”) AND (“school exclusion” OR “suspension” OR “suspended” OR “expelled” OR “expulsion” OR “outdoor suspension” OR “stand-down” OR “exclusionary discipline” OR “implementation” OR “facilitators” OR “access” OR “barriers”) AND (“qualitative” OR “qualitative data” OR “interviews” OR “focus groups” OR “ethnography”) AND (“United Kingdom”)

**Search #4**

**Conducted:** 16/09/23

**Timeframe:** 1/Oct/2015 – 31/Dec/2022

**Total results:** 8

**Final searches string:**

1. (TS =(“school exclusion” OR “school suspension” OR “school expulsion”)) AND (TS=(“evaluation” OR “process” OR “qual*” OR “interview” OR “focus group”)) AND (TS=(“United Kingdom”)) [1 result]
2. (TS =(“school”)) AND (TS=(“qualitative”)) AND (TS=(“exclusion” OR “suspension” OR expulsion”)) AND (TS=(“United Kingdom”)) [7 results]

**MEDLINE (via PubMed)**

**QUANT**

**Conducted:** 8/11/22

**Timeframe:** 01/Oct/2015 – 08/Nov/2022

**Total results:** 142

**Note:** results imported to Covidence

**Final searches string:**

((evaluation) OR (effectiveness) OR (program*) OR (impact) OR (effect) OR (experimental) OR (quasi-experimental) OR (RCT) or ("random evaluation") OR ("efficacy trial") OR ("process evaluation")) AND (("disciplinary methods") OR ("token economy") OR ("classroom management") OR ("school management") OR ("early intervention") OR ("school support project") OR ("skills training")) AND ((schoolchildren) OR (pupils) OR (children) OR (adolescents) OR ("school-aged children") OR (student) OR (youth) OR ("young people")) AND (("school exclusion") OR ("suspension reduction") OR ("out-of-school suspension") OR ("in-school suspension") OR ("out-of-school exclusion") OR ("in-school exclusion") OR (suspended) OR (suspension) OR (expelled) OR (expulsion) OR ("outdoor suspension") OR (stand-down) OR ("exclusionary discipline") OR (discipline))

**QUAL**

**Search #1**

**Conducted:** 16/09/23

**Timeframe:** 1/Oct/2015 – 31/Dec/2022

**Total results:** 127

**Final searches string:**

(“Effect” OR “Effectiveness” OR “Evaluation” OR “Impact” OR “Intervention” OR “Process Evaluation” OR “Programme” OR “Programme Effectiveness”) AND (“Classroom intervention” OR “Classroom Interventions” OR “Classroom management” OR “Classroom management programme” OR “Classroom Strategies” OR “Disciplinary methods” OR “Early intervention” OR “Early interventions” OR “School management” OR “School support projects” OR “Skills training OR Token economy”) AND (“Adolescent” OR “Adolescents” OR “Children” OR “Pupils” OR “School children” OR “School-aged children” OR “Student” OR “Students” OR “Young People” OR “Youth”) AND (“Access” OR “Barriers” OR “Discipline” OR “Exclusionary discipline” OR “Expelled” OR “Expulsion” OR “Facilitators” OR “Implementation” OR “In-school exclusion” OR “In-school suspension” OR “Out-of-school exclusion” OR “Out-of-school suspension” OR “Outdoor suspension” OR “School exclusion” OR “School exclusion reduction” OR “Stand-down” OR “Suspended” OR “Suspension” OR “Suspension reduction”) AND (“Ethnography” OR “Focus group” OR “Interview” OR “Qualitative” OR “Qualitative data”) AND (“United Kingdom”)

**Search #2**

**Conducted:** 16/09/23

**Timeframe:** 1/Oct/2015 – 31/Dec/2022

**Total results:** 30

**Final searches string:**

(“evaluation” OR “effectiveness” OR “intervention” OR “programme” OR “process evaluation” OR “impact” OR “effect” OR “process”) AND (“disciplinary methods” OR “token economy” OR “classroom management” OR “classroom intervention” OR “classroom strategies” OR “school management” OR “early intervention” OR “school support projects” OR “skills training”) AND (“school children” OR “pupils” OR “adolescent” OR “student” OR “youth” OR “young people” or “school-aged children”) AND (“school exclusion” OR “suspension” OR “suspended” OR “expelled” OR “expulsion” OR “outdoor suspension” OR “stand-down” OR “exclusionary discipline” OR “implementation” OR “facilitators” OR “access” OR “barriers”) AND (“qualitative” OR “qualitative data” OR “interviews” OR “focus groups” OR “ethnography”) AND (“United Kingdom”)

**Search #3**

**Conducted:** 16/09/23

**Timeframe:** 1/Oct/2015 – 31/Dec/2022

**Total results:** 339

**Final searches string:**

(“process evaluation” OR “intervention” OR “programme” OR “effectiveness”) AND (“token economy” OR “classroom management” OR “early intervention” OR “skills training”) AND (“pupils” OR “students” OR “youth” OR “adolescent” OR “children”) AND (“school exclusion” OR “expulsion” OR “expelled” OR “suspended” or “suspension” OR “discipline” OR “access” OR “barriers” OR “facilitators”) AND ("qualitative” OR “interview” OR “focus group” OR “ethnography”) AND (“United Kingdom”)

**ProQuest Dissertations & Theses A&I**

**QUANT**

**Conducted:** 11/11/22

**Timeframe:** 2015 – 2022

**Total results:** 3,717

**Note:** results imported to Covidence

**Final searches string:**

("school exclusion" OR "school suspension" OR "school expulsion") AND (impact OR rct OR quasi experiment) AND ("disciplinary methods" OR "classroom management" OR "school management" OR intervention)

**Exclude**: (individual & family studies AND disability studies AND womens studies AND public policy AND public health AND perceptions AND instructional design AND economics AND lgbtq studies AND personality psychology AND adult education AND public administration AND demography AND law AND mental disorders AND qualitative research AND cultural anthropology AND education finance AND families & family life AND parents & parenting AND political science AND community college education AND ethics)

**QUAL**

**Conducted:** 16/09/23

**Timeframe:** 1/Oct/2015 – 31/Dec/2022

**Total results:** 3,080

**Final searches string:**

(“Effect” OR “Effectiveness” OR “Evaluation” OR “Impact” OR “Intervention” OR “Process Evaluation” OR “Programme” OR “Programme Effectiveness”) AND (“Classroom intervention” OR “Classroom Interventions” OR “Classroom management” OR “Classroom management programme” OR “Classroom Strategies” OR “Disciplinary methods” OR “Early intervention” OR “Early interventions” OR “School management” OR “School support projects” OR “Skills training OR Token economy”) AND (“Adolescent” OR “Adolescents” OR “Children” OR “Pupils” OR “School children” OR “School-aged children” OR “Student” OR “Students” OR “Young People” OR “Youth”) AND (“Access” OR “Barriers” OR “Discipline” OR “Exclusionary discipline” OR “Expelled” OR “Expulsion” OR “Facilitators” OR “Implementation” OR “In-school exclusion” OR “In-school suspension” OR “Out-of-school exclusion” OR “Out-of-school suspension” OR “Outdoor suspension” OR “School exclusion” OR “School exclusion reduction” OR “Stand-down” OR “Suspended” OR “Suspension” OR “Suspension reduction”) AND (“Ethnography” OR “Focus group” OR “Interview” OR “Qualitative” OR “Qualitative data”) AND (“United Kingdom”)

**PsycInfo (via EBSCO)**

**QUANT**

**Search #1**

**Conducted:** 8/11/22

**Timeframe:** Oct/2015 – Nov/2022

**Total results:** 345

**Note:** results imported to Covidence

**Final searches string:**

((evaluation) OR (effectiveness) OR (program*) OR (impact) OR (effect) OR (experimental) OR (quasi-experimental) OR (RCT) or ("random evaluation") OR ("efficacy trial") OR ("process evaluation"))

AND (("disciplinary methods") OR ("token economy") OR ("classroom management") OR ("school management") OR ("early intervention") OR ("school support project") OR ("skills training"))

AND ((schoolchildren) OR (pupils) OR (children) OR (adolescents) OR ("school-aged children") OR (student) OR (youth) OR ("young people"))

AND (("school exclusion") OR ("suspension reduction") OR ("out-of-school suspension") OR ("in-school suspension") OR ("out-of-school exclusion") OR ("in-school exclusion") OR (suspended) OR (suspension) OR (expelled) OR (expulsion) OR ("outdoor suspension") OR (stand-down) OR ("exclusionary discipline") OR (discipline))

**Search #2**

**Conducted:** 22/11/22

**Timeframe:** Oct/2015 – Nov/2022

**Total results:** 861

**Note:** results imported to Covidence

**Final searches string:**

1. ("school exclusion" OR "school suspension" OR "school expulsion") AND (rct OR experiments OR quasi-experiments OR impact OR intervention OR program* OR effect)
2. "school exclusion"
3. "school suspension"
4. "school expulsion"
5. school suspended
6. school expelled

**QUAL:**

**Search #1**

**Conducted:** 16/09/23

**Timeframe:** 1/Oct/2015 – 31/Dec/2022

**Total results:** 34

(“Effect” OR “Effectiveness” OR “Evaluation” OR “Impact” OR “Intervention” OR “Process Evaluation” OR “Programme” OR “Programme Effectiveness”) AND (“Classroom intervention” OR “Classroom Interventions” OR “Classroom management” OR “Classroom management programme” OR “Classroom Strategies” OR “Disciplinary methods” OR “Early intervention” OR “Early interventions” OR “School management” OR “School support projects” OR “Skills training OR Token economy”) AND (“Adolescent” OR “Adolescents” OR “Children” OR “Pupils” OR “School children” OR “School-aged children” OR “Student” OR “Students” OR “Young People” OR “Youth”) AND (“Access” OR “Barriers” OR “Discipline” OR “Exclusionary discipline” OR “Expelled” OR “Expulsion” OR “Facilitators” OR “Implementation” OR “In-school exclusion” OR “In-school suspension” OR “Out-of-school exclusion” OR “Out-of-school suspension” OR “Outdoor suspension” OR “School exclusion” OR “School exclusion reduction” OR “Stand-down” OR “Suspended” OR “Suspension” OR “Suspension reduction”) AND (“Ethnography” OR “Focus group” OR “Interview” OR “Qualitative” OR “Qualitative data”) AND (“United Kingdom”)

**Search #2**

**Conducted:** 16/09/23

**Timeframe:** 1/Oct/2015 – 31/Dec/2022

**Total results:** 105

(“evaluation” OR “effectiveness” OR “intervention” OR “programme” OR “process evaluation” OR “impact” OR “effect” OR “process”) AND (“disciplinary methods” OR “token economy” OR “classroom management” OR “classroom intervention” OR “classroom strategies” OR “school management” OR “early intervention” OR “school support projects” OR “skills training”) AND (“school children” OR “pupils” OR “adolescent” OR “student” OR “youth” OR “young people” or “school-aged children”) AND (“school exclusion” OR “suspension” OR “suspended” OR “expelled” OR “expulsion” OR “outdoor suspension” OR “stand-down” OR “exclusionary discipline” OR “implementation” OR “facilitators” OR “access” OR “barriers”) AND (“qualitative” OR “qualitative data” OR “interviews” OR “focus groups” OR “ethnography”) AND (“United Kingdom”)

**Search #3**

**Conducted:** 16/09/23

**Timeframe:** 1/Oct/2015 – 31/Dec/2022

**Total results:** 47

(“process evaluation” OR “intervention” OR “programme” OR “effectiveness”) AND (“token economy” OR “classroom management” OR “early intervention” OR “skills training”) AND (“pupils” OR “students” OR “youth” OR “adolescent” OR “children”) AND (“school exclusion” OR “expulsion” OR “expelled” OR “suspended” or “suspension” OR “discipline” OR “access” OR “barriers” OR “facilitators”) AND ("qualitative” OR “interview” OR “focus group” OR “ethnography”) AND (“United Kingdom”)

**Scientific Electronic Library Online (SciELO) (via ISI Web of Science)**

**QUANT**

**Conducted**: 8/11/22

**Timeframe**: 1/Oct/2015 – 08/Nov/2022

**Total results**: 3

**Note:** results imported to Covidence

**Final searches string:**

((evaluation) OR (effectiveness) OR (program*) OR (impact) OR (effect) OR (experimental) OR (quasi-experimental) OR (RCT) or ("random evaluation") OR ("efficacy trial") OR ("process evaluation"))

AND (("disciplinary methods") OR ("token economy") OR ("classroom management") OR ("school management") OR ("early intervention") OR ("school support project") OR ("skills training"))

AND ((schoolchildren) OR (pupils) OR (children) OR (adolescents) OR ("school-aged children") OR (student) OR (youth) OR ("young people"))

AND (("school exclusion") OR ("suspension reduction") OR ("out-of-school suspension") OR ("in-school suspension") OR ("out-of-school exclusion") OR ("in-school exclusion") OR (suspended) OR (suspension) OR (expelled) OR (expulsion) OR ("outdoor suspension") OR (stand-down) OR ("exclusionary discipline") OR (discipline))

**QUAL**

**Conducted:** 16/09/23

**Timeframe:** 1/Oct/2015 – 31/Dec/2022

**Total results:** 7

**Final searches string:**

(“process evaluation” OR “intervention” OR “programme” OR “effectiveness”) AND (“token economy” OR “classroom management” OR “early intervention” OR “skills training”) AND (“pupils” OR “students” OR “youth” OR “adolescent” OR “children”) AND (“school exclusion” OR “expulsion” OR “expelled” OR “suspended” or “suspension” OR “discipline” OR “access” OR “barriers” OR “facilitators”) AND ("qualitative” OR “interview” OR “focus group” OR “ethnography”) AND (“United Kingdom”)

**Science.gov**

**QUANT**

**Conducted:** 14/11/22

**Timeframe:** 2015 –2022

**Total results**: 82

**Note:** results imported to Covidence

**Final searches string:**

1. "school suspension" AND evaluation
2. "school suspension" AND experiment*
3. "school exclusion" AND evaluation
4. "school exclusion" AND experiment*
5. "school expulsion" AND evaluation
6. "school expulsion" AND experiment*

**QUAL**

**Search #1**

**Conducted:** 16/09/23

**Timeframe:** 1/Oct/2015 – 31/Dec/2022

**Total results:** 132

**Final searches string:**

(“Effect” OR “Effectiveness” OR “Evaluation” OR “Impact” OR “Intervention” OR “Process Evaluation” OR “Programme” OR “Programme Effectiveness”) AND (“Classroom intervention” OR “Classroom Interventions” OR “Classroom management” OR “Classroom management programme” OR “Classroom Strategies” OR “Disciplinary methods” OR “Early intervention” OR “Early interventions” OR “School management” OR “School support projects” OR “Skills training OR Token economy”) AND (“Adolescent” OR “Adolescents” OR “Children” OR “Pupils” OR “School children” OR “School-aged children” OR “Student” OR “Students” OR “Young People” OR “Youth”) AND (“Access” OR “Barriers” OR “Discipline” OR “Exclusionary discipline” OR “Expelled” OR “Expulsion” OR “Facilitators” OR “Implementation” OR “In-school exclusion” OR “In-school suspension” OR “Out-of-school exclusion” OR “Out-of-school suspension” OR “Outdoor suspension” OR “School exclusion” OR “School exclusion reduction” OR “Stand-down” OR “Suspended” OR “Suspension” OR “Suspension reduction”) AND (“Ethnography” OR “Focus group” OR “Interview” OR “Qualitative” OR “Qualitative data”) AND (“United Kingdom”)

**Search #2**

**Conducted:** 16/09/23

**Timeframe:** 1/Oct/2015 – 31/Dec/2022

**Total results:** 221

**Final searches string:**

(“evaluation” OR “effectiveness” OR “intervention” OR “programme” OR “process evaluation” OR “impact” OR “effect” OR “process”) AND (“disciplinary methods” OR “token economy” OR “classroom management” OR “classroom intervention” OR “classroom strategies” OR “school management” OR “early intervention” OR “school support projects” OR “skills training”) AND (“school children” OR “pupils” OR “adolescent” OR “student” OR “youth” OR “young people” or “school-aged children”) AND (“school exclusion” OR “suspension” OR “suspended” OR “expelled” OR “expulsion” OR “outdoor suspension” OR “stand-down” OR “exclusionary discipline” OR “implementation” OR “facilitators” OR “access” OR “barriers”) AND (“qualitative” OR “qualitative data” OR “interviews” OR “focus groups” OR “ethnography”) AND (“United Kingdom”)

**Search #3**

**Conducted:** 16/09/23

**Timeframe:** 1/Oct/2015 – 31/Dec/2022

**Total results:** 212

**Final searches string:**

(process evaluation OR intervention OR programme OR effectiveness) AND (token economy OR classroom management OR early intervention OR skills training) AND (pupils OR students OR youth OR adolescent OR children) AND (school exclusion OR expulsion OR expelled OR suspended or suspension OR discipline OR access OR barriers OR facilitators) AND (qualitative OR interview OR focus group OR ethnography) AND (United Kingdom)

**Sociological Abstracts (via ProQuest)**

**QUANT**

**Conducted:** 9/11/22

**Timeframe:** 1/Oct/2015 – 08/Nov/2022

**Total results:** 1,144

**Note:** results imported to Covidence

**Final searches string:**

((evaluation) OR (effectiveness) OR (program*) OR (impact) OR (effect) OR (experimental) OR (quasi-experimental) OR (RCT) or ("random evaluation") OR ("efficacy trial") OR ("process evaluation"))

AND (("disciplinary methods") OR ("token economy") OR ("classroom management") OR ("school management") OR ("early intervention") OR ("school support project") OR ("skills training"))

AND ((schoolchildren) OR (pupils) OR (children) OR (adolescents) OR ("school-aged children") OR (student) OR (youth) OR ("young people"))

AND (("school exclusion") OR ("suspension reduction") OR ("out-of-school suspension") OR ("in-school suspension") OR ("out-of-school exclusion") OR ("in-school exclusion") OR (suspended) OR (suspension) OR (expelled) OR (expulsion) OR ("outdoor suspension") OR (stand-down) OR ("exclusionary discipline") OR (discipline))

**QUAL**

**Search #1**

**Conducted:** 16/09/23

**Timeframe:** 1/Oct/2015 – 31/Dec/2022

**Total results:** 351

**Final searches string:**

(“process evaluation” OR “intervention” OR “programme” OR “effectiveness”) AND (“token economy” OR “classroom management” OR “early intervention” OR “skills training”) AND (“pupils” OR “students” OR “youth” OR “adolescent” OR “children”) AND (“school exclusion” OR “expulsion” OR “expelled” OR “suspended” or “suspension” OR “discipline” OR “access” OR “barriers” OR “facilitators”) AND ("qualitative” OR “interview” OR “focus group” OR “ethnography”) AND (“United Kingdom”)

**Search #2**

**Conducted:** 16/09/23

**Timeframe:** 1/Oct/2015 – 31/Dec/2022

**Total results:** 391

**Final searches string:**

(“evaluation” OR “effectiveness” OR “intervention” OR “programme” OR “process evaluation” OR “impact” OR “effect” OR “process”) AND (“disciplinary methods” OR “token economy” OR “classroom management” OR “classroom intervention” OR “classroom strategies” OR “school management” OR “early intervention” OR “school support projects” OR “skills training”) AND (“school children” OR “pupils” OR “adolescent” OR “student” OR “youth” OR “young people” or “school-aged children”) AND (“school exclusion” OR “suspension” OR “suspended” OR “expelled” OR “expulsion” OR “outdoor suspension” OR “stand-down” OR “exclusionary discipline” OR “implementation” OR “facilitators” OR “access” OR “barriers”) AND (“qualitative” OR “qualitative data” OR “interviews” OR “focus groups” OR “ethnography”) AND (“United Kingdom”)

**Search #3**

**Conducted:** 16/09/23

**Timeframe:** 1/Oct/2015 – 31/Dec/2022

**Total results:** 315

**Final searches string:**

(“Effect” OR “Effectiveness” OR “Evaluation” OR “Impact” OR “Intervention” OR “Process Evaluation” OR “Programme” OR “Programme Effectiveness”) AND (“Classroom intervention” OR “Classroom Interventions” OR “Classroom management” OR “Classroom management programme” OR “Classroom Strategies” OR “Disciplinary methods” OR “Early intervention” OR “Early interventions” OR “School management” OR “School support projects” OR “Skills training OR Token economy”) AND (“Adolescent” OR “Adolescents” OR “Children” OR “Pupils” OR “School children” OR “School-aged children” OR “Student” OR “Students” OR “Young People” OR “Youth”) AND (“Access” OR “Barriers” OR “Discipline” OR “Exclusionary discipline” OR “Expelled” OR “Expulsion” OR “Facilitators” OR “Implementation” OR “In-school exclusion” OR “In-school suspension” OR “Out-of-school exclusion” OR “Out-of-school suspension” OR “Outdoor suspension” OR “School exclusion” OR “School exclusion reduction” OR “Stand-down” OR “Suspended” OR “Suspension” OR “Suspension reduction”) AND (“Ethnography” OR “Focus group” OR “Interview” OR “Qualitative” OR “Qualitative data”) AND (“United Kingdom”)

**Social Sciences Citation Index (SSCI) (via ISI Web of Science)**

**QUANT**

**Conducted:** 8/11/22

**Timeframe:** 1/Oct/2015 – 08/Nov/2022

**Total results:** 196

**Note:** results imported to Covidence

**Final searches string:**

((evaluation) OR (effectiveness) OR (program*) OR (impact) OR (effect) OR (experimental) OR (quasi-experimental) OR (RCT) or ("random evaluation") OR ("efficacy trial") OR ("process evaluation"))

AND (("disciplinary methods") OR ("token economy") OR ("classroom management") OR ("school management") OR ("early intervention") OR ("school support project") OR ("skills training"))

AND ((schoolchildren) OR (pupils) OR (children) OR (adolescents) OR ("school-aged children") OR (student) OR (youth) OR ("young people"))

AND (("school exclusion") OR ("suspension reduction") OR ("out-of-school suspension") OR ("in-school suspension") OR ("out-of-school exclusion") OR ("in-school exclusion") OR (suspended) OR (suspension) OR (expelled) OR (expulsion) OR ("outdoor suspension") OR (stand-down) OR ("exclusionary discipline") OR (discipline))

**QUAL**

**Search #1**

**Conducted:** 16/09/23

**Timeframe:** 1/Oct/2015 – 31/Dec/2022

**Total results:** 6

**Final searches string:**

(“evaluation” OR “effectiveness” OR “intervention” OR “programme” OR “process evaluation” OR “impact” OR “effect” OR “process”) AND (“disciplinary methods” OR “token economy” OR “classroom management” OR “classroom intervention” OR “classroom strategies” OR “school management” OR “early intervention” OR “school support projects” OR “skills training”) AND (“school children” OR “pupils” OR “adolescent” OR “student” OR “youth” OR “young people” or “school-aged children”) AND (“school exclusion” OR “suspension” OR “suspended” OR “expelled” OR “expulsion” OR “outdoor suspension” OR “stand-down” OR “exclusionary discipline” OR “implementation” OR “facilitators” OR “access” OR “barriers”) AND (“qualitative” OR “qualitative data” OR “interviews” OR “focus groups” OR “ethnography”) AND (“United Kingdom”)

**Search #2**

**Conducted:** 16/09/23

**Timeframe:** 1/Oct/2015 – 31/Dec/2022

**Total results:** 6

**Final searches string:**

(“process evaluation” OR “intervention” OR “programme” OR “effectiveness”) AND (“token economy” OR “classroom management” OR “early intervention” OR “skills training”) AND (“pupils” OR “students” OR “youth” OR “adolescent” OR “children”) AND (“school exclusion” OR “expulsion” OR “expelled” OR “suspended” or “suspension” OR “discipline” OR “access” OR “barriers” OR “facilitators”) AND ("qualitative” OR “interview” OR “focus group” OR “ethnography”) AND (“United Kingdom”)

**Search #3**

**Conducted:** 17/09/23

**Timeframe:** 1/Oct/2015 – 31/Dec/2022

**Total results:** 1

**Final searches string:**

("school exclusion" OR "suspension" OR "expulsion") AND ("qualitative data" OR "interview" OR "focus group") AND ("United Kingdom")

**The National Dropout Prevention Centre Network – Hand search**

**QUANT & QUAL**

**Conducted:** 11/11/22

**Timeframe:** cannot filter by date

**Total results:** 13 reports (10 from 2015-22)

**Note:** searched on section “Papers, reports, and books.” Results cannot be exported and imported to Covidence, screening done separately.

**Saved:** 0

**Campbell Collaboration Social, Psychological, Educational and Criminological Trials Register (C2-SPECTR) – Hand search**

**QUANT**

**Conducted:** 11/11/22

**Total results:** 44

**Note:** searched on section “Research evidence.” Results cannot be exported and imported to Covidence, screening done separately.

**Saved:** 0

**Final searches string:**

1. school exclusion OR school suspension OR school expulsion
2. school dropout
3. disciplinary
4. discipline
5. school

**QUAL**

**Conducted:** 16/09/23

**Timeframe:** 1/Oct/2015 – 31/Dec/2022

**Total results:** 0 [handsearched]

**Final searches string:**

“school exclusion” AND “qualitative “AND “united kingdom”

**WHO- International Clinical Trials Registry Platform (ICTRP)**

**QUANT**

**Conducted:** 19/10/22

**Timeframe:** cannot filter by date

**Total results:** 9

**Note:** results cannot be exported and imported to Covidence, screening done separately.

**Saved:** 0

**Final searches string:**

1. ((evaluation) OR (effectiveness) OR (program*) OR (impact) OR (effect) OR (experimental) OR (quasi-experimental) OR (RCT) or (random evaluation) OR (efficacy trial) OR (process evaluation))

AND ((disciplinary methods) OR (token economy) OR (classroom management) OR (school management) OR (early intervention) OR (school support project) OR (skills training))

AND ((schoolchildren) OR (pupils) OR (children) OR (adolescents) OR (school-aged children) OR (student) OR (youth) OR (young people))

AND ((school exclusion) OR (suspension reduction) OR (out-of-school suspension) OR (in-school suspension) OR (out-of-school exclusion) OR (in-school exclusion) OR (suspend*) OR (expelled) OR (expulsion) OR (outdoor suspension) OR (stand-down) OR (exclusionary discipline) OR (discipline))

**Filter**: with results only

1. School exclusion
2. School suspension
3. School expulsion

**QUAL**

**Conducted:** 18/09/23

**Timeframe:** cannot filter by date

**Total results:** 1

**Note:** results cannot be exported and imported to Covidence, screening done separately.

**Saved:** 0

**Final searches string:**

1. (“process evaluation” OR “intervention” OR “programme” OR “effectiveness”) AND (“token economy” OR “classroom management” OR “early intervention” OR “skills training”) AND (“pupils” OR “students” OR “youth” OR “adolescent” OR “children”) AND (“school exclusion” OR “expulsion” OR “expelled” OR “suspended” or “suspension” OR “discipline” OR “access” OR “barriers” OR “facilitators”) AND ("qualitative” OR “interview” OR “focus group” OR “ethnography”) AND (“United Kingdom”) [1 result]
2. ("qualitative") AND ("school exclusion") AND ("united kingdom") [0 results]

**APPENDIX B: Sources hand searched**

*List of reviews, books, policy briefs, and reports included in hand searches*

| **Author(s)** | **Type** | **Number of references checked** | **New references added to review** |
| --- | --- | --- | --- |
| Alabbad et al. (2020) | SR | 9 | MacIver et al. (2016) |
| Alegre (2018) | Review | 5 | 0 |
| Amores-Valencia et al. (2022) | SR | 0 | 0 |
| Ankele (2022) | Review | 0 | 0 |
| Armstrong (2021) |  | 1 | 0 |
| Aydin et al. (2021) | meta-analysis | 0 | 0 |
| Bal (2018) | SR | 3 | 0 |
| Barker & De Lugt (2022) | SR | 3 | 0 |
| Barker et al. (2022) | Book | 3 | Lee & Gage (2020) |
| Bausback (2021) | meta-analysis | 1 | 0 |
| Bernier et al. (2021) | Lit review | 0 | 0 |
| Bireda (2019) | Book | 0 | 0 |
| Bohnenkamp et al. (2022) | Review | 1 | Bohnenkamp et al. (2021) |
| Bradshaw (2015) | Review | 0 | 0 |
| Brown, C. et al. (2018) | SR | 1 | 0 |
| Bruhn & McDaniel (2021) |  | 7 | 0 |
| Camman & Wormith (2015) | Lit review | 0 | 0 |
| Chávez et al. (2021) | Working paper | 2 | 0 |
| Cheang et al. (2019) | SR | 0 | 0 |
| Cho et al. (2020) | SR | 8 | 0 |
| Counts et al. (2018) | Review | 0 | 0 |
| Cruz et al. (2021) | synthesis | 8 | 0 |
| De Oliveira et al. (2022) | SR | 0 | 0 |
| Diclemente (2021) | SR | 10 | Bohnenkamp, 2021 |
| Dreger & Downey (2022) | meta-analysis | 0 | 0 |
| Egeberg et al. (2016) | Lit review | 0 | 0 |
| Ekstrand (2015) | Review | 1 | 0 |
| Evans et al. (2017) | SR | 0 | 0 |
| Felty (2021) | Lit review | 0 | 0 |
| Fenning & Johnson (2018) | Book chapter | 0 | 0 |
| Freeman et al. (2018) | SR | 0 | 0 |
| Freiberg et al. (2020) | meta review | 2 | 0 |
| Fronius et al. (2019) | Review | 2 | 0 |
| Fronius et al. (2016) | Review | 0 | 0 |
| Gadd & Butler (2019) | annotated bibliography | 1 | 0 |
| Gaffney et al. (2022) | Review | 1 | 0 |
| Gaffney et al. (2021) | SR, meta-analysis | 0 | 0 |
| Gage et al. (2018) | SR, meta-analysis | 0 | 0 |
| Gage et al. (2020) |  | 2 | 0 |
| Gavine et al. (2016) | SR | 0 | 0 |
| Gion et al. (2020) | Chapter of book | 1 Book | 0 |
| González et al. (2019) |  | 0 | 0 |
| Gregory & Evans (2020) | Policy brief | 2 | 0 |
| Hakvoort et al. (2022) | bibliometric review | 2 | 0 |
| Hassani et al. (2022) | SR | 0 | 0 |
| Hepburn & Beamish (2019) | SR | 4 | 0 |
| Herrenkohl et al. | Review | 3 | 0 |
| Hicks (2021) | SR | 0 | 0 |
| Hindmarch (2017) | SR | 0 | 0 |
| Hokanson (2021) | SR | 0 | 0 |
| Jean-Pierre & Parris-Drummond (2018) | Lit review | 6 | 0 |
| John W. Gardner Center for Youth (2020) |  | 0 | 0 |
| Kearney & Graczyk (2020) |  | 8 | 0 |
| Keen et al. (2017) | SR | 0 | 0 |
| Kern et al. (2020) | Book chapter | 0 | 0 |
| Kincade et al. (2020) | meta-analysis | 2 |  |
| Komer (2020) | Thesis | 2 |  |
| Larson (2016) | SR | 0 | 0 |
| Lloyd et al. (2019) | SR | 0 | 0 |
| Lodi et al. (2021) | SR | 1 | 0 |
| Macias (2018) | Review | 0 | 0 |
| McGuire et al. (2021) | SR | 0 | 0 |
| Mckeering & Hwang (2019) | SR | 0 | 0 |
| McNeill et al. (2016) | Lit review | 0 | 0 |
| Medina (2020) | Thesis | 1 | 0 |
| Messeter & Soni (2018) | SR | 5 | 0 |
| Mielke & Farrington (2021) | meta-analysis | 5 | 0 |
| Miley (2019) | Lit review | 0 | 0 |
| Monroe (2015) | meta-analysis | 0 | 0 |
| Moore et al. (2019) | Review | 1 | 0 |
| Moreno (2021) |  | 1 |  |
| Morgan (2021) | SR | 0 | 0 |
| Morrish et al. (2018) |  | 1 | 0 |
| National Association Of School Psychologists (NASP; 2020) | Report | 0 | 0 |
| Nese & McIntosh (2016) | Book chapter | 0 | 0 |
| Nickerson (2019) |  | 0 | 0 |
| O’Reilly et al. (2018) | SR | 0 | 0 |
| Olubiyi et al. (2019) | SR | 1 | 0 |
| Paramita et al. (2020) | SR | 0 | 0 |
| Pastor-Porras & Suelves (2021) | lit review | 0 | 0 |
| Patnode et al. (2018) | lit review | 3 | 0 |
| Perry (2016) |  | 0 | 0 |
| Quail & Ward (2022) | SR | 0 | 0 |
| Regnier et al. (2022) | SR | 0 | 0 |
| Rivara & Le Menestrel (2016) | Book | 0 | 0 |
| Roseby & Gascoigne (2021) | SR | 2 | 0 |
| Roth (2016) | Book | 0 | 0 |
| Salas-Rodriguez & Lara (2020) |  | 0 | 0 |
| Samuel (2018) |  | 0 | 0 |
| Sanchez et al. (2021) |  | 0 | 0 |
| Schiff (2018) |  | 0 | 0 |
| Severini et al. (2018) | SR | 0 | 0 |
| Shakeel et al. (2016) | SR, meta-analysis | 0 | 0 |
| Sivaraman et al. (2019) | SR | 0 | 0 |
| Skiba et al. (2016) |  | 0 | 0 |
| Starzecki (2022) | SR | 0 | 0 |
| Stone (2018) | meta-analysis | 0 | 0 |
| Turner (2019) | meta-analysis | 0 | 0 |
| Valdebenito et al. (2019) | SR, meta-analysis | 0 | 0 |
| van der Meulen et al. (2021) | SR | 0 | 0 |
| van Loon et al. (2020) | meta-analysis | 0 | 0 |
| Welsh & Little (2018) | review | 2 | Cornell & Lovegrove, 2015 (2013) |
| Woods & Stewart (2018) |  | 0 | 0 |
| Yeung et al. (2016) | Lit review | 0 | 0 |
| Zinsser et al. (2022) | SR | 1 | 0 |

*Note.* SR = Systematic review

## APPENDIX C. SCREENING TOOLS

**Quantitative studies (RCTs and QEDs)**

| **Criteria** | **Evaluation** | |
| --- | --- | --- |
| 1. Does this paper measure school exclusion as an outcome? | - YES | - NO |
| Does the intervention is school based? (or at least one component in the school) | - YES | - NO |
| 1. Are the target individuals school students in mainstream schools? | - YES | - NO |
| 1. Is the report based on an experimental design (RCT)? (if the answer is NO, go to the next item (Item 5, below) | - YES | - NO |
| 1. Is this report based on a QED as per our target?   The study uses before and after measures. Yes/No  The study uses at least one matched control group. Yes/No  The study match treatment and control group using demographics and at least one behavioural risk factor (.e.g., suspensions, violence, disciplinary problems, school absenteeism) Yes/No  The matching procedure produces balance between the treatment and control group Yes/No  **If one of the answers is NO, the study is not our target. Select NO in the last column 🡪 | - YES | - NO |
| Is this report included?  *(If you have selected NO in one or more of the previous questions the study will need to be excluded)* | - YES | - NO |
| Reasons for exclusion: | | |

**Qualitative Studies**

| **Criteria (PerSpecTIF)** | **Evaluation** | |
| --- | --- | --- |
| *Perspective: (i.e., the sample)*  Does the paper present findings from the perspective of children and young people, or school staff/teachers/school leaders? | - Yes | - No |
| *Setting:*  Does the paper present findings from a UK study? | - Yes | - No |
| *Phenomenon:*  Does the paper present findings of a process evaluation of an intervention? | - Yes | - No |
| *Environment:*  Does the paper present findings with school students in mainstream schools? | - Yes | - No |
| *Time:*  Does the process evaluation present findings at the end of an intervention or during an intervention? | - Yes | - No |
| *Findings:*  Does the report present findings on school exclusion/suspension? | \| - Yes \|  \| \| --- \| --- \| | - No |
| **Is this paper included?** | \| - Yes \|  \| \| --- \| --- \| | - No |
| Reasons for exclusion: | | |

## APPENDIX D: DATA COLLECTION INSTRUMENT (Quantitative studies)

**DATA-CODING INSTRUMENT**

***School-based interventions for reducing***

***disciplinary school exclusion. A systematic review***

[Variable names in brackets]

Contents

Section A. Codification

Section B. Bibliographical information

Section C. Ethics

Section D. Research design

Section E. Sample

Section F. Primary outcome coding

Section G. Secondary outcomes coding

Section H. Base-line measurements

Section I. Programme delivered

Section J. Follow-up measurement

Section K. Effect sizes

Eligibility checklist

**Section A. Codification**

Instruction: use one data-coding instrument for each manuscript. When more than one manuscript reports the same research project, select one of them as the principal (e.g., the older) and give it an ID number. The following manuscripts should use the same ID but it must be registered in the Crossref field.

**[STUDYID]** Study ID number:

**[CROSSREF1]** Cross reference document identifier:

**[CROSSREF2]** Cross reference document identifier:

**[CROSSREF3]** Cross reference document identifier:

**[DATESCR]** Date of screening:

**[CODER]** Coder Initials:

**Section B. Bibliographical information**

Before completing this section, please be sure that the manuscript is correctly uploaded in the reference manager programme.

**[AUTHOR]** Name of the main author(s):

**[AFFIL]** Main author affiliation:

**[DATEPUB]** Year of publication:

**[DATEFIEDW]** Year of fieldwork (usually reported in a range):

**[COISTATEMENT]** Has the paper included a conflict of interest statement?

- 1. Yes
- 0. No

**[LANGPUB]** Language of the publication:

- 1. English
- 2. German
- 3. Italian
- 4. Spanish
- 5. Portuguese
- 999. Other:________

**[COUNTPUB]** Country of publication:

- 1. UK
- 2. USA
- 3. Canada
- 4. Australia
- 999. Other:_________
- 99. Unknown

**[TYPUB]** Type of publication:

- 1. Journal
- 2. Book/book chapter
- 3. Masters thesis
- 4. PhD/doctoral thesis
- 5. Technical/governmental report
- 6. Conference proceedings
- 999. Other:________

**[AUTDIS]** Main author discipline:

- 1. Education
- 2. Social Work
- 3. Psychology
- 4. Criminal Justice
- 5. Sociology
- 6. Psychiatry/Medicine
- 999. Other:________
- 99. Unknown

**[LOCAT]** How was the study/report located?

- 1. Electronic database
- 2. Web search
- 3. Reference in a book/paper. Please specify:
- 4. Hand search in specialised journal
- 5. Peer/expert suggestion
- 999. Other. Specify:____________

**Section C. Ethics**

**[CONSENT]** Did the study declare the use of “consent agreement forms”?

- 1. Yes
- 0. No
- 999. Other:________
- 99. Unknown

**[SIGNCONS]** Who signed the consent?

- 1. Students
- 2. Parents
- 3. Teachers
- 4. Schools
- 5. Parents and student
- 999. Other. Specify:______________
- 99. Unknown

**Section D. Design**

The present systematic review includes randomised control trials as well as quasi-experimental reports (before/after measure plus a control or comparison group). If the control/comparison group is randomly allocated, non-randomly allocated or matched and no intervention expected to produce impact is provided to it, you will be able to code that group as CONTROL. Subsequently, the TREATMENT group could be understood as the group that receives the intervention, no matter if that condition has been randomly allocated or not.

Please select always the data that is related with the sample effectively analysed.

**[DESTYPE]** What kind of design is this paper based on?

- 1. Randomised controlled trial (true experiment)
- 2. Before-and-after with control/comparison group/s
- 3. Instrumental variable
- 4. Propensity score matching
- 5. Interrupted time series
- 6. Pre/post measures with unmatched control/comparison group
- 7. Inverse probability weighting
- 999. Other. Specify:____________

**[RANDUNIT]** Units of randomization

- 1. Individuals
- 2. Clusters/groups (classroom, schools)
- 999. Other. Specify:____________
- 99. Unknown

**[ANALUNIT]** Unit of analysis

- 1. Students
- 2. Clusters/groups (classroom, schools)
- 999. Other. Specify:____________
- 99. Unknown

**[COMPVAR]** Variables measured to create comparability? (e.g., variables used to match the control and treatment groups)

______________________________________________________________________________________________________________________________

**[MAINSTAT]** What is the main statistical analysis used to produce the final results?

- 1. Multilevel modelling
- 2. Differences of means
- 3. MANOVA
- 4. Chi-squared
- 5. Propensity Score Matching
- 999. Other. Specify:____________

**Section D. Sample**

**[SAMPSELECT]** How was the sample selected?

- 1. Randomly
- 2. Assessment
- 3. Self-selection
- 999. Other. Specify:___________

**[INSAMP]** Initial sample size (i.e., individuals/schools):

**[NUMBFOLL]** Nº of follow-up:

**[FOLLSAMP1]** Follow-up 1 sample size:

**[FOLLSAMP2]** Follow-up 2 sample size:

**[FOLLSAMP3]** Follow-up 3 sample size:

**[NSCHOOL]** Initial number of schools:

**[NSFOLL1]** Follow-up 1 sample size:

**[NSFOLL2]** Follow-up 2 sample size:

**[NSFOLL3]** Follow-up 3 sample size:

**[NCLASS]** Initial number of classes:

**[NCFOLL1]** Follow-up 1 sample size:

**[NCFOLL2]** Follow-up 2 sample size:

**[NCFOLL3]** Follow-up 3 sample size:

Please code here the information on attrition described in the manuscript:

|  | Total number  of students at  Baseline | Total number of students at Follow-up |
| --- | --- | --- |
| Treatment | **[NTREBAS]** | **[NTREFOLL]** |
| Control | **[NCONTBA]** | **[NCONTFOL]** |

**[MEANAGE]** Mean age and standard deviation of overall sample at beginning of intervention:

**[GENDER]** Gender

- % of males
- % of females
- 99. Unknown
- **[LOCAT]** Location of program
- Urban area
- Suburban area
- Rural area
- Mixture of areas
- 99. Not enough information to determine

**[GRADEX]** Grade level of students

- % of students in Elementary school or equivalent
- % of students in Secondary school or equivalent
- % of students in High school or equivalent
- 4. Other:
- 99. Unknown

**[ETHNI]** Predominant ethnicity^^[[1]](#footnote-1)^^

- 1. % of Caucasian:
- 2. % of Black:
- 3. % of Hispanic:
- 4. % of Asian:
- 5. % of other mixed background:
- 99. Unknown

**[COUNTRY]** Please state the name of the country where schools and sample of students were located when tested.

______________________ (99 if unknown)

**[LUNEX]** Socio-economic status

% of students receiving free/reduced school lunch:

99. Unknown

**[SENEX]** Special Educational Needs

% of students declaring SEN:

99. Unknown

**Section E. Primary Outcome (School Exclusion)**

**[EXCLUSION] Is the manuscript reporting outcomes for school exclusion?**

- 1. Yes
- 0. No

**[TYPEXC]** Type of exclusion measured

- 1. In-school exclusion
- 2. Out-of-school exclusion
- 99. Unknown

**[CHEKTIP]** Duration of school exclusion measured

- 1. Days of Fixed-term exclusion

(Expressed in number or days, frequencies, percentages)

- 2. Days of Permanent exclusion

(Expressed in number or days, frequencies, percentages)

- 99. Unknown

**[ICCEXCLU]** If the statistical analysis include cluster in MLM, please register the ICC for Exclusion:

**Section F. Secondary outcomes^^[[2]](#footnote-2)^^**

**[BEHAVMES]** Did the study include measures on behaviour domains?

- 1. Yes
- 0. No
- 99. Unknown

What types of the following behaviours are measured?

- **[PROSO]** Pro-social behaviour (e.g., helping, empathy). Specify:____________
- 1. Yes
- 0. No

**[MEPROSO]** Measure(s) used to test the behaviour (name):

**[ALPHAPROSO]**

- Reliability test. Specify alpha value:_____________
- Non reported

| Groups | Effect size before | Effect size after |
| --- | --- | --- |
| Control or comparison | **[PROBC]** | **[PROAC]** |
| Treatment | **[PROBT]** | **[PROAT]** |

**[PAGEPROSO]** Number of the page from where you extract statistical data:

**[ICCPROSO]** If the statistical analysis include cluster in MLM, please register the ICC for behavioural outcomes:

- **[INTERNAL]** Internalising problem behaviour

(e.g., anxiety, depression, attention-deficit and hyperactivity disorder (ADHD), attention deficit, hyperactivity). Specify:____________

- 1. Yes
- 0. No

**[MINTERNAL]** Measure (s) used to test the behaviour (name):

**[ALPHAINTERNAL]**

- Reliability test. Specify alpha value:_____________
- Non reported

| Groups | Effect size before | Effect size after |
| --- | --- | --- |
| Control or comparison | **[PROBC]** | **[PROAC]** |
| Treatment | **[PROBT]** | **[PROAT]** |

**[PAGEINTERNAL]** Number of the page from where you extract statistical data:

**[ICCINTERNAL]** If the statistical analysis include cluster in MLM, please register the ICC for behavioural outcomes:

- **[NAEXTERNAL]** Non-aggressive externalising problem behaviour

(e.g., stealing, lying, graffiti, illegal drugs). Specify: _____________

- 1. Yes
- 0. No

**[MNAEXTERNAL]** Measure used to test the behaviour (name):

**[ALPHANAEXTER]**

- Reliability test. Specify alpha value:_____________
- Non reported

| Groups | Effect size before | Effect size after |
| --- | --- | --- |
| Control or comparison | **[PROBC]** | **[PROAC]** |
| Treatment | **[PROBT]** | **[PROAT]** |

**[PAGENAXTERN]** Number of the page from where you extract statistical data:

**[ICCNAEXT]** If the statistical analysis include cluster in MLM, please register the ICC for behavioural outcomes:

- **[AAGRESEXT]** Aggressive externalising problem behaviour

(e.g., Opposition/defiance, physical aggression, indirect aggression, instrumental aggressions/dominance, reactive aggression, school bullying). Specify:_____________

- 1. Yes
- 0. No

**[MAGRESSEXT]** Measure used to test the behaviour (name):

**[ALPHAAEXT]**

- Reliability test. Specify alpha value:_____________
- Non reported

| Groups | Effect size before | Effect size after |
| --- | --- | --- |
| Control or comparison | **[PROBC]** | **[PROAC]** |
| Treatment | **[PROBT]** | **[PROAT]** |

**[AGRESPAGE]** Number of the page from where you extract statistical data:

**[ICCAGREEX]** If the statistical analysis include cluster in MLM, please register the ICC for Behavioural outcomes:

**Section G. Base-line measurements**

**[DATABAS]** Date of baseline assessment:

What measures were used?

**[SRMES]** Self-report

- 1. Yes
- 0. No
- 99. Unknown

**[TRMES]** Teachers’ report

- 1. Yes
- 0. No
- 99. Unknown

**[SCHRMES]** School records

- 1. Yes
- 0. No
- 99. Unknown

**[PAREP]** Parents

- 1. Yes
- 0. No
- 99. Unknown

**[OMES]** Other:_________________

**[EXCBL]** Frequency of exclusion at baseline (register any measure given by the study)

**Section H. Programme delivered**

This section aims to codify data on the delivery process. Be aware that sometimes final reports do not describe all the data related to delivery. In those cases it would be helpful to search for registered protocols or earlier publications reporting more data on this.

**[PRONAME]** Name of the programme:

**[PROCURRI]** Was the program curricular?

- 1. Yes
- 0. No
- 99. Unknown
- 999. Other. Specify:____________

**[PROEND]** The programme was conducted for:

- 1. Research ends
- 2. Demonstration ends
- 3. Routine
- 99. Unknown
- 999. Other. Specify:_____________

**[PROSIT]** Primary programme site:

- 1. Public school
- 2. Private school
- 3. Other, (specify):_________
- 99. Unknown

**[PROSCH]** Was at least one of the components of the intervention was settled at school?

- 1. Yes
- 0. No
- 99. Unknown

**[PRODEL]** Who delivered the programme?

- 1. External facilitators
- 2. School facilitators
- 3. Both
- 99. Unknown

**[PDBACK]** Deliverer’s background 1

- 1. Social worker
- 2. Psychologist
- 3. Teacher
- 4. Police officers
- 5. Peers
- 999. Other. Specify:_____________
- 99. Unknown

**[PDBACK]** Deliverer’s background 2

- 1. Social worker
- 2. Psychologist
- 3. Teacher
- 4. Police officers
- 5. Peers
- 999. Other. Specify:_____________
- 99. Unknown

**[TRAINBEF]** Did the deliverer receive training BEFORE implementing the programme?

- 1. Yes.
- 0. No.
- 99. Unknown

**[THOURS]** How long was the training in hours?:____________

**[TRAINDUR]** Did the deliverer receive training DURING the implementation?

- 1. Yes.
- 0. No.
- 99. Unknown

**[THOURS2]** How long was the training in hours?:

What type of intervention was delivered? If the manuscript indicates a mixture of interventions you can select more than one using TYPEPRO 1, 2 and 3.

|  | **[TYPEPRO1]** | **[TYPEPRO2]** | **[TYPEPRO3]** |
| --- | --- | --- | --- |
| 1. Mentoring programme |  |  |  |
| 2. Restorative programme |  |  |  |
| 3. Skills training programme |  |  |  |
| 4. School-wide systemic intervention |  |  |  |
| 5. Classroom management |  |  |  |
| 6. Counselling/therapy |  |  |  |
| 999. Other |  |  |  |

Theoretical background of the intervention. If the manuscript indicates a mixture of theories, you can select more than one using THEORY 1, 2 and 3.

|  | **[THEORY1]** | **[THEORY2]** | **[THEORY3]** |
| --- | --- | --- | --- |
| 1. Cognitive behavioural |  |  |  |
| 2. Learning theory |  |  |  |
| 3. Restorative theories |  |  |  |
| 4. Organisational theories or principles |  |  |  |
| 99. Unknown |  |  |  |
| 999. Other (Specify) |  |  |  |

**[PROCONT]** What happened to the control group?

- 1. No intervention
- 2. Wait-list control
- 3. Minimal contact
- 4. Treatment as usual
- 5. Alternative treatment
- 5. Placebo
- 999. Other. Specify:____________

**[PROFORM]** Delivery format:

- 1. Manualised programme
- 2. Unstructured programme
- 3. Mixed
- 99. Unknown
- 999. Other. Specify:____________

What was the programme dosage?

**[PRODOSW]** AVERAGE Duration in weeks:

**[PRODOSH]** AVERAGE Hours per week:

**[PROFREQ]** What was the frequency of the programme counted?

- 1. Less than a week
- 2. Once a week
- 3. Twice a week
- 4. 3-4 times a week
- 5. Daily
- 99. Unknown

**[EVROLE]** What was the “evaluator” role?

- 1. Deliver the programme
- 2. Designed the programme
- 3. Both design and delivery
- 4. Independent evaluator
- 99. Unknown

**[MONITOR]** Was the programme implementation monitored?

- 1. Yes
- 0. No
- 99. Unknown. Not enough information

**[IMPROB]** Does the report provide information about implementation problems?

- 1. Yes, there were clear problems which are reported
- 0. No, non-reported problems, reasonably well implemented
- 2. Possible problems based on the description of the intervention
- 99. Unknown. Not enough information

**[PROCOST]** Is the cost of the intervention mentioned?

- 1. Yes
- 0. No

**[AMOUNT]** Cost:

**[UNITCURR]** Currency:

**Section I. Follow-up measurement**

**[DATEFALL]** Date of follow up:

Multiple follow-ups

**[MONTHFO1]** Nº of months from baseline to 1^st^ follow-up:

**[MONTHFO2]** Nº of months from baseline to 2^nd^ follow-up:

**[MONTHFO3]** Nº of months from baseline to 3^rd^ follow-up:

**[MONTHFO4]** Nº of months from baseline to 4^th^ follow-up:

What measures were used?

**[POSTSR]** Children/adolescent self-report

- 1. Yes
- 0. No

**[POSTTR]** Teachers’ report

- 1. Yes
- 0. No

**[POSTSR]** School records

- 1. Yes
- 0. No

**[POSTPR]** Parents report

- 1. Yes
- 0. No

**[POSTO]** Other:__________

**[FREQEXFOLL]** Frequency of exclusion at follow-up (register any measure given by the study)

**Section J. Effect sizes of intervention on school exclusion**

- Effect size: outcomes expressed in continuous data.

**[CSSEX]** Sample size for the ES (Treatment group)

**[CSSCON]** Sample size for the ES (Control group)

**[MEANEX]** Mean (Treatment group)

**[MEANCON]** Mean (Control group)

**[MEANADJ]** Are the Means adjusted?

- 1. Yes.
- 0. No

**[ADJBY]** Adjusted by (describe):______________

**[SDEX]** Standard deviation (Treatment group)

**[SDCON]** Standard deviation (Control group)

**[SEEX]** Standard error (Treatment group)

**[SECON]** Standard error (Control group)

**[CORREX]** Correlation coefficient + *p* value (Treatment group)

**[CORRCON]** Correlation coefficient + *p* value (Control group)

**[SMDTREAT]** Standardised mean difference + confidence intervals

- Effect size: outcomes expressed in dichotomous data.

**[DSSTRE]** Sample size for the ES (Treatment group)

**[DSSCONT]** Sample size for the ES (Control group)

**[NUMTRE]** Treatment group; number of successful cases:

**[NUMCON]** Control group; number of successful cases:

**[PROPTRE]** Treatment group; proportion of successful cases:

**[PROPCON]** Control group; proportion of successful cases:

**[ORTRE]** Treatment group; odds ratios:

Confidence Intervals:

*p*-value:

**[ORCON]** Control group; odds ratios:

Confidence Intervals:

*p*-value:

**[ORADJ]** Are the odds ratios adjusted?

- 1. Yes.
- 0. No

Adjusted by (explain):_________

**[CHISC]** X^2^ value with *df:*

**[PAGEEFFECT]** Number of the page from where you extract statistical data:

- Effect sizes at follow-up

**[ESFOLLOW1]** Calculated effect at follows up 1:______

[ESFOLL1] Number of months after intervention for follow-up 1:______

**[ESFOLLOW2]** Calculated effect at follows up 2:______

[ESFOLL2] Number of months after intervention for follow-up 2:______

**[ESFOLLOW3]** Calculated effect at follows up 3:______

[ESFOLL3] Number of months after intervention for follow-up 3:______

**[ESFOLLOW4]** Calculated effect at follows up 4:______

[ESFOLL4] Number of months after intervention for follow-up 4:______

**Appendix E. Effect Size calculations**

Where outcomes were measured dichotomously, we computed odds ratios as an estimate of the treatment effect. Odds ratios (ORs) were estimated as:

$$OR= \frac{AD}{BC}$$

The variance of an odds ratio was estimated as:

$$V_{OR}= \frac{1}{A}+\frac{1}{B}+\frac{1}{C}+\frac{1}{D}$$

In the equation, A represents the number of students excluded in the treatment group and B is the number of students not excluded in the treatment group. In the formula, the number of students excluded or not excluded in the control group are represented by C and D, respectively. Typically, the number of students not excluded is not reported by primary evaluation, but calculated using the group sample size and the frequency or proportion of students who were excluded.

Where outcomes were measured as continuous measures we used Cohen’s *d* effect sizes to estimate treatment effects. Cohen’s *d* is estimated as:

$$d= \frac{\underline{x}_{2}-\underline{x}_{1}}{Pooled SD}$$

Where $\underline{x}_{2}$ and $\underline{x}_{1}$represent the mean of the outcome in the treatment and control groups respectively. The pooled standard deviation (SD) was estimated as:

$$Pooled SD= \sqrt{\frac{\left( n_{1}-1 \right)s_{1}^{2}+ \left( n_{2}-1 \right)s_{2}^{2}}{n_{1}+n_{2}-2}}$$

In the above formula, *n_1_* and *n_2_* were the sample sizes of treatment and control groups and *s_1_* and *s_2_* were the standard deviation for the outcome measured in the treatment and control groups respectively. The variance of *d* was estimated as:

$$v_{d}=\left( \frac{n_{1}+n_{2}}{n_{1}n_{2}}+ \frac{d^{2}}{2\left( n_{1}+ n_{2}-2 \right)} \right)\left( \frac{n_{1}+n_{2}}{n_{1}+n_{2}-2} \right)$$

We aimed to estimate difference-in-difference (DID) effect sizes to calculate the effect of interventions where possible. A DID Cohen’s *d* was estimated as the difference between groups after implementation of the intervention (i.e., post-test or follow-up) and the difference between groups before implementation of the intervention. Where baseline statistics were not reported we estimated post-test only effect sizes.

Included evaluations may not have reported descriptive information such as raw frequencies, means and standard deviations and group sample sizes in order to estimate either ORs or Cohen’s *d* effect sizes using the formulae previously outlined. An effect size calculator^^[[3]](#footnote-3)^^ (Wilson, n.d.) was used to estimate Cohen’s *d* effect sizes where results of impact evaluations were reported as F-tests from one-way ANOVAs or coefficients from regression models.

The present review conducted meta-analyses of multiple outcomes. In some instances where a decrease in the outcome was a desirable intervention effect (e.g., school exclusions, violence, or offending) the above formula for an odds ratio would result in an OR less than 1 indicating a positive impact of the intervention. However, in instances where an increase in the outcome is desirable, the formula would result in an OR greater than 1 indicating a positive impact of the intervention. Thus, we adjusted the direction of odds ratios for outcomes where a decrease is desirable. The natural logarithm scale (LnOR) will be used for all computations using odds ratios.

Additionally, included evaluations may have reported the impact of an intervention on a rare event. For example, being permanently excluded from school or engaging in violent offending. In such instances, values used to compute an odds ratio may include zero (i.e., no students were excluded, or no participants report violence). In these cases, we used an odds ratio continuity principle in order to compute an effect size. In these cases, 0.5 was added to each value in the 2x2 frequency table used to estimate the OR.

The weighted mean effect size in our meta-analysis was represented as Cohen’s *d,* or the standardised mean difference (SMD). Therefore, effect sizes estimated as ORs were transformed to Cohen’s *d* for the purpose of analysis. (Lipsey & Wilson, 2001a) formula for computing *d* from an odds ratio was used. This transformation is as follows:

$$LnOR= \frac{d}{0.5513}$$

**Appendix F. Narrative summaries of process evaluations**

*Allen et al. (2022)*

As part of a larger trial (i.e., the STARS trial; Ford et al., 2019) to evaluate the impact of the Incredible Years ® teacher classroom management programme (IY TCM) in Devon, UK, Allen et al. (2022) report the results of a process evaluation with teachers from eight primary schools that took part in the cluster-randomised controlled trial. The STARS trial evaluated the impact of the IY TCM programme on several outcome domains, including disruptive behaviour.

First developed in the US, the IY programme is described as a classroom management programme that can be implemented to help teachers reduce disruptive behaviour in their classrooms (Allen et al., 2022; Webster-Stratton et al., 2011). The programme is delivered over 6 sessions that take place over 6 months and has four main goals, to: (1) improve teachers’ skills in behaviour management and improve relationships between teachers and pupils; (2) support teachers to utilize proactive classroom management strategies; (3) encourage teachers to use social and emotional regulation skills; and (4) foster positive relationships between teachers and parents (Allen et al., 2022). During training sessions, that take over a whole day, teachers engage in reflective exercises, problem solving, role play and group discussion (Allen et al., 2022).

Allen et al. (2022) interviewed teachers that took part in IY training sessions two months after they completed the training. Training took place during the academic year and in groups of 8-12 teachers (Allen et al., 2022). Led by training group leaders, teachers took part in six sessions and the programme focused on: “… building positive relationships with students; preventing problem behaviour; teacher inattention; coaching and praise; motivating children through incentives; decreasing inappropriate behaviour; and emotional regulation, social skills and problem solving” (Allen et al., 2022, p. 1162; Webster-Stratton et al., 2011).

*Ashworth (2018); Humphrey et al. (2018)*

Both Ashworth (2018) and Humphrey et al. (2018) report qualitative findings from a process evaluation of the Good Behaviour Game. This intervention is described as a universal school-based intervention and relies on group-based intervention activities delivered in classrooms by trained teachers (Ashworth, 2018). There are four main components underlying the GBG: (1) classroom rules that outline expectations about student behaviour; (2) teamwork in groups of seven children with a designated team leader; (3) teachers monitor student behaviour, and students can also learn to monitor each other’s behaviour; and (4) positive reinforcement of desirable behaviours (Ashworth, 2018; Humphrey et al., 2018).

Teachers are provided with training prior to using the GBG in their classrooms and training sessions take place over two days (Ashworth, 2018). Training focuses on the theoretical background and presumed causal mechanisms, and teachers also receive on-going support and mentoring from a GBG coach (Ashworth, 2018). Coaching takes place every two to three weeks, and involves coaches observing teachers implementing the programme and providing teachers with constructive and actionable feedback (Humphrey et al., 2018).

The GBG is designed to be implemented in classrooms, and the aim of the game is to ensure that teams of students have less than five rule breaks. Rules for behaviour are set by individual teachers and the game is first used for 10 minutes, three times per week but this can increase to daily implementation for up to 30 minutes (Ashworth, 2018; Humphrey et al., 2018).

*Blanford-Elliott (2021)*

School-wide Positive Behaviour Support (SWPBIS) is a well-known and widely researched intervention. First developed in the US, Blandford-Elliott (2021) examined the implementation of this intervention in Welsh primary schools. As an example of applied behavioural analysis implemented on a large scale (i.e., across an entire school; Horner & Sugai, 2015), SWPBIS aims to promote pro-social behaviours in positive school environment.

A multi-faceted approach, SWPBIS is designed on a three-tier framework. Universal prevention strategies implemented with all members of a school community comprise the first tier and targeted intervention strategies with students demonstrating behavioural problems are encompassed in the second tier (Blandford-Elliott, 2021). The third tier involves further intensive support for students requiring additional help despite partaking in tiers one and two (Blandford-Elliott, 2021).

Schools that implemented SWPBIS and evaluated by Blandford-Elliott (2021) engaged in various intervention activities. Participating schools created a SWPBIS committees of staff members to oversee the implementation and decide on a value system for their school (Blandford-Elliott, 2021). Committees met with researchers twice termly. This is a train-the-trainer model, where teachers and school staff are trained by a trained facilitator, and then implement training and intervention activities in their respective schools. Additional or follow-up training was provided by researchers if requested by schools (Blandford-Elliott, 2021).

*Middleton (2022)*

This process evaluation examined the implementation of programmes informed by ‘nurture practice’, a form of trauma-informed intervention based on six principles: (1) transitions; (2) learning; (3) behaviour; (4) language; (5) wellbeing; and (6) safety (Middleton, 2022). Based on theoretical frameworks relating to attachment, child development, and the effect of early adverse childhood experiences, nuture practice was incorporated into Violence Reduction Unti programmes in London and Medway (Middleton, 2022). The fundamental concept informing programmes using nurture practice is the aim to provide a safe space for children who have experienced disruption or adversity in life (Boxall, 2010; Lucas, 2019). The approach aims also to ‘bridge the gap’ between a child’s home and school life and tries to recreate core experiences that may have been missed in early child development through supportive and trained teachers and school staff (Boxall, 2010; Education Scotland, 2018; Middleton, 2021; 2022).

The programmes evaluated by Middleton (2022) are described as being based on the six core principles of nuture practice, and offered schools:

1. Tailored consultancy with an experienced nurtureUK consultant to review schools’ existing approaches, progress meetings and specific support.
2. Training for school staff in nurture programmes and approach.
3. Evidence-based assessment using data from individual schools to guide their progress.
4. Products and resources to support nurture practice.
5. Partnership programmes to deliver training, support staff and regular networking meetings to connect participating schools.

Middleton (2022) conducted focus groups with project leaders that led school-based restorative and nurturing programmes between 2020 and 2022. Nine participants took part, including senior leadership from mainstream secondary schools and alternative provision institutions. Participants were drawn from a pool of 31 educational settings across London boroughs and nine secondary schools in Kent and Medway (Middleton, 2022).

*Sparling et al. (2022)*

The ACE-informed schools (AIS) model is a trauma-informed intervention and was evaluated in 13 primary schools in England by Sparling et al. (2022). ACEs are events during childhood that could possibly be traumatic and have a long-term negative impact on outcomes later in life (Sparling et al., 2022). Examples include, sexual, physical or emotional abuse, witnessing domestic violence, or abandonment by a caregiver.

Sparling et al. (2022) examined the implementation of the AIS intervention, which involved training sessions for school staff designed by educational psychologists. Training was delivered over three sessions and included content related to research on ACEs, attachment theory, developmental trauma, increasing resilience and behaviour management strategies (Sparling et al., 2022). Following training, participants were provided with a ‘toolkit’ containing ideas for using trauma-informed approaches in their daily professional life. School leaders were also asked to revise school systems that could support the AIS model and create an action plan with clear goals and targets for integrating trauma-informed models into the school environment (Sparling et al., 2022). Additional support from intervention facilitators, such as educational psychologists, was available but schools were encouraged to adapt the AIS model to their own respective school communities (Sparling et al., 2022).

*Stanbridge & Campbell (2016)*

The intervention planning tool evaluated by Stanbridge and Campbell (2016) aimed to help schools support the emotional well-being and behaviour of students. The intervention planning tool was grounded in many principles, including, multi-element plans (MEPs) and target-monitoring and evaluation (TME; Stanbridge and Campbell, 2016). MEPs are described as collaborative approaches to implement multiple interventions, targeting a range of factors, to support behaviour and well-being of students (Stanbridge & Campbell, 2016). The intervention planning tool was also developed to recognize that students in the UK are typically only offered one intervention programme, but that these interventions need to consider the role of the school environment and the function that a behaviour may serve (i.e., functional behaviour assessments; Stanbridge & Campbell, 2016). TME is incorporated into the tool to support students further by creating clearly defined targets and a process for monitoring progression (Stanbridge & Campbell, 2016).

Stanbridge and Campbell (2016) obtained the views of staff members from two schools that had been trained to use and implement the intervention planning tool. The intervention involved group discussion and meetings between members of staff to effectively discuss a students’ behaviour and to try and understand the possible motivations for said behaviour(s). Students’ views were obtained before meetings and the purpose of meetings was to set out a plan for addressing problem behaviours, create targets and design a process to monitor student progression (Stanbridge & Campbell, 2016).

Twelve students took part in the intervention from two primary schools in a rural area. The majority were identified as male (*n* = 8) and they were aged 8 to 11 years. Students were identified to take part by school staff and were those students for whom staff had concerns about their emotional well-being but perhaps they did not meet the criteria for additional support such as special educational needs support (Stanbridge & Campbell, 2016). Information regarding student ethnicity is not provided.

Nineteen individuals, students, and school staff were interviewed for the process evaluation and no demographic information about these participants (Stanbridge & Campbell, 2016).

*Warren et al. (2020)*

Warren et al. (2020) report the findings from a process evaluation of the Learning together intervention, a heath-based programme, implemented and evaluated in a three-year cluster randomised controlled trial. As a whole-school intervention, the programme encompasses a number of different components, such as, a manual, classroom curriculum, and restorative practice training (Warren et al., 2020). The programme also involves a yearly survey of students to investigate the needs and views of students on their health (Warren et al., 2020).

Training is provided by trained facilitators and is delivered to all staff in the first year, in sessions lasting between two and three hours (Warren et al., 2020). Training in restorative practice is delivered by an external specialist organisation. In subsequent years, a 3-day training event is offered and schools may select between five and ten members of staff to attend (Warren et al., 2010). Training involves group discussions, group work and role play activities. Teachers are trained to implement restorative conferences with their students to address occurrences of bullying or aggression, and based on the seriousness of the behaviour, parents and/or the police may also be involved (Warren et al., 2020).

At the school-level, schools are required to create action groups of staff members and students who meet regularly (six times per year) to review the findings of yearly surveys about students’ health needs, make decisions about strategies to address the needs highlighted (Warren et al., 2020). The action group is also responsible for the implementation of the social and emotional learning curriculum and reviewing and revising existing school policies (Warren et al., 2020).

Warren et al. (2020) examined perspectives of participants using both focus groups and interviews with staff and students from three case study schools in London. Two of the schools are described as high deprivation, with high rates of students eligible for free school meals and students for whom English was an additional language. The third school was described as being in a more affluent area of London with relatively low levels of problem behaviours (Warren et al., 2020). No demographic information about the participants is provided.

*Wright (2020)*

Wright (2020) evaluated the impact of the Risk-Advert programme, alongside the Risk-Advert screening tool. Whilst the screening tool focuses on identifying pupils engaged in ‘risky behaviour’, the Risk-Advert programme is a teacher-led group intervention that aims to “… encourage[s] young people to analyse the why for their behaviour and what motivates them” (Wright, 2020, p. 159). Intervention sessions focus on moving away from the consequences of risky behaviour, and to instead reduce risk by facilitating insight into pupils’ behaviour, motivations, and decision-making (Wright, 2020). Two decision-making models are taught to participants; the ‘Four Whats’ and the Traffic Light system.

The ‘Four Whats’ decision-making process is represented visually by a set of five mechanical cogs. The aim is to demonstrate to students that the factors and elements contributing to risky behaviour are related (Wright, 2020). The following provides a brief overview of each cog:

1. ‘What I know’: This symbolizes what the participants knows about their behaviour and the consequences.
2. ‘What I feel’: This symbolizes the possible feelings around a particular behaviour and encompasses both simple (e.g., fear or excitement) and complex (e.g., sense of obligation) feelings.
3. ‘What I want’: This symbolizes the participants desires and priorities for their lives in general.
4. ‘What I do’: This symbolizes the interaction of cogs 1 to 3, and how they influence the participants’ behaviour.
5. ‘Why I do it’: This symbolizes the reasons for engaging in a particular behaviour, and provides an explanation for cog 4.

The second model is the Traffic Light system, where participants are taught to adapt their thought process before engaging in a perceived risky behaviour. The ‘red light’ represents the opportunity to stop and reflect; for a young person to identify a behaviour as risky and reflect upon what the risk is and why it is risky (Wright, 2020). The ‘yellow light’ represents the opportunity to consider the ‘pros and cons’ of the behaviour and reflect upon their feelings about the risk (Wright, 2020). Finally, the ‘green light’ represents the decision to engage in the behaviour or to refrain.

Wright (2020) interviewed a convenience sample of teachers at two schools in southern England that took part in the Risk-Advert programme. Nine members of staff from eight schools took part in the process evaluation undertaken by Wright (2020), recruited through email requests for participation sent to all schools implementing the programme across Suffolk and Essex. All participants that were interviewed had led sessions as part of the Risk-Advert programme in 2016/17 and the majority were in student support roles (Wright, 2020).

**APPENDIX G. PROCESS EVALUATIONS EXCLUDED AT DATA EXTRACTION**

*Calcutt (2021)*

Calcutt (2021) conducted a type of ethnographic study, whereby, they investigated the implementation of mindfulness lessons in their Year 3 classroom. It was excluded as the findings were not relevant enough to student misbehaviour that may lead to a child being excluded from school. The research used a social justice framework and used case study methods, guided by the overarching research question to understand the successful implementation of mindfulness. The majority of findings were focussed on student and facilitator enjoyment with the mindfulness activities and the impact on student learning (Calcutt, 2021). There were some findings on student behaviour, such as, the researcher noting that mindfulness techniques were often useful in settling “a restless class” (Calcutt, 2021, p. 116). Overall, the students enjoyed the programme, with some identifying that teachers would benefit from the programme also. Calcutt (2021) outlines, “Children noted that teachers would enjoy mindfulness because it improved the behaviour of the class – the class would be “nice and quiet”.” (Calcutt, 2021, p. 117). Finally of note, Calcutt (2021) found that some elements of mindfulness were useful to apply when a child got angry or upset, even if the particular child had not participated in mindfulness themselves. However, this process evaluation was excluded from our qualitative evidence synthesis as, given the setting and context, the findings were primarily focussed on “increased attention and focus” (Teaching assistant; Calcutt, 2021, p. 116).

*Knowler et al. (2019)*

At first glance, the process evaluation by Knowler et al. (2019) seemed to be the best fit to our inclusion criteria for the qualitative evidence synthesis. This evaluation investigated the implementation of a programme for children at-risk of permanent exclusion from school. However, the programme was defined as an outdoor learning experience whereby children spent time away from normal school activities and therefore does not meet our inclusion criteria. Knowler et al. (2019) used a visual content analysis approach to analyse images captured during the implementation of the outdoor learning programme. The findings were interesting and showed that the outdoor learning experience could have an impact on children at-risk of exclusion. For example, children who participated were observed in “complete absorption in learning” (Knowler et al., 2019, p. 3991), something which the authors is not characteristically typical of this sample. Children at-risk of exclusion are generally seen to have problems with attention and task completion, and so the outdoor learning environment may have been beneficial (Knowler et al., 2019). Whilst a full overview of the results is beyond the scope of the current review, it is noteworthy to mention that Knowler et al. (2019) also found that children at-risk of exclusion who took part in the programme appreciated the opportunity to be creative, new and novel ways to learn, and the decrease in formal adult supervision in the outdoor learning environment.

*Marchant et al. (2019)*

Marchant et al. (2019) evaluated a similar programme, where participating schools in Wales implemented outdoor learning into regular curriculum teaching. Participants were interviewed by researchers and took part in focus groups to understand the facilitators and barriers to implementation. The findings were interesting, and participants, students and teachers alike viewed the intervention favourably, but the study was excluded from our qualitative evidence synthesis. There was some discussion of the impact of the programme on student behaviour, but similar to Calcutt (2020), behaviour in this sense related to more general classroom behaviour and not disruptive or aggressive behaviours that may increase the risk of being excluded.

*Rivers (2016)*

An evaluation by Rivers (2016) was excluded from the qualitative evidence synthesis during data extraction as the findings were not sufficiently relevant to behaviour considered related to children being excluded from school. This intervention involved bibliotherapy and used a mixed-methods approach. The quantitative results included an outcomes (i.e., L3 behaviour sanctions) that included exclusionary discipline. However, the study was excluded from our synthesis of process evaluations. This was because the qualitative findings focussed on students’ learning behaviours and attitudes towards reading and literacy (Rivers, 2016).

*Reynolds (2021)*

An evaluation by Reynolds (2021) was excluded from the qualitative evidence synthesis at the data extraction phase. This was a mixed-methods study of referral to internal inclusion units (IIUs) and as such, did not meet our inclusion criteria for interventions to reduce school exclusions. It is our view that these IIUs are a form of alternative provision. In qualitative interviews with six participants, Reynolds (2021) examined perceptions of the IIUs and student’s views on whether or not this strategy improved their behaviour. Overall, whilst students reported that the IIUs did positively impact their behaviour and they perceived it as a worthwhile facility, they also reported feelings of neglect and rejection from school staff and commented on the unfairness and unjust nature of the system (Reynolds, 2021).

**APPENDIX H: CASP CHECKLIST RESULTS**

| **Checklist question** | **Study** | | | | | | | | |
| --- | --- | --- | --- | --- | --- | --- | --- | --- | --- |
|  | Allen et al. (2020) | Ashworth (2018) | Blandford-Elliott (2021) | Humphrey et al. (2018) | Middleton (2022) | Sparling et al. (2022) | Stanbridge & Campbell (2016) | Warren et al. (2020) | Wright (2020) |
| 1. Was there a clear statement of the aims of the research? | Yes | Yes | No | Yes | Yes | Yes | Yes | Yes | No |
| 2. Is a qualitative methodology appropriate? | Yes | Yes | Yes | Yes | Yes | Yes | Somewhat | Yes | Yes |
| 3. Was the research design appropriate to address the aims of the research? | Somewhat | Yes | Yes | Yes | Yes | Somewhat | Somewhat | Yes | Yes |
| 4. Was the recruitment strategy appropriate to the aims of the research? | No | Yes | No | Yes | Yes | No | Yes | Yes | Yes |
| 5. Was the data collected in a way that addressed the research issue? | Yes | Yes | Yes | Yes | Yes | Yes | Somewhat | Yes | Yes |
| 6. Has the relationship between researcher and participants been adequately considered? | Yes | No | Yes | No | No | No | No | No | No |
| 7. Have ethical issues been taken into consideration? | Yes | Yes | Yes | Yes | Yes | Yes | No | Yes | Yes |
| 8. Was the data analysis sufficiently rigorous? | Yes | Yes | Somewhat | Yes | Yes | No | No | Yes | Yes |
| 9. Is there a clear statement of findings? | Yes | Yes | Somewhat | No | Yes | Yes | Yes | Yes | Yes |
| 10. How valuable is the research? | Yes | Yes | Somewhat | Yes | Yes | Yes | Somewhat | Yes | Somewhat |

1. Based on Lipsey & Wilson (2001) [↑](#footnote-ref-1)
2. Opposition/defiance, physical aggression, indirect aggression, instrumental aggressions/dominance, reactive aggression, school bullying, antisocial behaviour or cime, are expected to be captured in the variable AAGRESEXT. [↑](#footnote-ref-2)
3. [https://campbellcollaboration.org/research-resources/effect-size-calculator.html](https://www.campbellcollaboration.org/research-resources/effect-size-calculator.html) [↑](#footnote-ref-3)
